# First-In-Class CD13-Targeted Tissue Factor tTF-NGR in Patients with Recurrent or Refractory Malignant Tumors: Results of a Phase I Dose-Escalation Study

**DOI:** 10.3390/cancers12061488

**Published:** 2020-06-07

**Authors:** Christoph Schliemann, Mirjam Gerwing, Hauke Heinzow, Saliha Harrach, Christian Schwöppe, Moritz Wildgruber, Anna A. Hansmeier, Linus Angenendt, Andrew F. Berdel, Ursula Stalmann, Björna Berning, Karsten Kratz-Albers, Kristina Middelberg-Bisping, Stefanie Wiebe, Jörn Albring, Christian Wilms, Wolfgang Hartmann, Eva Wardelmann, Tobias Krähling, Walter Heindel, Joachim Gerss, Eike Bormann, Hartmut Schmidt, Georg Lenz, Torsten Kessler, Rolf M. Mesters, Wolfgang E. Berdel

**Affiliations:** 1 Department of Medicine A, Hematology, Oncology, University Hospital Muenster, D-48149 Muenster, Germany; Christoph.Schliemann@ukmuenster.de (C.S.); s.harrach@gmx.net (S.H.); christian.schwoeppe@uni-muenster.de (C.S.); Anna.Hansmeier@ukmuenster.de (A.A.H.); linus.angenendt@ukmuenster.de (L.A.); andrew.berdel@ukmuenster.de (A.F.B.); u.stalmann@erasmusmc.nl (U.S.); JoernChristian.Albring@ukmuenster.de (J.A.); georg.lenz@ukmuenster.de (G.L.); Torsten.Kessler@ukmuenster.de (T.K.); Rolf.Mesters@ukmuenster.de (R.M.M.); 2 Institute for Clinical Radiology, University Hospital Muenster, D-48149 Muenster, Germany; Mirjam.Gerwing@ukmuenster.de (M.G.); Moritz.wildgruber@med.uni-muenchen.de (M.W.); tobias.kraehling@uni-muenster.de (T.K.); heindel@uni-muenster.de (W.H.); 3 Department of Medicine B, Gastroenterology, University Hospital Muenster, D-48149 Muenster, Germany; Hauke.Heinzow@ukmuenster.de (H.H.); Christian.Wilms@ukmuenster.de (C.W.); hepar@ukmuenster.de (H.S.); 4 Oncology Practice, D-48431 Rheine, Germany; bjoerna.berning@icloud.com; 5 Group Practice Hematology Oncology, D-48149 Muenster, Germany; kratzalbers@onkologie-muenster.de; 6 Department of Medicine, Marienhospital Osnabrück, D-49074 Osnabrück, Germany; kristina.middelberg-bisping@mho.de; 7 Department of Hematology Oncology, St. Franziskus Hospital, D-48145 Muenster, Germany; Stefanie.Wiebe@SFH-MUENSTER.de; 8 Gerhard-Domagk-Institute for Pathology, University of Muenster, D-48149 Muenster, Germany; Wolfgang.Hartmann@ukmuenster.de (W.H.); Eva.Wardelmann@ukmuenster.de (E.W.); 9 Institute of Biostatistics and Clinical Research, University of Muenster, D-48149 Muenster, Germany; Joachim.Gerss@ukmuenster.de (J.G.); Eike.Bormann@ukmuenster.de (E.B.)

**Keywords:** tTF-NGR, vascular targeting, CD13, aminopeptidase N, first-in-class phase I study

## Abstract

Background: Aminopeptidase N (CD13) is present on tumor vasculature cells and some tumor cells. Truncated tissue factor (tTF) with a C-terminal NGR-peptide (tTF-NGR) binds to CD13 and causes tumor vascular thrombosis with infarction. Methods: We treated 17 patients with advanced cancer beyond standard therapies in a phase I study with tTF-NGR (1-h infusion, central venous access, 5 consecutive days, and rest periods of 2 weeks). The study allowed intraindividual dose escalations between cycles and established Maximum Tolerated Dose (MTD) and Dose-Limiting Toxicity (DLT) by verification cohorts. Results: MTD was 3 mg/m^2^ tTF-NGR/day × 5, q day 22. DLT was an isolated and reversible elevation of high sensitivity (hs) Troponin T hs without clinical sequelae. Three thromboembolic events (grade 2), tTF-NGR-related besides other relevant risk factors, were reversible upon anticoagulation. Imaging by contrast-enhanced ultrasound (CEUS) and dynamic contrast-enhanced (DCE) magnetic resonance imaging (MRI) showed major tumor-specific reduction of blood flow in all measurable lesions as proof of principle for the mode of action of tTF-NGR. There were no responses as defined by Response Evaluation Criteria in Solid Tumors (RECIST), although some lesions showed intratumoral hemorrhage and necrosis after tTF-NGR application. Pharmacokinetic analysis showed a t_1/2(terminal)_ of 8 to 9 h without accumulation in daily administrations. Conclusion: tTF-NGR is safely applicable with this regimen. Imaging showed selective reduction of tumor blood flow and intratumoral hemorrhage and necrosis.

## 1. Introduction

The formation of a tumor neo-vasculature is important for nutrient and oxygen supply and for removal of metabolic waste and, thus, is essential for support of tumor growth and spread [1]. To complement antiangiogenic approaches in cancer therapy, Denekamp et al. proposed existing tumor vessels and tumor endothelial cells as target carriers for anti-vascular therapy [2]. The tumor vasculature indeed presents therapeutic targets for antiangiogenic therapies, vascular disruption, or vascular occlusion and thrombosis with subsequent tumor infarction. Thorpe et al. introduced the concept of tumor vessel occlusion by targeted Tissue Factor (TF) [3]. Pasqualini et al. characterized small NGR (asparagine-glycine-arginine)-containing peptides, binding to aminopeptidase N (APN, also known as CD13) as a tumor vascular target [4]. CD13 [5,6] has been shown to promote angiogenesis, tumor growth, and metastasis [7] and has also been shown to be of prognostic relevance for patients with cancer of some but not all histologies examined [8,9,10,11,12].

We have designed and studied a new class of fusion proteins carrying NGR peptides instead of the transmembrane domain at the C-terminus of truncated tissue factor (tTF) to induce tumor vascular thrombosis and occlusion with subsequent infarction [13,14,15,16,17,18,19,20]. These fusion proteins were tested in vitro and in vivo for essential therapeutic properties, such as pro-coagulatory activity, specific binding to their respective target molecules on stimulated endothelial cells (EC) or pericytes, in vivo intratumoral accumulation, in vivo intratumoral activation of coagulation, tumor vascular occlusion and inhibition of blood flow, pharmacodynamic properties including therapeutic antitumor activity in xenotransplants of human tumors from different histologic origin, and finally rodent as well as non-rodent animal safety and toxicology (Investigator’s Brochure (IB), authors on file) [13,14,15,16,17,18,19,20]. The results of the first-in-class phase I trial of the lead protein tTF-NGR in late-stage cancer patients are presented here.

## 2. Patients and Methods

### 2.1. Good Manufacturing Practice (GMP) Production of tTF-NGR

GMP production of clinical scale tTF-NGR was performed in our GMP facility under a manufacturer´s authorisation (DE_NW_05_MIA_2016_0011/24.05.03.-034/2015.0002) by the regional government and the federal Paul-Ehrlich Institute (PEI; Langen, Germany; No. 2852/01). The detailed procedure is described in an Investigational Medicinal Product Dossier (IMPD on file). Cloning, expression in *E. coli,* and purification by a 4-step Preparative High-Performance Liquid Chromatography (HPLC unit: ÄKTA purifier 100 System, GE healthcare, Uppsala, Sweden) have been published before [15]. Quality control besides standard evaluations included testing of the investigational medicinal product (IMP) His-tag-tTF_1-218_-GNGRAHA (tTF-NGR) in a validated Factor X-activation assay for pro-coagulatory activity.

Clinical charges were taken for animal safety and toxicology testing according to ICH S6 and S9 guidelines by EMA and FDA before the start of this study (data in IMPD/IB on file).

### 2.2. Study Design and Patients

This was an investigator-initiated trial without support from the pharmaceutical industry. The study protocol (NCT02902237, EudraCT-No.: 2016-003042-85) in its version 10 is attached to this report (see the Supplementary Information). During study conduct, two amendments were introduced, the first adding dynamic contrast-enhanced ultrasound (CEUS) to dynamic contrast-enhanced (DCE) magnetic resonance imaging (MRI) as a second imaging method to determine the tumor perfusion within the secondary objectives of the trial and the second allowing for faster dose escalation in 1.0 mg/m^2^ steps between 3.0 and 5.0 mg/m^2^, at which dose-limiting toxicity (DLT) was observed. In addition, written informed consent forms were adapted when the new EU data safety legislation (EU-GDPR) was activated. The study protocol and amendments were approved by the Ethical Board of the Physician´s chamber of Westphalia-Lippe and the Westphalian Wilhelms University of Muenster (AZ 2016-414-f-A) and by the PEI. The study was performed according to the Declaration of Helsinki (Fortaleza, 2013), and written informed consent by the patients was obligatory prior to entry to the study.

In brief, the study was an open-label, single-arm, non-randomized, prospective, single-center phase I trial for patients with recurrent or refractory malignant tumors or lymphomas beyond all standard treatments. tTF-NGR was given under close in-house observation in our phase-I unit as a 1-h infusion in 0.9% NaCl (50 mL, later 100 mL) via central venous access once daily for 5 days with a subsequent rest period of 2 weeks and following cycles with intraindividual dose escalation of 0.5 mg/m^2^ (later 1 mg/m^2^) upon judgement of tolerability and therapeutic activity. Starting dose was 1 mg/m^2^/day. Patients within the dose escalation part were treated in sequence and not in parallel. Individual patients could theoretically undergo a maximum of 8 dose escalations. Dose escalation had to be stopped before the maximum number of 8 escalation steps if tumor response, tumor progression, or a DLT were observed. In the case of response or stable disease (SD) and with good tolerability, these patients could obtain further cycles without dose escalation until tumor progression or DLT and the next patient could start with dose-escalation cycles on the highest tolerable dose for the previous patient. The study first followed an intraindividual dose-escalation procedure as described, and after DLT was observed, introduced verification cohorts of 6 patients and a further de-escalation procedure when >1 DLT per 6 patients occurred in the verification cohort. Maximum Tolerated Dose (MTD) was defined as the highest dose with <2 DLT per 6 patients treated with 2 cycles as described. Primary objectives of the study were to establish the MTD and to characterize the DLT for tTF-NGR using this regimen. Secondary objectives were to determine the perfusion and vascular volume fraction (VVF) of measurable tumor lesions versus normal reference organ tissue before and after tTF-NGR application by DCE-MRI and CEUS as surrogate parameters for biological activity and proof of principle for tTF-NGR mode of action (MOA) as well as to obtain pharmacokinetic (PK) data for tTF-NGR and data on antitumor activity as defined by RECIST (Response Evaluation Criteria in Solid Tumors) criteria. Inclusion criteria were age ≥18 years; histologically proven or cytologically confirmed solid malignant tumor or malignant lymphoma; recurrent or refractory disease after standard therapy with no known curative or survival-prolonging treatment options according to the judgement of the investigators; life expectancy of at least 6 weeks according to the judgement of the investigators; Karnofsky performance status ≥50; measurable disease with at least one marker-lesion measurable by DCE-MRI or CEUS; adequate bone marrow function and laboratory values as detailed in the protocol attached (Supplementary Information; laboratory limits could be overruled with note to file by the investigator upon individual judgement); no history of coronary heart disease, stroke, transitory ischemic attacks, pulmonary embolism, or deep vein thrombosis; time elapsed from previous therapy (including other IMPs) ≥3 weeks with recovery from side effects; exclusion of central nervous system (CNS) disease and CNS vascular abnormalities by MRI; ability to understand and provide written informed consent; written informed consent given; and for female patients with child-bearing potential, exclusion of pregnancy by adequate testing within 48 h prior to entry on study. Females of childbearing potential as well as fertile males had to agree to use a highly effective form of contraception (Pearl index <1) during the study and for 120 days following the last dose of the IMP. Exclusion criteria comprised clinically significant unrelated illnesses which, in the judgement of the investigator, could compromise the patient’s ability to tolerate the investigational medicinal product (IMP) or could be likely to interfere with the study procedures or results; known hypersensitivity reactions to prior application of *E. coli-*derived material; women with breast-feeding activity; concomitant use of any other investigational agent; clinical application of any other drug with known antitumor activity; and prophylactic anticoagulation within the last 3 days. Study treatment was given in-house under monitoring. Pharmacokinetics and clinical and blood value monitoring were scheduled daily on multiple time points during treatment (Supplementary Information) and weekly after discharge of the patients on an out-patient basis. Adverse event (AE) reporting was according to Good Clinical Practice (GCP) guidelines (Supplementary Information). Common Terminology Criteria for Adverse Events (CTCAE) version 4.0 were used.

Since this was a single-arm dose-escalation phase I trial, only descriptive statistics were used for analysis of the main study. Statistical methods used for the hypothesis-generating single-parameter correlations are given in the Results section.

### 2.3. Dynamic Contrast-Enhanced Ultrasound (CEUS)

Dynamic CEUS (Logiq E9; GE Healthcare, Chalfont St Giles Buckinghamshire, UK) was performed after injecting 2.4 mL of sulfur-containing microbubbles (second-generation contrast agent SonoVue, Bracco Imaging) via the left cubital vein followed by an 8-mL saline flush. Images were acquired continuously within 90-s measuring influx, accumulation, and efflux through a preselected region of interest (ROI) by ultrasound. ROIs were selected by a single person (H.H.) within a metastasis and an adjacent area of normal tissue, e.g., within the liver. This person also performed the follow-up measurements. Echo-power was recorded over time and evaluated with VueBox^®^ Quantification Toolbox v.6.0 and v.7.2 software (BRACCO Suisse S.A. software applications, Manno, TI, Switzerland). Evaluation was done for “wash-in area under the curve” (WiAU) and/or “wash-in perfusion index” (wash-in area under the curve/rise time (WiAUC/RT)) as shown in Supplementary Figure S1.

### 2.4. Dynamic Contrast-Enhanced (DCE) Magnetic Resonance Imaging (MRI)

MRI of target lesions was performed with a 1.5 Tesla whole body scanner (Philips Achieva; Philips Healthcare, Best, The Netherlands). For assessment of tumor vascularity, Vascular Volume Fraction (VVF) was determined upon T2* mapping following injection of Resovist^®^ (Nihon Schering, Japan) as previously described [14]. In brief, R2* (1/T2*) values were used to calculate the VVF as followed: ΔR2*_tumor_/ΔR2*_muscle_. Note that measurement of VVF is normalized on muscle tissue. Additionally, tumor perfusion was quantified by calculation of K_trans_, which reflects the combined effects of plasma blood flow, permeability, and capillary surface area following injection of Gadobutrol^®^ (Bayer, Germany). Image analyses was performed on a commercial platform (IntelliSpace Portal 9.0, Philips Healthcare). Similar protocols have been published before [14,21]. Whenever K_trans_ of normal tissue adjacent to the tumor was measured, the ROI is marked in the Figures.

### 2.5. Pharmacokinetic Determination of tTF-NGR in Human Plasma

An enzyme-linked immunosorbent assay (ELISA) was used for quantitative determination of tTF-NGR levels in human plasma in a modification of a method described [16]. To this effect, the commercially available IMUBIND^®^ Tissue Factor ELISA Kit (American Diagnostica, Stamford, CT, USA) was validated to present objective evidence that this method fulfills the requirements for its intended use in the clinical phase I study of tTF-NGR. The analytical method was validated with regard to accuracy, precision (repeatability and intermediate precision), as well as long-term and freeze-thaw stability (full validation report in the IMPD on file).

Pharmacokinetic (PK) evaluation of the plasma data was performed using PKSolver, a freely available menu-driven add-in program for Microsoft Excel. A non-compartment model was employed. The following parameters were determined:

AUC_0–t last_ = extrapolated area from time zero to the last quantifiable plasma concentration
K_el_ = elimination rate constant
t_1/2_ = alpha (distribution phase), terminal (elimination phase) half-life

C_max_ values represent the highest measured plasma concentrations, while t_max_ values represent the time-points of the highest plasma concentrations. AUC_0–t last_ was calculated according to the linear trapezoidal rule. The dose proportionality of C_max_ and AUC was evaluated. Elimination rate constants (K_el_) and plasma elimination half-lives (t_1/2_) were calculated by linear regression analysis of the log/linear portion of the individual plasma concentration-time curves (c = concentration, t = time, and h = hours). Half-life is defined as follows:

(1) $$t_{1/2}=\frac{\ln2}{K_{\mathrm{el}}}[h]$$

(2) $$\frac{\mathrm{dc}}{\mathrm{dt}}=K_{\mathrm{el}}\times c[h]$$

The C_max_ levels and AUC areas revealed a dose-related exposure of the patients to tTF-NGR during the treatment period. The PK parameters were based on tissue plasma factor concentration measured by ELISA assay after the first dose (initial PK assessment).

### 2.6. Human Anti-Fusion Protein Antibody (HAFA) Determination

Presence of antibodies to tTF-NGR was determined using an ELISA assay (detailed description, IMPD on file). Briefly, in the screening assay, the tTF-NGR was captured onto the plate by histidine-tag antigen and then the patient’s sera were diluted and applied into microplate wells; if antibodies (IgG) to tTF-NGR were present in the serum, they bind to tTF-NGR. A peroxidase-conjugated antihuman IgG was added, forming a complex with the human anti-tTF-NGR antibodies, and the probes were then developed by adding the peroxidase substrate to produce a visible signal. The optical density measurements (OD_450nm–650nm_) were performed using a standard microplate reader. The results were evaluated by using a pretreatment cutoff value: samples producing a signal at or above the cutoff point were considered positive; otherwise, they were reported as negative.

## 3. Results

### 3.1. Patients Demographics and Disease Characteristics

Twenty-four patients were screened, of which 6 were screening failures and not treated (Unique Patient Number (UPN) 001: fast disease progression—decision by investigator; UPN 002: retraction of consent by patient; UPN 010, 013, and 014: fast disease progression—decision by investigator; and UPN 017: new brain metastasis diagnosed in screening MRI). One patient (UPN 016) died from sepsis shortly before treatment with tTF-NGR could be started. Thus, 17 patients (Caucasian origin) obtained at least 1 cycle of tTF-NGR and were eligible for safety and toxicity evaluation and for efficacy evaluation (characteristics in Table 1). Median age of these patients was 54 years (range 19–74), with 4 being female. We treated patients with various malignant tumor entities during dose escalation and enriched for sarcoma patients within the verification cohorts, since soft tissue sarcomas (STS) have high CD13 target expression [20].

### 3.2. Safety and Tolerability 

**Figure 1:**
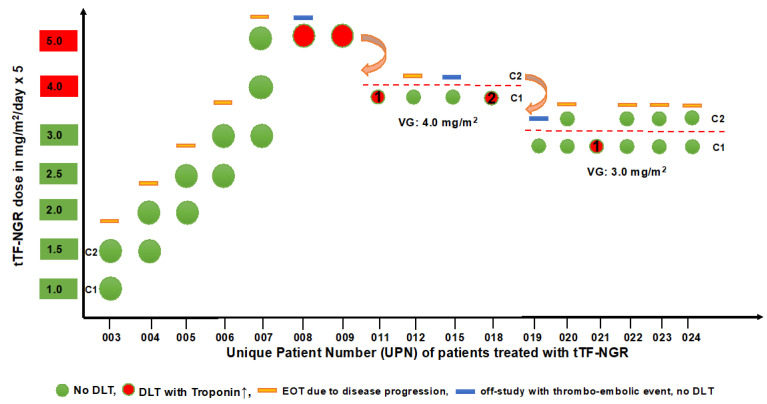
Distribution of all patients treated and cycles of tTF-NGR given over the dose range studied. UPN, unique patient number; for further information, see Table 1; C, cycle; EOT, end of treatment; DLT, dose limiting toxicity; VG, verification group.

Figure 1 depicts the dose levels and cycles for all patients treated with tTF-NGR (Figure 1, see also Table 1). During dose escalation, 4 patients obtained 2 cycles and 1 patient obtained 3 cycles. There was no DLT, and tolerability of tTF-NGR was good. A listing of all adverse events (AE) observed is given in Supplementary Table S1. Absolute doses of tTF-NGR given per patient and day are depicted in Supplementary Table S2. Subsequently, treatment had to be stopped in these patients due to disease progression. The second patient on dose level 5 mg/m^2^ (UPN 008, synovial sarcoma) showed a DLT after 2 applications of tTF-NGR as an isolated increase of Troponin T hs (hs, high sensitivity) to levels >50 ng/L. Although CTCAE 4.0 does not include high-sensitivity Troponin T hs, we have judged this increase as being grade 3 according to CTCAE laboratory value criteria for cardiac Troponin (“consistent with myocardial infarction”, normal range <14 ng/L, and grey area 14–50 ng/L). However, no cardiac symptoms occurred or developed later, and ECG and all other cardiac lab values were nonindicative for myocardial hypoxia or infarction. Echocardiography ruled out changes compatible with myocardial infarction or pulmonary embolism, thoracic angio-CT ruled out pulmonary embolism, and the Troponin T hs values upon reaching a peak value of 197 ng/L after 4 days decreased over the next days without further tTF-NGR application under anticoagulation with enoxaparin and acetylsalicylic acid (ASS). Isolated Troponin T hs elevation without any clinical sequelae occurred also in the next patient (UPN 009, non-small cell lung cancer) after 1 application of tTF-NGR (maximum value 209 ng/mL on day 1 after application with decrease under enoxaparin and ASS); this patient however already had an elevated Troponin T hs value before start of tTF-NGR. Both events were judged as Suspected Unexpected Serious Adverse Reactions (SUSARs; related to the IMP, resolved/recovered) and DLT. In addition, an isolated Troponin T hs elevation occurred also in 2 other patients: UPN 004 reached 50.7 ng/L on day 2 of 2.0 mg/m^2^ tTF-NGR, but values normalized under therapy already on day 3 of treatment. Thus, we have interpreted this increase as no AE and as not related to tTF-NGR. UPN 005 showed elevated values at 2.5 mg/m^2^ tTF-NGR. This was also interpreted as not being related to the IMP, since the patient had elevated levels before treatment and under repeated intrabronchial laser therapy to shrink tumor deposits and to stop tumor bleeding with inflammatory reaction.

Subsequently, the first verification cohort was opened by de-escalating the dose of tTF-NGR to 4 mg/m^2^ according to protocol. On this dose level, 4 patients were further treated (Figure 1). Patients 011 and 018 also developed DLT as isolated Troponin T hs elevation (grade 3; peak values UPN 011: 214 ng/L, UPN 018: 56.4 ng/L) without any clinical symptoms or sequelae, of whom patient 011 again had an elevated value before tTF-NGR. In both cases, values returned to the values before treatment within a week after tTF-NGR removal and anticoagulation with enoxaparin with or without ASS.

On the next de-escalated dose level of 3 mg/m^2^, isolated Troponin T hs elevation to values >50 ng/L occurred only in 1 patient (UPN 021, values already elevated to 27 ng/L before tTF-NGR application, peak value: 376 ng/L) with later decrease to values before treatment. Eight patients were treated at this dose level, 7 patients with 3.0 mg/m^2^ as the starting dose and 6 patients obtaining 2 cycles or experiencing DLT (Figure 1). One DLT (UPN 021, Troponin T hs) occurred at this dose with 12 cycles applicated. Thus, the MTD for tTF-NGR was determined as 3 mg/m^2^/day × 5, q day 22.

There were 3 completely reversible CTCAE grade 2 thromboembolic events under treatment with tTF-NGR (Figure 1 and Table 1). In the following, they are briefly described. The 1st event was a small deep vein thrombosis in patient UPN 008 (5 mg/m^2^) verified by sonography in the left lower limb at the same time the Troponin T hs elevation occurred. This AE was resolved clinically and in sonography already a few days later under enoxaparin (grade 2). Although the metastatic cancer patient was immobilized, the event was judged as related to tTF-NGR. The 2nd episode was observed in patient UPN 015 (4 mg/m^2^; medullary thyroid cancer, after surgery with partial neck lymph node dissection and systemic treatment). Cycle 1 was without AE. The patient had no central venous port system, and a subclavian catheter was placed for the 2nd cycle in the same position as for the 1st cycle. A catheter-associated subclavian thrombosis occurred at day 1 of the 2nd cycle. tTF-NGR was stopped, and the thrombosis was completely resolved within few days under anticoagulation with enoxaparin. Although catheter-associated thrombosis in cancer patients, here in a previously operated area in addition, is known, the event was judged as related to tTF-NGR (grade 2). This event prompted the investigators to increase the fluid volume for the tTF-NGR solution from 50 to 100 mL applied in 1 h, as allowed by protocol and discussed with the Data Safety Monitoring Board (DSMB). The 3rd episode was observed in a patient (UPN 019; 3 mg/m^2^) with a metastasized angiosarcoma of the heart after cardiac surgery, radiochemotherapy, and multiple chemotherapies, with a large tumor remaining in the wall of the left heart atrium. The first cycle was tolerated well. Approximately 3 h after the first application of tTF-NGR in cycle 2, the patient complained about dizziness and visual disturbances. The symptoms could not be substantiated by a neurological examination, but an immediately performed CNS angio-CT showed an embolic occlusion of the P3 of the left posterior cerebral artery. During the angio-CT, the patient completely and permanently lost the symptoms. Thus, besides stopping further tTF-NGR applications, no specific treatment was given. A control angio-CT on the next day showed no vascular occlusion and MRI ruled out any tissue damage by Diffusion Weighted Imaging. The event was judged as a transient ischemic attack (grade 2) and was related to tTF-NGR, although the DSMB argued in favor of peritumoral thrombotic material possibly present on the sarcoma lesion in the left atrial wall of the heart as an additional cause.

There was 1 death on trial due to a sepsis before tTF-NGR was given and was thus unrelated to tTF-NGR (UPN 016).

A MedDRA v.22.1 coded table for all Serious Adverse Events (SAE) and SUSAR possibly related to tTF-NGR with relation to dose level and UPN is given in Supplementary Table S3. The study protocol requested to control a broad variety of safety laboratory values including global coagulation parameters and cellular blood counts. Besides the events described above, we have observed minor increase of D-dimer and decrease of platelets, always reversible after the end of tTF-NGR application. This might represent activated coagulation and fibrinolysis but did not qualify for AE with few exceptions (Supplementary Table S1). Otherwise, tTF-NGR was well tolerated.

As can be seen in Figure 1, end of treatment (EOT) and thus also the number of cycles applied were defined by progressive tumor disease (PD) in 10 patients (UPNs 003, 004, 005, 006, 007, 012, 020, 022, 023, and 024), by DLT in 5 patients (UPNs 008, 009, 011, 018, and 021), and by thromboembolic event grade 2 in 2 patients (UPNs 015 and 019).

### 3.3. Antitumor Activity

Contrast-enhanced Ultrasound (CEUS): We could measure representative metastatic lesions and reference areas in the livers of 10 out of the 17 patients before and during tTF-NGR. The other patients had no lesions unequivocally measurable by CEUS. In all but one of these patients, we observed a major decrease of the tumor lesion´s blood flow. In contrast, the adjacent normal reference areas, e.g., in the liver, were either not affected or showed even increased perfusion after tTF-NGR. In the case (UPN 011) without tumor blood flow inhibition when compared with values before therapy, the reference liver tissue had approximately 6-fold increased blood flow under tTF-NGR, whereas the tumor lesion had none (Supplementary Table S4). However, the inhibition of tumor blood flow was not complete and varied within limits. Also, there is not a sufficient number of patients to delineate a clear dose response relation of this tumor blood flow inhibition. When categorized in a semiquantitative way, of the 3 patients treated with tTF-NGR doses <3mg/m^2^ and with measurable lesions (UPNs 003, 004, and 006; Supplementary Table S4), 2 had a maximum tumor blood flow inhibition of ≥90% and 1 had that of ≥50%. Of the 3 patients treated with >3 mg/m^2^ and measurable lesions (UPNs 011, 012, and 015), the maximum tumor blood flow decrease was <50% in 1, >50% in the 2nd, and >90% in the 3rd. Of the 4 patients treated at the MTD of 3 mg/m^2^ tTF-NGR dose level (UPNs 019, 020, 022, and 023), tumor blood flow decrease was <50% in 2 and >50% in the other 2 patients.

**Figure 2:**
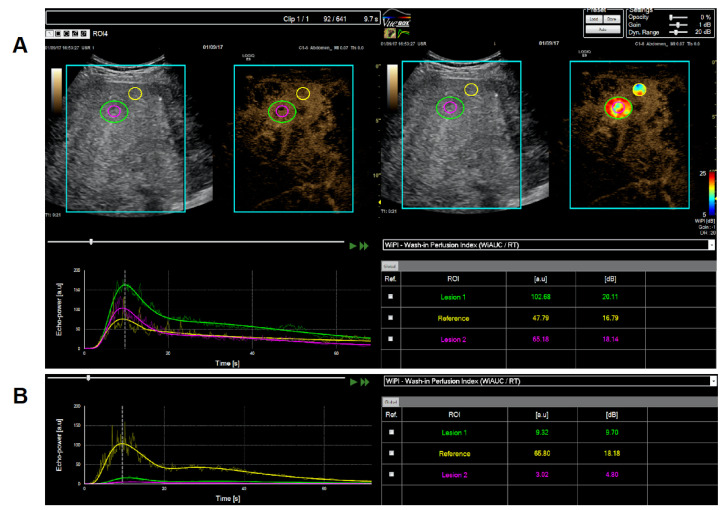
Contrast-enhanced ultrasound (CEUS) measurement (UPN 006) of wash-in perfusion index (WIAUC/RT, see Supplementary Figure S1) in liver metastasis (green and purple) versus normal liver (yellow) reference region of interest (ROI) before (upper panel, (**A**)) versus after 5 days of treatment with 2.5 mg/m^2^ tTF-NGR per day (lower panel, (**B**)).

**Figure 3:**
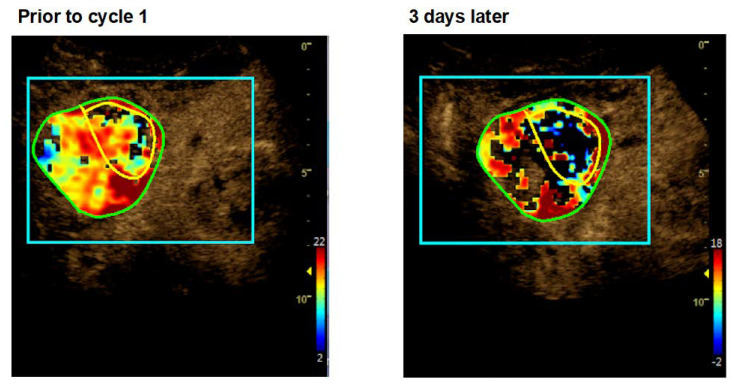
Heat maps of dynamic CEUS in a liver metastasis lesion of a colon adenocarcinoma (UPN 023) before and after 3 days of tTF-NGR (cycle 1). **Left part**: Before treatment with tTF-NGR at 3.0 mg/m^2^. **Right part**: Decrease of contrast perfusion after 3 days of 3 mg/m^2^ tTF-NGR. Color scales at the right lower corner of each photograph semiquantify contrast perfusion (red = high, blue/black = low). The green circle represents “total lesion”, and the yellow circle represents the “central lesion” (for values, see Supplementary Table S4).

As an example, patient UPN 006 treated with 2.5 mg/m^2^ tTF-NGR showed approximately a 1-log-step decrease of the tumor blood flow (Figure 2). A CEUS heatmap example of patient UPN 023 (cycle 1) before and after 3 days of 3 mg/m^2^ tTF-NGR is shown in Figure 3 (quantitative data of all 10 patients, Supplementary Figure S2 and Supplementary Table S4).

**Figure 4:**
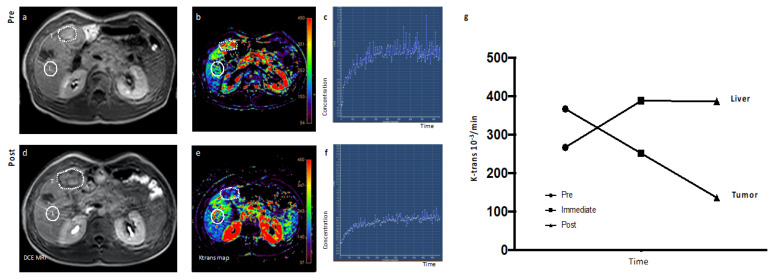
Early evaluation of activity of tTF-NGR using Dynamic Contrast-Enhanced Magnetic Resonance Imaging (DCE-MRI): Transverse images show the liver in a 22-year-old patient (UPN 003) with metastatic non-seminomatous germ cell tumor as treated in cycle 1 (1 mg/m^2^ tTF-NGR per day). Images of tumor (T) before (**a**–**c**) and after five days of therapy (**d**–**f**): Comparisons of tumor diameter before treatment (dotted line (**a**)) versus after treatment (dotted line (**d**)) reveal a moderate increase in tumor diameter 5 days after the beginning of treatment. Comparisons of tumor perfusion maps (volume transfer coefficient k-trans) demonstrate a notable reduction in tumor perfusion (**b**,**e**). Modelled perfusion parameter (k-trans) from before treatment (**c**) and after treatment (**f**) show a reduction in calculated k-trans values of >50% and delayed enhancement kinetics, indicating a substantial reduction in tumor perfusion. In contrast, healthy liver tissue (L in Figure 4a) shows a moderately enhanced perfusion after therapy. The line diagram (**g**) visualizes a first reduction of tumor perfusion already 5 h after treatment initiation (immediate) compared to the baseline scan (pre), with further decrease after five days of therapy (post).

Dynamic Contrast-enhanced (DCE)-Magnetic Resonance Imaging (MRI): We also measured tumor blood flow before and after tTF-NGR application using different dynamic and contrast-enhanced MRI methods (using Gadobutrol^®^ and Resovist^®^). At least one measurement was possible in 15 out of 17 treated patients for k-trans and in 14 out of 17 for vascular volume fraction (VVF; all values and effect sizes in Supplementary Figure S4). In all patients without DLT and completed cycle 1, we observed a major decrease of tumor blood flow, again without affection of blood flow in adjacent normal tissues (note that measurement of VVF is normalized on muscle tissue). As an example, Figure 4 shows the early tumor blood flow decrease (by k-trans measurement) in the hepatic metastasis of a patient with late-stage germ cell tumor in contrast to normal adjacent tissues (UPN 003; for tumor adjacent normal liver tissue, see L in Figure 4).

**Figure 5:**
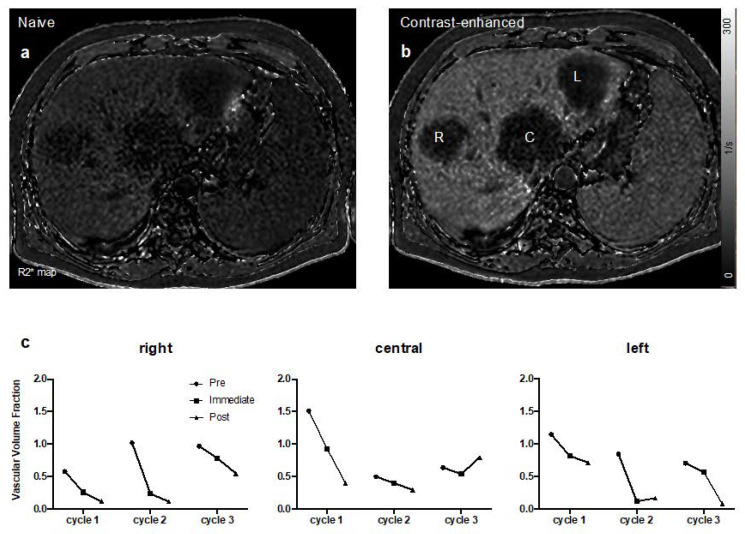
Early evaluation of tumor perfusion response to tTF-NGR using R2* mapping in MRI: R2* maps before (**a**) and after (**b**) injection of the iron-based contrast medium Resovist^®^ visualize the perfusion of 3 liver metastases (R = right, C = central, and L = left) of a patient with a colorectal carcinoma (UPN 007). The calculated vascular volume fractions (VVF) demonstrate a reduction in blood flow after treatment with tTF-NGR for each of the 3 cycles (**c**) at 3 (cycle 1), 4 (cycle 2), and 5 (cycle 3) mg/m^2^ tTF-NGR per day × 5. Notice the partial reperfusion at the beginning of cycles 2 and 3. (**c**) y-axis shows arbitrary units, black circles represent measurements before (pre) treatment with tTF-NGR, black squares are 5 h (immediate) after tTF-NGR, and black triangles are after 5 days (post) of tTF-NGR at the end of a cycle.

Figure 5 shows the VVF development over 3 cycles of therapy with increasing doses of tTF-NGR in patient UPN 007. Sequential measurements showed decrease of VVF of ≥1-log step after each cycle of tTF-NGR when compared to values before treatment onset with partial recovery in some lesions before start of the next cycle. As effects were already submaximal with 3 mg/m^2^ × 5, no further dose-activity relation could be observed in this patient. Quantitative values for all patients are given in Supplementary Table S5.

CD13 in histology: All patients except two (UPN 020, no material left; UPN 022 hepatocellular carcinoma, diagnosis by imaging and tumor marker) expressed the binding target CD13 on the tumor vasculature, the tumor cells, or both (Supplementary Table S6). Due to the material available, we could not perform a systematic study on CD13 expression of normal tissue adjacent to the tumor. Interestingly, we have observed major tumor blood flow reductions in MRI as measured by k-trans and VVF also in patients with CD13 positivity (intensity scores 2; for methodology see ref. 20) of the tumor vasculature only, with the tumor cells being CD13-negative (Supplementary Figure S3).

In conclusion, we have observed proof of principle for the mode of action of tTF-NGR by showing strong and selective tumor blood flow inhibition by the IMP with two independent methods in all patients with measurable lesions.

RECIST: This phase I trial enrolled very late-stage cancer patients with high tumor load, multiple previous therapies, and an estimated minimum life expectancy of 6 weeks. Early tumor progression with deterioration of performance was the reason for the majority of screening failures by the decision of the investigator, and 1 death occurred before tTF-NGR could be given. In none of the 17 patients treated, a response (CR and PR) could be observed. All patients showed progressive disease (PD) after two cycles, with the exception of one patient being progressive after one and of one after the third cycle (Figure 1). The times to PD were comparable to disease kinetics before study. All patients eventually died from progressive disease (PD). Two patients (UPNs 020 and 024) reported a notable improvement of quality of life and mobility after cycle 1.

**Figure 6:**
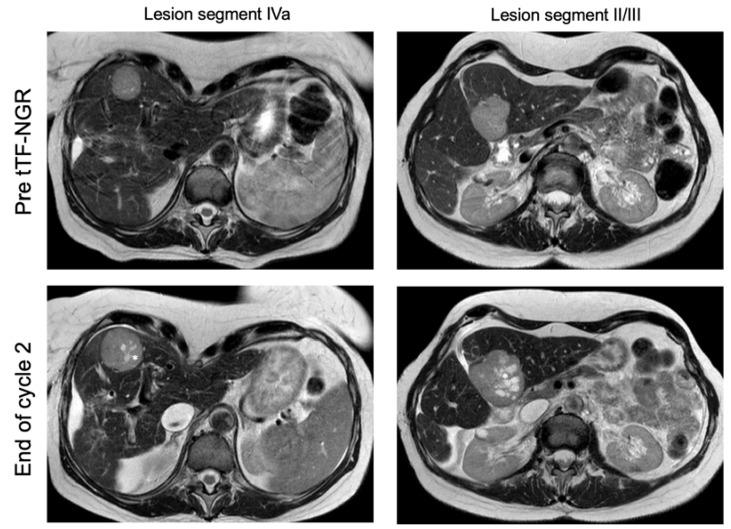
Morphological changes in hepatocellular carcinoma (HCC) (UPN 022) under tTF-NGR therapy: In the initial MRI scans, two prominent HCC lesions are visible in a transversal T2 weighted image. Before treatment with tTF-NGR, the HCC lesions are homogenously hyperintense as compared to the surrounding liver tissue (**upper panels**). After two cycles with tTF-NGR at 3.0 mg/m^2^ × 5, morphological changes with multiple hemorrhagic regions (*) have occurred inside the tumor lesions (**lower panels**), and at the same time, enlargement of tumor diameters can be seen.

As evidence of antitumor activity of tTF-NGR, we have observed intratumoral areas in some tumor lesions in the tumor imaging compatible with necrosis and/or hemorrhage occurring after tTF-NGR application, leading to swelling of the tumors. In DCE-MRI, this was visible with morphological changes inside the lesion showing T2w hyperintense areas when compared with surrounding liver tissue (examples are shown in Figure 6 and Supplementary Figure S5), which is interpreted as hemorrhagic or necrotic areas. Since we have observed this phenomenon as a mechanism of action also in the mouse xenograft studies showing tumor swelling within the first days after treatment onset and intratumoral hemorrhage and necrosis in histological sections [14], this could lead to misinterpretation as early disease progression as is also described for immune checkpoint inhibitors [22]. Table 1 gives the patients in whom such an observation was made either in CEUS or in DCE-MRI.

### 3.4. Pharmacokinetics (PK)

**Figure 7:**
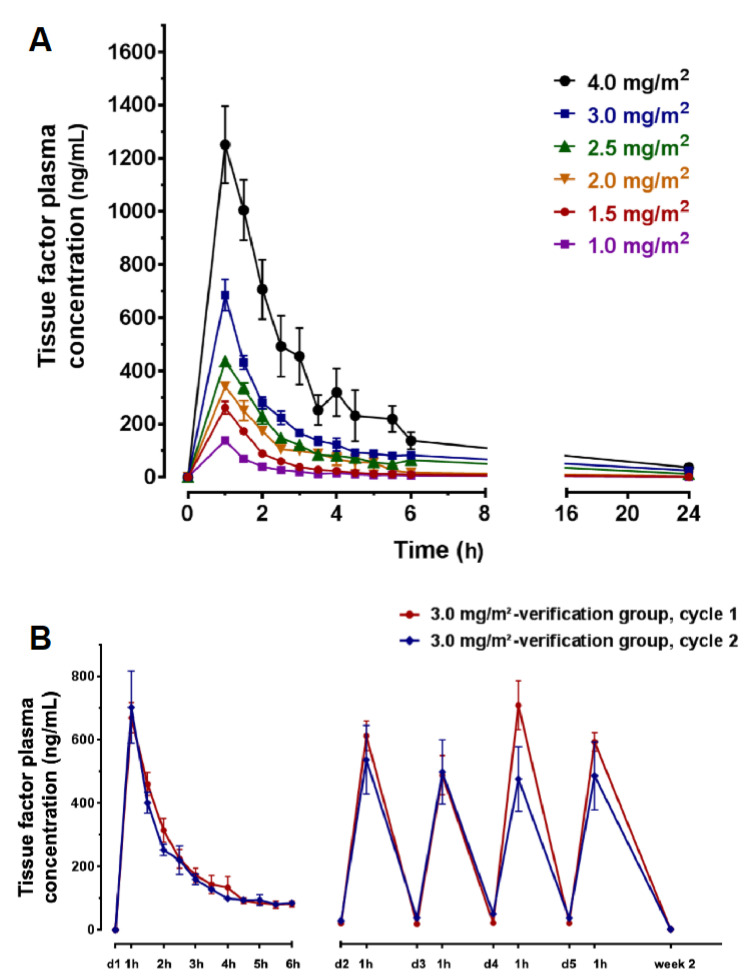
Pharmacokinetics with plasma concentration—time curves of tTF-NGR for different doses applied: (**A**) Cmax values at the end of infusion (1 h) and dose-time curves when 1–4 mg/m^2^ tTF-NGR were given over 1 h on day 1 (0–24 h). Interpretation of pharmacokinetic (PK) values at 5 mg/m^2^ dose level (see Supplementary Table S7) was complicated by difficulties to draw and prepare blood samples for testing due to sticky consistency. (**B**) Values of all patients in the verification cohort at 3 mg/m^2^ tTF-NGR at day 1, at each of the following 4 days of tTF-NGR application (peak at the end of infusion and trough before the next application), and for cycle 1 (red) and cycle 2 (blue).

We determined PK results of tTF-NGR in human plasma from all 17 patients treated (details in Supplementary Table S7). The C_max_ levels and AUCs revealed a dose-related exposure of the patients to tTF-NGR during the treatment period. Mean peak plasma levels of tTF-NGR in patients treated with 2.0, 3.0, or 4.0 mg tTF-NGR/m^2^ body surface area/day by a 1-h intravenous infusion for 5 days were generally observed 1 h after start of dosing with C_max_ values of 453.66, 788.74, and 1205.20 ng/mL, respectively (Figure 7A). The individual plasma concentration time curves for all patients (except UPN 024, who had no sampling exactly at the end of infusion) at tTF-NGR dose level 3.0 mg/m^2^ are given in Figure 7B. The calculated mean alpha plasma distribution half-life of tTF-NGR at 3 mg/m^2^ was 1.14 h. tTF-NGR was eliminated with a mean terminal half-life of 8.99 h. No significant differences in peak plasma levels (C_max_) of tTF-NGR at a given dose-level among multiple-dose administration (×5) were found (Figure 7B). The mean concentration of tTF-NGR was 30.1 ng/mL at 24 h post-administration. The dose proportionality factor (DPF) resulted in values >1.0 (Supplementary Table S7 and Supplementary Figure S6), indicating an elimination lower than proportional with higher doses. This is in contrast to the behavior in dogs, where we observed a DPF close to 1.0 (data in IB on file). However, comparing plasma levels of tTF-NGR before first treatment with those short before subsequent treatments in patients with treatment repeating identical doses over several cycles has not shown major plasma accumulation over a period of 5 daily applications (Figure 7B). The same holds true when cycle 1 was compared with subsequent cycles (Supplementary Table S7). Since the next administration of tTF-NGR, when given daily, follows intervals of approximately 2.5 to 3 elimination half-lives (t_1/2(terminal)_ ~8 to 9 h), accumulation of the drug in the body is not to be expected with the schedule applied. Interpretation of PK values at the 5 mg/m^2^ dose level was complicated by difficulties to draw and prepare blood samples for testing due to sticky consistency of the blood samples.

### 3.5. Human Anti-Fusion Protein (tTF-NGR) Antibodies (HAFA)

Fifteen out of the 17 patients treated did not develop human anti-tTF-NGR antibodies over 1 to 2 cycles. As shown in Supplementary Figure S7, 2 patients (UPN 003 and UPN 007) developed low titers of anti-tTF-NGR IgG. Interestingly, we observed this in patient UPN 007 (Supplementary Figure S7C) treated with 3 cycles, but this patient showed drastic reduction of tumor blood flow also during the 3rd cycle (see Figure 5) with low IgG titers present. Thus, we conclude that IgG antibody formation was not interfering with the MOA of tTF-NGR. We did not observe any signs of clinical reaction to HAFAs in these patients. Further, in UPN 007, the HAFA did not compromise measured AUC increase per dose applied (Supplementary Table S7); thus, the HAFA binding site must be different from the one of the detection antibody in the PK analysis. Binding position within the molecule has to be characterized in future studies, with N-terminal histidine tag being a candidate.

### 3.6. Single-Parameter Correlations

Despite the small number of patients, the different tumor entities, and the different dose levels of tTF-NGR applied, we have with due caution tested for some correlations in a hypothesis-generating approach. No significant correlations were found a) correlating CD13 expression (tumor cells, vascular cells, composite stain (Supplementary Table S6)) with effect size (best change from baseline in cycle 1 in CEUS (Supplementary Table S4 and Supplementary Figure S2) or DCE-MRI (Supplementary Table S5 and Supplementary Figure S4; Kruskal–Wallis test); b) comparing effect size (best change from baseline in cycle 1) with the single methods CEUS with DCE-MRI and comparing DCE-MRI k-trans with VVF (Spearman correlation coefficient); c) correlating Troponin T hs after therapy with effect size (best from baseline in cycle 1) in DCE-MRI; and d) correlating survival time and dose level (Cox regression). A correlation trend was observed for Troponin T hs after therapy with age of the patients (Kruskall–Wallis test, *p* = 0.142). Trends or significant correlations were observed for effect size (best change from baseline in cycle 1) with IMP dose applied (Spearman correlation coefficient). CEUS to absolute dose applied (per cycle 1; Supplementary Tables S2 and S4) showed a trend (correlation coefficient r = 0.498, *p* = 0.142), and DCE-MRI VVF measurements showed a significant correlation with absolute dose applied per cycle 1 (r = 0.556, *p* = 0.038) but not with dose level per m^2^, whereas k-trans measurements only showed trends. A lower Troponin T hs after therapy was correlated with higher effect size (best change from baseline in cycle 1) for DCE-MRI VVF (Kruskal–Wallis test, *p* = 0.0267).

## 4. Discussion

This is the first phase I clinical trial with the first-in-class CD13-targeted tissue factor tTF-NGR. With 17 patients treated, we were able to characterize the DLT as an isolated elevation of Troponin T hs, which was reversible upon discontinuation of tTF-NGR and by anticoagulation and without clinical sequelae or other connected signs of toxicity. Furthermore, 3 episodes of thromboembolism were observed, all grade 2 (CTCAE) and reversible on anticoagulation, which were classified as drug-related, although other risk factors could have contributed as described. Otherwise, tTF-NGR was well tolerated, and the MTD for this schedule was determined as 3 mg/m^2^/day × 5, q day 22.

The exact meaning of the Troponin T hs elevation is complex. CTCAE 4.0 does not specify high-sensitivity cardiac troponins but only cardiac Troponin T and I. Cardiac troponins are regulators of actin and myosin and are released into plasma upon myocardial damage and in acute coronary syndrome. High-sensitivity troponin assays can reliably measure small increases in plasma still undetectable with the other assays and well before clinically detectable cardiac damage. Multiple anticancer drugs of different classes, such as anthracyclines, trastuzumab, sunitinib, and nivolumab, can cause troponin increase, being sometimes prognostic for later clinical cardiac dysfunction [23,24,25,26,27]. This was not generally described as DLT, but given the mode of action of tTF-NGR, we considered it to be cautious and justified to define Troponin T hs elevation as such. However, troponin increases are also described in other conditions resulting in secondary cardiac wall stress such as sepsis and pulmonary embolism or with yet unclear relation to myocardium such as stroke or gastrointestinal bleeding [28,29,30,31]. In our patients, there was no relevant renal dysfunction to complicate interpretation and troponin T hs increase was always reversible upon discontinuation of drug application and anticoagulation. Due to the low number of patients, it was impossible to detect risk factors for Troponin T hs elevation under treatment; however, there was a trend in association with higher age. We have retrospectively assayed the beagle plasma samples in the animal safety studies and could not observe elevated Troponin T hs even at the highest dose of tTF-NGR applied (20 mg/m^2^/day × 5). We interpret this elevation as a possible surrogate for hypoxic cell stress and cell death within myocardial tissue and thus as a sensitive early sign, valuable for prevention of clinically relevant and symptomatic side effects. Patient UPN 024 had a preexisting elevated Troponin T hs due to an inflammatory reaction, which could be lowered and kept within normal range under tTF-NGR by corticosteroids. Thus, patients with preexisting Troponin T hs of >50 ng/L and/or history of coronary heart disease or any type of previous cerebral ischemia should not be included in future trials with tTF-NGR. Further, frequent controls of Troponin T hs and stop of treatment upon increase of values to >50 ng/mL are advisable, but later re-institution at lower doses can be envisaged.

We have observed proof of principle for the mode of action of tTF-NGR with major and selective decrease in tumor blood flow not affecting normal tissue with two independent dynamic imaging methods in the measurable lesions of all patients. For DCE-MRI measurement of VVF, there was a significant dose–activity relation. The reduction of tumor blood flow by tTF-NGR was not restricted to a specific tumor histology, supporting the observation that the tumor vasculature is the primary target of tTF-NGR and not the tumor cells. The duration of tumor blood flow inhibition varied between patients and lesions, and partial reperfusion was sometimes observed at the beginning of the next cycle. In parallel, limited drops of platelet numbers and elevations of D-dimer have been observed, indicating some coagulatory activity. In the 17 patients treated, no objective response was seen according to RECIST. In preclinical experiments, tTF-NGR mainly induced tumor growth inhibition and rarely induced tumor remissions [14,15]. In some tumor lesions, swelling by intratumoral hemorrhage or necrosis could be seen as evidence for activity. Thus, it might be advisable for future studies with tTF-NGR to use one of the immunological modifications of RECIST to avoid misinterpretation of pseudoprogression as progressive disease whenever time to progression or progression-free survival is a primary endpoint [22].

As can be envisaged today, inclusion criteria for future trials with tTF-NGR are not necessarily restricted by tumor histology but by CD13 target presence and exclusion criteria besides a preexisting elevated Troponin T hs are certainly any kind of increased thromboembolic risk deduced from case history or risk category as used for cancer patients.

There are two hypothetical advantages of performing phase I trials in this setting with intraindividual dose-escalation testing IMPs with PK-characteristics such as tTF-NGR. First, fewer patients are needed to reach MTD than using the traditional Fibbonacci 3 + 3 design. This was not the case in this trial, as Fibonacci dose escalation would have led to MTD with ≥18 patients and this trial needed 17. Second, more patients are treated in the upper and potentially active dose levels of the IMP. As 14 out of 17 patients were treated with dose levels ≥ 3 mg/m^2^/day × 5, q day 22, this was the case. Most important, intraindividual dose escalation was safe for the patients treated in this trial, as no life-threatening toxicity was observed.

The IMP, tTF-NGR (His-tag-tTF_1-218_-GNGRAHA), is the lead compound of our series of fusion proteins carrying tumor-targeting peptides instead of the transmembrane domain at the C-terminus of truncated tissue factor for induction of tumor infarction [13,14,15,16,17,18,19,20]. Interestingly, sequential application of doxorubicin with subsequent tumor vascular occlusion by tTF-NGR at maximum intratumoral doxorubicin levels yields favorable combinatorial therapeutic results in vivo in a variety of tumor entities [19]. The main binding site of tTF-NGR is aminopeptidase N (APN, also known as CD13) [6], a transmembrane enzyme expressed in a variety of tissues and cells with different functions including degradation of extracellular material for an invasive cell [5,6,7,8]. It is upregulated on endothelial cells and pericytes in tumors and tissues that undergo angiogenesis and is operational in capillary tube formation [6,32,33]. CD13 protein is also present in some normal tissues [34], but expression outside blood vessels is of minor importance for the mode of action of tTF-NGR, since for pro-coagulatory activity, this molecule needs a coagulation-competent area. However, areas of vascular remodeling, such as in wound healing, in ischemic myocardium, in small vessels as present in CNS [34], or in the uterine cycle, are of general concern and should be considered before exposing patients to tTF-NGR. CD13 is often used for experimental tumor imaging [35,36,37,38]. The preclinical antitumor activity of tTF-NGR in vivo is not restricted by tumor histology, as the main target is the tumor vasculature. Even in tumor entities such as small cell lung cancer xenografts, in which the tumor cells rarely express CD13 whereas vascular cells stain positive, significant therapeutic effects of systemically administered tTF-NGR can be shown [39]. In this respect, it is interesting that we have observed major tumor blood flow reductions in DCE-MRI also in patients with CD13 positivity in cells of the tumor vasculature only, with the tumor cells being CD13 negative.

Besides tTF-NGR, another NGR-peptide-targeted molecule is in clinical trials in oncology. NGR-hTNF is studied in phases II/III [40,41,42]. In addition to NGR peptides, CD13 is targeted by a variety of different compounds [43] and bestatin has been reported to increase the overall survival of patients with early-stage squamous cell lung cancer when given after surgery in a randomized trial [44]. Phase II/III trials with tTF-NGR are in preparation. However, tissue factor biology in cancer is complex [45], and the endogenous molecule plays a role in tumor progression and cancer-associated thrombosis and can influence antitumor immunity [46,47,48]. Thus, further studies with tTF-NGR must be performed with caution and in a clinical setting with specific hemostaseology expertise available.

## 5. Conclusions

This is the first clinical study with a pro-coagulatory protein fused to a peptide targeting the tumor vasculature for inducing tumor infarction. The study shows that the fusion protein tTF-NGR can be safely applied to patients with advanced cancer with good tolerability and that its dose-limiting toxicity is manageable. In the majority of the patients, major inhibition of tumor blood flow in contrast to normal tissue blood flow could be shown by two dynamic imaging methods (CEUS and DCE-MRI) as proof of principle. Together with the extensive preclinical testing of the fusion protein tTF-NGR, this study provides strong rationale for continued exploration of this molecule in phase II/III trials in patients suffering from advanced malignant disease. On the basis of targeting the tumor vasculature, potential application is independent from tumor histology.

## Figures and Tables

**Table 1:** Characteristics of the patients treated with truncated tissue factor (tTF) with a C-terminal NGR peptide (tTF-NGR).

UPN	Gender (m/f)	Age (year)	Diagnosis	Previous Tx	tTF-NGRcycles	tTF-NGR Dose (mg/m^2^)	DLT Related to IMP	Imaging (I), Outcome (O), and QOL (Q)
003	m	22	metastatic non-seminomatous germ cell tumor	PEI, HD-PEI, surgery, TI, TIP, HD-CE, Gem/Ox, Radiation, Nivolumab	2	cycle 1: 1.0 cycle 2: 1.5	good tolerability, no DLT	I: hemorrhagic/necrotic areas in liver lesions upon therapy (MRI) O: RECIST PD; Q: n.c.
004	f	29	hepatocellular carcinoma + malignant ascites, multifocal	surgery, Sorafenib, Regorafenib, TACE	2	cycle 1: 1.5 cycle 2: 2.0	good tolerability, no DLT	I: hypointense areas in liver lesions upon therapy (sonography) O: RECIST PD; Q: n.c.
005	m	63	small cell lung cancer (SCLC), extensive disease, metastatic	radiochemo CE, CNS-PCI, CE, Topotecan, ACO	2	cycle 1: 2.0 cycle 2: 2.5	good tolerability, no DLT	O: FUO unrelated to IMP; RECIST PD Q: n.c.
006	m	46	rectal adeno, metastatic	neoadj. radiochemo, surgery + HIPEC, FOLFOX, liver surgery, radiation, FOLFIRI + Bev, SIRT, FOLFOX + Bev, TAS-102 (Trifluridin/Tipiracil)	2	cycle 1: 2.5 cycle 2: 3.0	good tolerability, no DLT	O: RECIST PD Q: n.c.
007	m	58	colon adeno, metastaticRAS-mutated	surgery, FOLFOX + Bev, FOLFIRI + Bev,Ramucirumab,TAS-102	3	cycle 1: 3.0 cycle 2: 4.0 cycle 3: 5.0	good tolerability, no DLT	O: RECIST PD Q: n.c.
008	m	45	synovial sarcoma, metastatic	surgery, Doxo/Ifo, Trabectedin, Gem/Doc, Doxo + Fibromun (study), Pazopanib, Bendamustin, Vandetanib, HD-Ifo	1	Cycle 1: 5.0 (3 days)	DLT, related to IMP, Troponin T hs, grade 3; DVT, related to IMP, grade 2	O: DLT (Trop) and DVT reversible; RECIST PD Q: n.c.
009	m	56	lung squamous, metastatic	radiochemo, Cisplatin/Vinorelbin, Nivolumab, Docetaxel, Gemcitabine, Afatinib,	1	cycle 1: 5.0 (1 day)	DLT, related to IMP, Troponin T hs, grade 3	O: DLT (Trop) reversible Q: n.c.
011	m	74	colon adeno, metastatic	surgery, FOLFOX + Bev, FOLF + Bev + anti-PDL-1 (study), FOLFIRI + Cetuximab, FOLFOX + Cetuximab	1	cycle 1: 4.0 (2 days)	DLT, related to IMP,Troponin T hs, grade 3	O: DLT (Trop) reversible; RECIST PD Q: n.c.
012	m	26	extragonadal germ cell tumor, metastatic	PEI, surgery, TI, TIP, HD-CE, radiation, Gem/Ox/Paclitaxel	1	cycle 1: 4.0	good tolerability, no DLT	O: only 1 cycle since fast PD Q: n.c.
015	m	63	medullary thyroid cancer	surgery, Vandetanib, Cabozantinib	1(com-pleted)	cycle 1: 4.0 cycle 2: 4.0 (1 day)	cycle 1: good tolerability, no DLT; cycle 2: catheter-associated DVT related to IMP, grade 2	O: catheter-associated DVT reversible; RECIST PD Q: decreased in cycle 2, reversible
018	f	54	undifferentiated teratoma, then squamous carcinoma, CUP, metastatic	surgery, PEB, PEI, Carboplatin/Paclitaxel	1	cycle 1: 4.0 (3 days)	DLT, related to IMP, Troponin T hs, grade 3	O: DLT (Trop) reversible; RECIST PD Q: n.c.
019	f	41	angiosarcoma of the heart, metastatic	surgery, radiochemo, Paclitaxel, Doxo/Ifo, Pazopanib, radiation, Gem/Doc, Gem, Trabectedin, SIRT	1	cycle 1: 3.0 cycle 2: 3.0 (1 day)	cycle 1: good tolerability; cycle 2: TIA, related to IMP, grade 2	O: TIA reversible; RECIST PD Q: n.c.
020	m	19	desmoplastic small/round cell tumor, metastatic	VIDE, VAI, Temodal/Irinitecan,	2	cycle 1: 3.0 cycle 2: 3.0	good tolerability, no DLT	I: hypointense areas in liver lesions upon therapy (sonography) O: RECIST PD Q: improved under therapy
021	m	63	synovial sarcoma, metastatic	surgery, radiation, Doxo/Ifo, Trabectedin, Gem/Doc,	1	cycle 1: 3.0 (1 day)	DLT, related to IMP, Troponin T hs, grade 3	O: DLT (Trop) reversible; RECIST PD Q: n.c.
022	f	56	hepatocellular carcinoma, metastatic	TACE, SIRT, Sorafenib (toxicity), Nivolumab (toxicity)	2	cycle 1: 3.0 cycle 2: 3.0	good tolerability	I: hemorrhagic/necrotic areas in liver lesions upon therapy (MRI) O: RECIST PD; Q: n.c.
023	m	60	colon adeno, metastatic	FOLFIRI-OX + Panitumumab, 5-FU + Panitumumab, radiotherapy, FOLFIRI + Bevacizumab, Trifluridin/Tipiracil, Irinotecan + Cetuximab	2	cycle 1: 3.0 cycle 2: 3.0	good tolerability	O: RECIST PD Q: n.c.
024	m	24	undifferentiated pleomorphic sarcoma, metastatic	EURAMOS protocol (Cisplatin, Doxo, HD-MTX and surgery), adjuvant Mifamurtid, Ifo + Etoposide, surgery of metastasis, radiotherapy, Gem/Doc, Nivolumab, Pazopanib		cycle 1: 3.0 cycle 2: 3.0	good tolerability	O: RECIST PD Q: improved under therapy

UPN numbers not mentioned belong to patients with screening failure and/or not treated with tTF-NGR; PEI; Cisplatin, Etoposide, Ifosfamide (Ifo); HD-PEI, high-dose PEI with stem cell rescue; TI, Paclitaxel, Ifo; TIP, TI plus Cisplatin; HD-CE, HD Carboplatin, Etoposide; Gem/Ox, Gemcitabine, Oxaliplatin; TACE, transarterial chemoembolization; CNS-PCI, prophylactical brain irradiation; ACO, Doxorubicin, Cyclophosphamide, Vincristine; HIPEC, hyperthermic intraperitoneal chemotherapy; FOLFOX, 5-Fluorouracil, folinic acid, Oxaliplatin; Bev, Bevacizumab; FOLFIRI, 5-Fluorouracil, folinic acid, Irinotecan; SIRT, selective internal radiotherapy; Doxo/Ifo, Doxorubicin, Ifosfamide; Gem/Doc, Gemcitabine, Docetaxel; VIDE, Vincristine, Ifo, Doxo, Etoposide; VAI, Vincristine, Actinomycin, Ifo; FUO, fever of unknown origin; DVT, deep vein thrombosis; TIA, transient ischemic attack; DLT, dose limiting toxicity; IMP, investigational medicinal product; RECIST PD, progressive according to RECIST criteria; QOL, quality of life; n.c., no change.

## Supplementary Materials

The following are available online at https://www.mdpi.com/2072-6694/12/6/1488/s1, Supplementary Information File: Figure S1: Dynamic contrast-enhanced ultrasound (CEUS), Figure S2: CEUS blood flow decrease in all patients with measurable lesions, Figure S3: Reduction of tumor perfusion by tTF-NGR in a synovial sarcoma with CD13 positivity in the tumor vasculature only, Figure S4: Changes in tumor perfusion during treatment with tTF-NGR in all patients serially imaged, Figure S5: Intratumoral necrosis in liver metastases of a germ cell tumor (UPN 003), Figure S6: Visualization of the dose proportionality factor (DPF), Figure S7: Detection of human anti-fusion protein (tTF-NGR) IgG antibodies (HAFA) in the patients plasma, Table S1: AE listing, Table S2: Absolute doses of tTF-NGR given per patients, Table S3: Frequency of SUSAR and SAE possibly related to tTF-NGR, Table S4: tTF-NGR phase I study—CEUS maximum and minimum A.U. values for wash-in perfusion index in all patients with measurable lesions, Table S5: VVF and k-trans in magnetic resonance imaging under tTF-NGR application, Table S6: CD13 immunohistochemistry (IHC) staining intensity per patient and distribution according to histology, Table S7: Non-compartment analysis of tTF-NGR with various intravenous doses of tTF-NGR administered to cancer patients, Supplementary Material: v10 Study Protocol.
