# Supplementary material for: First-In-Class CD13-Targeted Tissue Factor tTF-NGR in Patients with Recurrent or Refractory Malignant Tumors: Results of a Phase I Dose-Escalation Study
Source: Cancers (Basel). 2020 Jun 7;12(6):1488. doi: 10.3390/cancers12061488 (PMC7352358; doi:10.3390/cancers12061488)
Supplement: Supplementary file 1 [file cancers-12-01488-s001.zip › cancers-841723_Protocol_final.pdf]

Title: **PHASE I STUDY OF tTF-NGR IN PATIENTS WITH RECURRENT OR REFRACTORY MALIGNANT TUMORS AND LYMPHOMAS BEYOND ALL STANDARD TREATMENTS**

Short title: **tTF-NGR Phase I Study**

EudraCT-No.: **2016-003042-85**

Sponsor:

University Hospital Muenster, Germany
Representative: Dr. Ch. Hoppenheit, Commercial Director
Albert-Schweitzer-Campus 1, Gebäude D5
D-48149 Münster, Germany
Telephone: +49 251 83 55802
Fax: +49 251 83 55803

Sponsors Study Code: UKM12_0018

Investigator:

PD Dr. Christoph Schliemann
Department of Medicine A (Hematology, Hemostaseology, Oncology)
University Hospital Muenster, Germany
Albert-Schweitzer-Campus 1
D-48149 Muenster, Germany
Telephone: +49 251 83 47587
Fax: +49 251 83 47588
E-mail: Christoph.Schliemann@ukmuenster.de

Deputy of the Investigator (Stellvertretender Prüfer):

Prof. Dr. Rolf Mesters
University Hospital Muenster, Germany
Department of Medicine A (Hematology, Hemostaseology, Oncology)
Albert-Schweitzer-Campus 1
D-48149 Muenster, Germany
Telephone: +49 251 83 47594
Fax: +49 251 83 48745
E-mail: Rolf.Mesters@ukmuenster.de

Protocol version 10: 2016 07 28

Study Contact: PD Dr. Christoph Schliemann
E-mail: Christoph.Schliemann@ukmuenster.de
Hotline: +49 251 8347587

Confidential

The information in this study protocol is strictly confidential. It may be used for the conduct of the study. It must not be available to persons or institutions who are not concerned with the study. Usage for other purposes requires written approval by the investigator.

Cancers **2020**, *12*, x; doi: www.mdpi.com/journal/cancers

Head of the Production Unit (GMP)
Dr. rer. nat. Christian Schwöppe
University Hospital Muenster, Germany
Department of Medicine A (Hematology, Hemostaseology, Oncology)
Albert-Schweitzer-Campus 1
D-48149 Muenster, Germany
Telephone: +49 251 83 56225
Fax: +49 251 83 56709
E-mail: christian.schwoeppe@uni-muenster.de

Statistician
Dr. rer. nat. Joachim Gerß, Dipl.-Stat.
Institute of Biostatistics and Clinical Research
Director: Prof. Dr. Andreas Faldum
University of Muenster
Schmeddingstr. 56
D-48149 Muenster, Germany
Telephone: +49 251 83 50660
Fax: +49 251 83 55277
E-mail: joachim.gerss@ukmuenster.de

Study Radiologist
Prof. Dr. Walter Heindel
University Hospital Muenster, Germany
Department of Radiology
Albert-Schweitzer-Campus 1
D-48149 Muenster, Germany
Telephone: +49 251 83 45139
Fax: +49 251 83 47312
E-mail: heindel@uni-muenster.de

Monitoring
Stefanie Dickmänken
Zentrum für Klinische Studien Münster
Universitätsklinikum Münster
Von-Esmarch-Str. 62
D-48149 Muenster, Germany
Tel.: +49 251 83 57256
Fax: +49 251 83 57026
E-mail: Stefanie.Dickmaenken@ukmuenster.de

SAE Management
Dr. Trude Butterfaß-Bahloul
Zentrum für Klinische Studien Münster
Universitätsklinikum Münster
Von-Esmarch-Str. 62
D-48149 Muenster, Germany
Tel.: +49 251 83 57109
Fax: +49 251 83 57112
E-mail: butterft@ukmuenster.de; mssd@ukmuenster.de

Data Management
Sonja Baier
Zentrum für Klinische Studien Münster
Universitätsklinikum Münster
Von-Esmarch-Str. 62
D-48149 Muenster, Germany
Tel.: +49 251 83 57496
Fax: +49 251 83 57026

Cancers **2020**, *12*, x; doi: www.mdpi.com/journal/cancers

E-mail: sonja.baier@ukmuenster.de

Signatures

**Sponsor:**	
University Hospital Muenster<br>represented by	
	______________________
Dr. Christoph Hoppenheit<br>(Commercial Director)	**Place, date**
	______________________
	**Signature**
**Statistician:**	
Institute of Biostatistics and Clinical Research	
Dr. Joachim Gerß	______________________
	**Place, date**
	______________________
	**Signature**
**Investigator:**	
Department of Medicine A (Hematology, Hemostaseology, Oncology)	
	______________________
PD Dr. Christoph Schliemann	**Place, date**
	______________________
	**Signature**

Cancers **2020**, *12*, x; doi: www.mdpi.com/journal/cancers

Table of contents

1.	General Information	1
2.	Protocol synopsis	5
3.	Protocol overview	9
4.	Background and rationale	13
5.	Study design	13
6.	Eligibility criteria and patient registration	14
7.	Treatment plan	16
8.	Dosing delays and dose de-escalation in individual patients	18
9.	Dose-Limiting toxicity (DLT) and maximum tolerated dose (MTD)	18
10.	Dose escalation and de-escalation according to tocixity and dose recommended for phase II trials (RDPII)	20
11.	Study objectives and end-points	23
12.	Patient evaluation and follow-up	24
13.	Reporting requirements	26
14.	Anticipated risks for the subjects	32
15.	Assessment of disease status	35
16.	Statistical considerations	35
17.	Study duration, premature termination of the study	36
18.	Informed consent	37
19.	Study administration	39
20.	Patient insurance	43
21.	Financing of the study	43
22.	Adherence to the protocol	43
23.	Data protection	44
24.	Reporting and publication policy	44

Appendix: Rekonstitution des Fertigarzneimittels tTF-NGR

Cancers **2020**, *12*, x; doi: www.mdpi.com/journal/cancers

2. Protocol Synopsis

**Title:**	PHASE I STUDY OF tTF-NGR IN PATIENTS WITH RECURRENT OR REFRACTORY MALIGNANT TUMORS OR LYMPHOMAS BEYOND ALL STANDARD TREATMENTS
**Short Title:**	tTF-NGR Phase I Study
**Sponsor's Study Code:**	UKM12_0018
**EudraCT-No.:**	**2016-003042-85**
**Sponsor:**	Universitätsklinikum Münster
**Investigator:**	PD Dr. Christoph Schliemann<br>Department of Medicine A (Hematology, Hemostaseology, Oncology), UKM<br>Albert-Schweitzer-Campus 1<br>D-48149 Muenster, Germany<br>Telephone: +49 251 8347587<br>Fax: +49 251 8347588<br>E-mail: Christoph.Schliemann@ukmuenster.de
**Indication:**	All solid tumors and lymphomas beyond standard therapy
**Study Design:**	Phase I Study
**Investigational Medicinal Product:**	tTF-NGR
**Therapy:**	Phase I, open label, single arm, non-randomized prospective, monocenter study. tTF-NGR will be given as 1-hour infusion via central venous access once daily for 5 days with a subsequent rest period of 2 weeks and following cycles with dose escalation of 0.5 mg/m^2 upon judgement of tolerability and therapeutic activity. Starting dose will be 1 mg/m^2/day. Patients within the dose-escalation part will be treated in sequence and not in parallel. Individual patients can undergo a maximum of 8 dose-escalations. Dose-escalation is stopped before the maximum number of 8 escalation steps if tumor response, tumor progression or a Dose-Limiting Toxicity (DLT) is observed. In the case of stable disease (SD) and with good tolerability these patients can obtain further cycles without dose-escalation until tumor progression or Dose-Limiting Toxicity (DLT) and the next patient can start with dose-escalation cycles on the highest tolerable dose for the previous patient.
**Objectives:**	Primary Objective:<br>To evaluate the maximum tolerated dose (MTD) and the Dose-Limiting Toxicity (DLT) of intravenously (iv) infused daily applications of tTF-NGR for 5 days every 3 weeks in patients with relapsed or refractory cancer, who had obtained all

Cancers **2020**, *12*, x; doi: www.mdpi.com/journal/cancers

	standard treatment known for their disease entity prior to entry on study.<br><br>Secondary Objectives:<br><br>1. To determine the perfusion and vascular volume fraction of measurable tumor lesions versus normal reference tissue before and after tTF-NGR application by MRI as a biological surrogate parameter for biological activity of the IMP in the patients treated within the verification cohorts.<br><br>2. To obtain pharmacokinetic data of tTF-NGR.
**Endpoints:**	Primary Safety Endpoint:<br><br>Maximum Tolerated Dose (MTD). MTD is the dose below the dose leading to reproducible episodes of Dose Limiting Toxicity (DLT) in at least 2/6 patients. DLT will be characterized by clinical, blood and serum monitoring at specified time points before and during study period.<br><br>Secondary Efficacy Endpoints:<br><br>1. Occurrence and frequency of inhibition of tumor blood flow as measured by contrast-enhanced Magnetic Resonance Imaging (MRI) in the patients treated in the verification cohorts.<br>2. Occurrence and frequency of tumor response defined as Complete Response (CR), Partial Response (PR), or Stabile Disease (SD) as defined by RECIST criteria.<br>Descriptive statistics will be used.
**Planned Number of Study Centers:**	1
**Number of Patients/ Sample size:**	A minimum of 7 patients depending on occurrence of DLT.
**Inclusion Criteria:**	➢ age $\geq$ 18 years<br><br>➢ histologically proven or cytologically confirmed solid malignant tumor or malignant lymphoma<br><br>➢ recurrent or refractory disease after standard therapy and with no known curative or survival-prolonging

Cancers **2020**, *12*, x; doi: www.mdpi.com/journal/cancers

	treatment options according to the judgement of the investigators<br><br>➢ life expectancy of at least 6 weeks according to the judgement of the investigators<br><br>➢ Karnofsky performance status ≥50<br><br>➢ measurable disease with at least one marker-lesion measurable in 2 dimensions by Vascular Volume-Fraction-MRI<br><br>➢ adequate bone marrow function with absolute neutrophil count > 1000/microliter and platelet count > 50/nl.<br><br>➢ normal global coagulation parameters (Quick, PTT, TZ, fibrinogen), no prophylactic anticoagulation<br><br>➢ adequate liver function (total bilirubin < 3x the upper normal limit (ULN), SGPT/SGOT < 3x ULN)<br><br>➢ adequate renal function (serum creatinine < 3x ULN)<br><br>➢ no history of coronary heart disease, stroke, transitory ischemic attacks, pulmonary embolism, or deep vein thrombosis<br><br>➢ time elapsed from previous therapy (including other IMPs) ≥ 3 weeks with recovery from side effects<br><br>➢ exclusion of central nervous system (CNS) disease and CNS vascular abnormalities by MRI<br><br>➢ ability to understand and provide written informed consent<br><br>➢ written informed consent given<br><br>➢ for female patients with child-bearing potential exclusion of pregnancy by adequate testing within 48 hours prior to entry on study<br><br>➢ females of childbearing potential as well as fertile males must agree to use a highly effective form of contraception (Pearl Index < 1) during the study and for 120 days following the last dose of the IMP
**Exclusion Criteria:**	➢ clinically significant unrelated illness which in the judgement of the investigators could compromise the patient's ability to tolerate the IMP or be likely to interfere with the study procedures or results

Cancers **2020**, *12*, x; doi: www.mdpi.com/journal/cancers

- known hypersensitivity reactions to prior application of *E. coli*-derived material
- women with breast-feeding activity
- concomitant use of any other investigational agent (agent for which there is currently no approved indication from regulatory authorities)
- clinical application of any other drug with known antitumor activity
- prophylactic anticoagulation within the last 3 days

Visits:

Days (d), hours (h), weeks (w) post IMP	pre (within 1 w)	at inf. stop	1 h	5 h	daily	5 d	weekly	repeat cycles (see left)	monthly from end of IMP application	study period end
History + examination	x	x	x	x	x	x	x		x	x
Lab tests (FBC, biochemistry, coagulation)	x	x	x	x	x	x	x		x	x
Oxygen sats (- = continuous), ECG-monitoring	x	x-	x-	x-	x	x	x		x	x
Investigator to check eligibility	x						x			
Informed consent	x									
Clinical/blood assessment of AE and toxicity	x	x	x	x	x	x	x		x	x
Tumor measurements	x								x (without relation to IMP)	x
MRI, vascular volume fraction*	x			x		x				(x)
Pharmacokinetics	x	x	x (see below)	x	x	x	x		x	
HAHA						x				x
CNS-MRI	x									

**, times are selected by the decision of the investigator, patients in the verification groups are to be evaluated at least in one cycle of therapy*

Statistical Methods:

Descriptive statistics are performed to characterize MTD (= dose level below DLT occurring in 2/6 patients)

Financial Support:

Deutsche Krebshilfe, grant 111004

Schedule

Planned start date of the study (FPFV): 01.11.2016
Planned end date of recruitment (LPFV): 01.11.2017
Planned end date of the study (LPLV): 01.05.2018

Planned recruitment duration: 1 year
Duration of single patient participation: according to tolerability and antitumor activity
Planned overall duration of the study: 1.5 years

Cancers **2020**, *12*, x; doi: www.mdpi.com/journal/cancers

3. Protocol overview

3.1 Title of Study

PHASE I STUDY OF tTF-NGR IN PATIENTS WITH RECURRENT OR REFRACTORY MALIGNANT TUMORS OR LYMPHOMAS BEYOND ALL STANDARD TREATMENTS

3.2 Protocol summary

Every year worldwide more than 8 million humans die from malignant diseases. The incidence of malignant diseases is increasing. Surgery, radiotherapy and systemic treatment with chemotherapy and immunotherapy have measurable therapeutic effects, but about half of the patients with cancer develop metastasis and have no curative outlook. There is an unmet need for new anticancer treatment strategies using innovative mechanisms of action.

Antiangiogenesis therapy is a relatively new treatment approach for metastatic cancer. It interferes with the development of new vessel formation (neo-angiogenesis), which is essential for the spread of cancer. First agents, such as antibodies targeting vascular endothelial growth factor (VEGF) and small molecule receptor tyrosine kinase inhibitors have been shown to increase overall survival of patients with various tumor histologies for some months. Some agents are approved for medical use in cancer patients. Vascular targeting agents or vascular disrupting agents interfere and destroy already existing tumor vessels. Although interesting results from experimental and early clinical studies exist, no such agent is approved for clinical use.

This document describes a clinical phase I study with an investigational medical product (IMP) inducing thrombosis of blood vessels in solid tumors by a fusion protein consisting of the extracellular domain of tissue factor (truncated tissue factor, tTF) and the peptide GNGRAHA, targeting aminopeptidase N (CD13) and the integrin $\alpha_v\beta_3$ (CD51/CD61) on tumor vascular endothelium. The designed fusion protein tTF-NGR retains its thrombogenic activity and demonstrated in vivo antitumor activity through partial or complete thrombotic occlusion of tumor vessels in human tumor xenograft models. Clinical first-in-man application of low dosages (up to 4 mg/m^2) of this targeted coagulation factor revealed good tolerability and decrease of tumor perfusion as measured by magnetic resonance imaging (MRI).

Cancers **2020**, *12*, x; doi: www.mdpi.com/journal/cancers

In this phase I clinical trial cancer patients suffering from solid tumors or lymphomas, recurring after and/or refractory against standard treatment are treated intravenously (iv) with increasing doses of tTF-NGR. The objectives of this trial are to evaluate the maximum tolerated dose (MTD) and the dose-limiting toxicity (DLT) of intravenously (iv) infused daily applications of tTF-NGR for 5 days every 3 weeks in patients with cancer, who had obtained all standard treatment known for their disease entity prior to entry on study. Further objectives are to determine the perfusion and vascular volume fraction of measurable tumor lesions versus normal reference tissue before and after tTF-NGR application by MRI as a biological surrogate parameter for biological activity of the IMP and to obtain pharmacokinetic data.

3.3 Trial Objectives

Primary Objectives

To evaluate the maximum tolerated dose (MTD) and the dose-limiting toxicity (DLT) of intravenously (iv) infused daily applications of tTF-NGR for 5 days every 3 weeks in patients with cancer (solid tumors and lymphomas), who had obtained all standard treatment known for their disease entity prior to entry on study.

Secondary Objectives

1. To determine the perfusion and vascular volume fraction of measurable tumor lesions versus normal reference tissue before and after tTF-NGR application by MRI as a biological surrogate parameter for biological activity of the IMP. Imaging will be preferentially done in the patients within the verification cohorts in at least one of the treatment cycles as determined by the investigator.

2. To obtain pharmacokinetic data of tTF-NGR.

3.4 Investigational Medicinal Product

The IMP is a fusion peptide of N-terminally histidine-tagged human truncated tissue factor (amino acids 1-218) and a C-terminal GNGRAHA binding peptide cloned into the expression vector pET-30(+)a (Novagen), generated by *Escherichia coli* (BL21 DE3) and purified by high performance liquid chromatography (HPLC). Details are summarized in the accompanying Investigational Medicinal Product Dossier (IMPD).

Cancers **2020**, *12*, x; doi: www.mdpi.com/journal/cancers

3.5 Trial design

Mono-center, non-randomised, open-label, multiple-dose level phase I trial. The study will be performed in the University Hospital Muenster (UKM). Patients will be treated with tTF-NGR by 1-hour iv infusions via central venous access daily for 5 days every 3 weeks. Starting dose will be 1 mg/m^2/day (for justification of this starting dose see IMPD). Dose escalation will be performed within a single patient in 0.5 mg/m^2/day steps upon good toleration of the previous cycle. Patients within the dose-escalation part will be treated in sequence and not in parallel. Individual patients can undergo a maximum of 8 dose-escalations. The maximum number of 8 dose escalations is reached only if stable disease (SD) is ongoing upon 8 cycles of therapy. Dose-escalation is stopped before the maximum number of 8 escalation steps if tumor response, tumor progression or a Dose-Limiting Toxicity (DLT) is observed. In the case of SD and with good tolerability these patients can obtain further cycles without dose-escalation until tumor progression or Dose-Limiting Toxicity (DLT) and the next patient can start with dose-escalation cycles on the highest tolerable dose for the previous patient. A patient will undergo no further treatment in case of progressive disease or DLT, unless the DLT is resolved in a responding patient.

Treatment will be followed by MRI tumor perfusion studies before treatment onset, on day 1 (4-6 hours after 1st application), and on day 5. Omission of MRT monitoring during single cycles is allowed as decided by the Investigator with the aim to concentrate MRT monitoring on the higher dose levels, in particular in the patients of the verification cohorts (see below).

Pharmacokinetic monitoring will be performed as outlined below.

3.6 Risk-Benefit Assessment

Every year worldwide more than 8 million humans die from malignant diseases. The incidence of malignant diseases is increasing. Surgery, radiotherapy and systemic treatment with chemotherapy and immunotherapy have measurable therapeutic effects, but about half of the patients with cancer develop metastasis and have no curative outlook. There is an unmet need for new anticancer treatment strategies using innovative mechanisms of action. Patients accrued for this trial are suffering from relapsed or refractory solid tumors or lymphomas after standard therapy with no option

Cancers **2020**, *12*, x; doi: www.mdpi.com/journal/cancers

for further standard treatment leading to cure or prolongation of overall survival as judged by the investigator.

The IMP, tTF-NGR, was developed as a new bifunctional protein carrying an essential pro-coagulatory molecule – tissue factor (TF) – via a C-terminal peptide (GNGRAHA) into tumor vessels to induce *selective tumor vascular infarction*. To this end the non-specific membrane anchor of TF was replaced by the CD13-binding NGR motif. CD13 is an aminopeptidase selectively occurring on stimulated and growing endothelial cells (EC), as on tumor endothelial cells, with only limited expression on normal tissue (http://www.proteinatlas.org/ENSG00000166825-ANPEP/tissue). With this molecule we target and accumulate TF in tumor vasculature and induce tumor vascular infarction leading to tumor cell death. tTF-NGR showed robust *in vitro* and *in vivo* activity. tTF-NGR retains complete procoagulatory activity, specifically binds to CD13 on growing EC, it leads to tumor vessel infarction and occlusion with resulting tumor growth inhibition and regression in experimental models. Therapeutic activity of tTF-NGR was independent of tumor histology (e.g. melanoma, lung, breast, sarcoma). Repeated rounds of treatment showed no resistance development. Xenograft experiments in the mouse showed a safe therapeutic window.

The IMP tTF-NGR will be given to few patients with intraindividual dose escalation in sequence until DLT is observed. Subsequently, 5 further patients will be treated sequentially within a verification cohort for DLT. In case a deadly DLT occurs, the verification cohort will be sequentially dosed at the next-lower dose level for safety reasons. Exact dose-escalation is described below. MTD is defined as the dose below observation of 2/6 patients with DLT. MTD dose level will then be verifyed by treating 5 further patients on this dose level below the one on which DLT was observed. This will allow to reduce the risk of DLT to few individual patients and simultaneously avoid treating any patient at dose level so low, that antitumor activity can not occur.

All patients (minimum 7 patients) treated within this protocol can hypothetically reach tumor control by the IMP with a minimum of only 2 patients at risk for DLT. Thus, the potential benefit for the individual patients treated within this study outweighs the risks.

4. *Background and Rationale*

Cancers **2020**, *12*, x; doi: www.mdpi.com/journal/cancers

The scientific background and rationale for performing this phase I trial and the complete set of information on the IMP tTF-NGR are outlined in detail in the Investigational Medicinal Product Dossier (IMPD) on tTF-NGR.

5. Study Design

This is an open-label, single-center phase I study of tTF-NGR in patients with relapsed and/or refractory solid tumors and lymphomas.

Patients will be treated with tTF-NGR by 1-hour iv infusions via central venous access daily for 5 days every 3 weeks. Starting dose will be 1 mg/m^2/day. Intraindividual dose escalation will be performed within one single patient in 0.5 mg/m^2/day steps upon good toleration of the previous cycle. Patients within the dose-escalation part will be treated in sequence and not in parallel. Individual patients can undergo a maximum of 8 dose-escalations. The maximum number of 8 dose escalations is reached only if stable disease (SD) is ongoing upon 8 cycles of therapy. Dose-escalation is stopped before the maximum number of 8 escalation steps if tumor response, tumor progression or a Dose-Limiting Toxicity (DLT) is observed. In the case of SD and with good tolerability these patients can obtain further cycles without dose-escalation until tumor progression or Dose-Limiting Toxicity (DLT) and the next patient can start with dose-escalation cycles on the highest tolerable dose for the previous patient. A patient will undergo no further treatment if any of the criteria under 7.4 is reached.

Treatment will be followed by MRI tumor perfusion studies at cycles as decided by the Investigator as a surrogate marker for target-hit. MRI tumor perfusion studies will be performed before treatment onset, on day 1 (4-6 hours after application), and on day 5. MRI will be done not in every cycle of every patient, but in every patient of the verification cohorts in at least one cycle.

Toxicity monitoring will be performed according to the most recent version of the CTC criteria.

MTD is the dose below the dose leading to reproducible episodes of Dose Limiting Toxicity (DLT). DLT will be characterized by clinical, blood and serum monitoring at specified time points before and during study period.

Antitumor activity and response will be determined according to RECIST criteria with extent of disease evaluation before and after treatment period and in 3-monthly intervals thereafter, or as defined by clinical needs. Waterfall-analysis for response quantification in % of the marker lesions before treatment onset and at best response will be done centrally by the study radiologist.

Pharmacokinetic monitoring will be done as outlined below.

6. Eligibility criteria and patient registration

6.1 Inclusion Criteria

Inclusion criteria for entry on trial are

- age $\geq$ 18 years
- histologically proven or cytologically confirmed solid malignant tumor or malignant lymphoma
- recurrent or refractory disease after standard therapy and with no known curative or survival-prolonging treatment options according to the judgement of the investigators
- life expectancy of at least 6 weeks according to the judgement of the investigators
- Karnofsky performance status $\geq$ 50
- measurable disease with at least one marker-lesion measurable in 2 dimensions by Vascular Volume-Fraction-MRI
- adequate bone marrow function with absolute neutrophil count > 1000/microliter and platelet count > 50/nl.
- normal global coagulation parameters (Quick, PTT, TZ, fibrinogen), no prophylactic anticoagulation
- adequate liver function (total bilirubin < 3x the upper normal limit (ULN), SGPT/SGOT < 3x ULN)
- adequate renal function (serum creatinine < 3x ULN)
- no history of coronary heart disease, stroke, transitory ischemic attacks, pulmonary embolism, or deep vein thrombosis
- time elapsed from previous therapy (including other IMPs) $\geq$ 3 weeks with recovery from side effects

- exclusion of central nervous system (CNS) disease and CNS vascular abnormalities by MRI
- ability to understand and provide written informed consent
- written informed consent given
- for female patients with child-bearing potential exclusion of pregnancy by adequate testing within 48 hours prior to entry on study
- females of childbearing potential as well as fertile males must agree to use a highly effective form of contraception (Pearl Index < 1) during the study and for 120 days following the last dose of the IMP

6.2 Exclusion criteria

- clinically significant unrelated illness which in the judgement of the investigators could compromise the patient’s ability to tolerate the IMP or be likely to interfere with the study procedures or results
- known hypersensitivity reactions to prior application of *E. coli*-derived material
- women with breast-feeding activity
- concomitant use of any other investigational agent (agent for which there is currently no approved indication from regulatory authorities)
- clinical application of any other drug with known antitumor activity
- prophylactic anticoagulation within the last 3 days

NOTE: Since this is a phase I study for end-stage cancer patients, patients who would be excluded from the protocol strictly for laboratory abnormalities only can be included at the Investigator’s discretion. This will be documented as an exception to the criteria and will be signed and filed in the CRF and the Trial Master File.

Patient’s eligibility for entry to the study will be assessed by the Investigator or their delegated representative physician. Distribution of **gender** in the study population will be random and gender will be no factor influencing accrual. tTF-NGR is not expected to differ in its effects in male and female patients.

Patients identified as eligible for entry into the study will be **registered** centrally through the study bureau of the Department of Medicine A (Hematology, Hemostaseology and Oncology) of the UKM.

7. Treatment Plan

7.1 Administration of tTF-NGR

Patients will receive their assigned dose of tTF-NGR as an i.v. infusion via central venous access (PORT-systems allowed) with an infusion duration of 1 hour at a constant rate. The IMP is stored at -80°C and will be thawed, solved in saline and filtered (Millipore filter unit, 0.2 µm) short before application (see Appendix: Phase I: Rekonstitution tTF-NGR).

Potential side effects and toxicities anticipated can be classified as those related to infusional reactions, venous thrombosis, arterial vascular occlusion and embolism, disseminated intravascular coagulation (DIC), tumor bleeding, antibody formation, liver toxicity, and transient thrombocytopenia (see IMPD). Thus, treatment has to be performed obligatory in-house for the complete application time of 5 days with all appropriate drugs for hypertensive reactions, for fibrinolysis and anticoagulation ready to use and with appropriate transfusions of platelets and red blood cells available and prepared for. Constant back-up and information of the Intensive Care Unit (ICU) of the UKM is mandatory.

7.2 General concomitant medication and supportive care

Concomitant therapy and supportive care will be provided as deemed necessary by the Investigators and according to the institutional guidelines.

7.3. Other anti-cancer medication

Administration of any other anti-cancer medication including chemotherapy and biologic therapy and other IMPs is not permitted during study period. Palliative radiotherapy of single tumor-lesions e.g. causing tumor-related pain is allowed, however, these lesions have to be excluded from response judgement.

7.4 Duration of therapy and Study Period

Treatment with tTF-NGR may continue until one of the following criteria applies:

- disease progression

Cancers **2020**, *12*, x; doi: www.mdpi.com/journal/cancers

- intercurrent illness that prevents further administration of treatment according to the judgement of the Investigators
- Occurrence of Dose Limiting Toxicity (DLT), i.e., unacceptable adverse event(s), including Grade $\geq$ 3 (CTCAE) toxicity for any organ with the exception of the hematopoietic system or Grade 4 for the hematopoietic system attributed to the IMP
- patient decides to withdraw from the study
- general or specific changes in the patient´s condition rendering the patient unfit for further treatment in the judgement of the Investigators
- patient is lost to follow-up
- death

Duration of Study Period with follow-up is defined as being

- from day of signature of Informed Consent to
- day 5 after the start of the last dose level plus 3 months of observation

Patients will be removed from study treatment when any of the criteria listed under section 7.4 applies or the Study Period is completed.

In patients with objective response and good toleration on a specific dose-level reached, or with stable disease upon 8 cycles of therapy, individual dose-escalation will be stopped and patients will be further treated for up to 3 months with the specific dose under which response occurred, or in case of stable disease with the dose at the 8th cycle. If this patient is treated with dose-escalation steps, he/she will be replaced by the next patient to continue the dose-escalation. This is to not add further risk to an individual, when individual benefit is visible. Upon this time Study Period in these individual patients will end. These patients will be eligible for further compassionate-use treatment with the IMP on the dose-level after completion of the Study Period as long as they benefit from treatment in accordance with §34 of the Declaration of Helsinki (2013 Fortaleza, Brazil). Serious adverse events (see paragraph 13 of this protocol) occurring during compassionate use treatment will be reported as within the defined Study Period to guarantee patient safety within this trial.

8. *Dosing delays and dose de-escalations in individual patients*

De-escalation in individual patients in dosing is not allowed during the Study Period to not interfere with the description of Dose Limiting Toxicity (DLT) and MTD.

Delays in dosing of the next application for up to 6 days in individual patients are allowed in case anticipated adverse events connected with the composition of the drug or the mechanism of action of tTF-NGR occur, which need to be addressed before treatment can continue. This is in particular foreseen in the following situations:

1. Consumption of platelets due to tumor vascular coagulation with platelet count drops to grade 3 of CTCAE version 4.0 (page 112; 25.000 – 50.000 platelets per microliter) to allow recovery of counts to >50.000 per microliter,

2. Infusional reactions due to the protein application in case tTF-NGR was applied without premedication (see below) to allow resolution to CTCAE grade <2 and a following application with premedication.

However, in patients with objective response and good toleration of the drug, who are further treated in the compassionate-use program with the IMP after completion of the Study Period, dosing delays and/or modifications are allowed and handled according to the judgement of the Investigators.

9. *Dose Limiting Toxicity (DLT) and Maximum Tolerated Dose (MTD)*

Dose Limiting Toxicity (DLT): DLT is defined as occurrence of toxicity of Grade 3 or more according to the revised NCI Common Terminology Criteria for Adverse Events (CTCAE) version 4.0 (http://ctep.cancer.gov) for non-hematological toxicity or Grade 4 or more for hematological toxicity. DLT must be considered by the investigator as being causally related to the IMP. In case the investigator comes to no decision, the data safety monitoring board is to be involved. Intensity of toxicity will be graded according to CTCAE using a five-point scale:

Cancers **2020**, *12*, x; doi: www.mdpi.com/journal/cancers

CTC Grade	Equivalent To:	Definition
Grade 1	Mild	Mild; asymptomatic or mild symptoms; clinical or diagnostic observations only; intervention not indicated.
Grade 2	Moderate	Moderate; minimal, local or noninvasive intervention indicated; limiting age-appropriate instrumental Activities of Daily Living (ADL).
Grade 3	Severe	Severe or medically significant but not immediately life-threatening; hospitalization or prolongation of hospitalization indicated; disabling; limiting self care ADL.
Grade 4	Lifethreatening/Disabling	Life-threatening consequences; urgent intervention indicated
Grade 5	Death	Death related to AE.

When DLT is observed in the 1st patient, up to 5 more patients will be treated on the same dose level, or in case of deadly DLT in the dose level below (verification group for DLT; maximum of 6 patients on this level). Upon observation of no additional DLT after treating 6 patients on this dose level dose escalation can be resumed in one patient. The remaining 5 patients can be treated without dose-escalation until tumor progression or DLT. Upon observation of one more episode of any DLT within the verification group, patient accrual to this verification group is stopped with this second patient with DLT and de-escalation by a 0.5 mg/m^2/day step is performed in 5 further patients until ≤1 DLT occurs in 5 further patients treated on a specific dose-level, i.e. with ≤1/6 patients in total with DLT on this dose level (scheme see below).

Maximum Tolerated Dose (MTD): MTD is the dose below the lowest dose level on which reproducible episodes of any DLT are observed. Reproducible episodes of DLT have also to be stated if DLT occurs in different organ systems of individual patients.

10. Dose escalation and de-escalation according to toxicity and dose recommended for phase II trials (RDPII)

The patients will be treated daily with tTF-NGR by 1-hour iv infusions for 5 days with MRI studies before, 4-6 hours after the 1st application, and on day 5 after treatment as decided by the Investigator.

Starting dose will be 1 mg/m^2/day. Intraindividual dose-escalation will be performed in 1 patient in 0.5 mg/m^2/day steps (to a maximum of 8 cycles) upon a rest period of 16 days for the next treatment cycle of 5 days. The maximum number of 8 dose escalations is reached only if stable disease (SD) is ongoing upon 8 cycles of therapy. Dose-escalation is stopped before the maximum number of 8 escalation steps if tumor response, tumor progression or a Dose-Limiting Toxicity (DLT) is observed. If tumor response is observed or if a patient is diagnosed with progressive disease (PD) after $\geq$ 2 cycles or clinically as judged by the investigator even before 2 cycles, this patient will go off study and will be replaced by a further patient in whom individual dose escalation will be resumed, starting with the highest dose level reached without DLT in the previous patient. When any DLT is observed in this patient, intraindividual dose escalation is stopped and the patient is taken off study. In case of stable disease or response and resolution of the DLT this patient can be further treated on the highest tolerable dose level without DLT until any of the criteria under 7.4 is reached. This treatment will then be given on an individual compassionate-use basis (off study) according to § 34 of the Helsinki declaration (2013, Fortaleza; Post-Trial Provision).

If this first observed DLT is nonfatal, up to 5 more patients will then be treated on the same dose level having caused DLT (verification group). Patients of the verification group will be treated in sequence of each other, thus a patient has to tolerate the first cycle (5 days) and the rest period of 16 days without DLT before the next patient can be added to this verification group.

Upon observation of no additional DLT in the verification group in a minimum of 2 cycles for each patient, dose escalation can be resumed in one patient out of these until DLT (continue as above). The remaining patients in the verification group will be further treated at this dose-level until any of the criteria under 7.4 is reached.

Cancers **2020**, *12*, x; doi: www.mdpi.com/journal/cancers

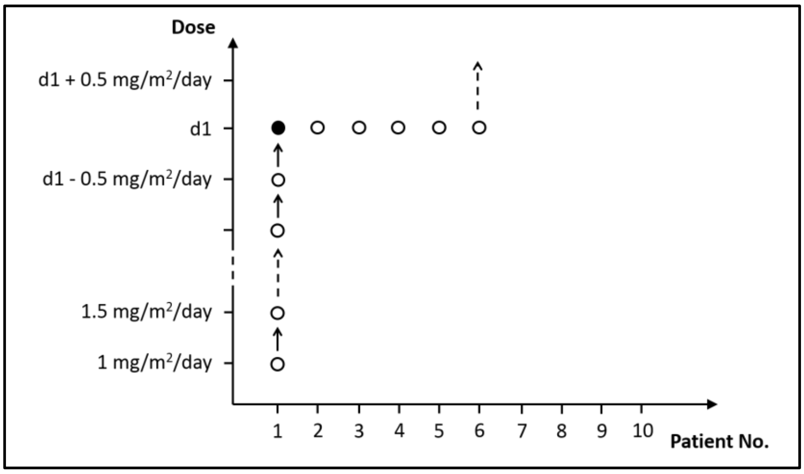

Upon observation of one more episode of any DLT within the first 2 cycles given to the patients in the verification group, patient accrual to this verification group is stopped with this second patient with DLT and de-escalation by a 0.5 mg/m^2/day step is performed in further up to 5 patients. If on the next lower dose level there are ≥2 DLT in 5 further patients (i.e., ≥2 DLT in 6 patients in total on this dose level), de-escalation will continue. In case, patients of the verification group experience DLT in a later cycle (3rd and following), this will be counted as DLT with all consequences, although the procedure of the study was not influenced before such an event occurred.

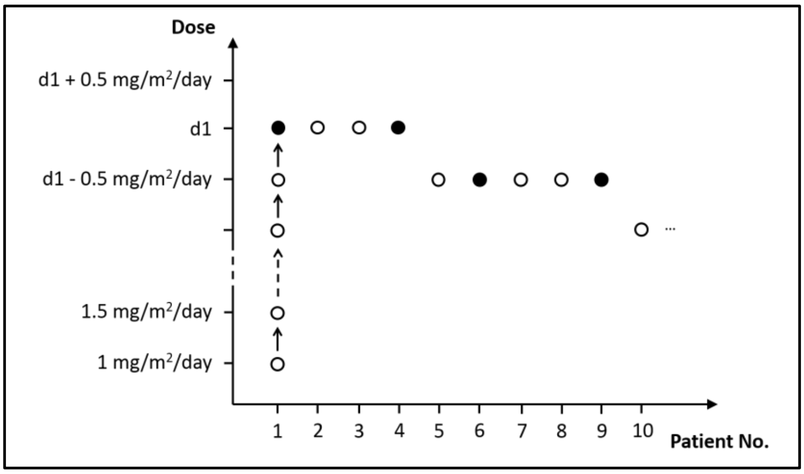

Dose de-escalation will continue until ≤1 DLT occurs in 5 further patients treated on a specific dose-level, i.e. there are ≤1/6 patients with DLT in total on this dose level. This dose level is declared the MTD and will be recommended for further studies, such as phase II trials (RDPII).

If an observed DLT is fatal, the above dose escalation scheme is modified. Denote this dose d_{fatal}. In the unlikely event that further patients are treated with this dose, treatment in these patients will be stopped at this dose level and the investigator decides how to go on with the treatment in these patients. The next patients will not be treated with the dose d_{fatal}, but on the next lower dose level d_{fatal} - 0.5 mg/m^2/day. If on the dose level d_{fatal} - 0.5 mg/m^2/day no DLT or not more than 1 DLT is observed that is non-fatal, further patients are treated on the dose level d_{fatal} until any further DLT is observed or 6 patients are included on this dose level in total. Possible later dose escalation to a level at which one death occurred has to be discussed with the data safety board.

Only in the very unlikely event, that DLT occurs at the starting dose or the dose 0.5 mg/m^2/day higher, further dose de-escalation below the starting dose level can be performed as described to establish MTD.

The above procedure is constructed so that MTD is defined similarly to a standard 3+3 design. I.e., any dose with observed DLT in ≤1/6 patients is defined to be the MTD, if on the next higher dose level the observed number of patients with DLT is ≥2/6. The following summary visualizes this procedure:

Start 1 pt at 1 mg/m^2/day with individual dose escalation at 0.5 mg/m^2/day for 5 days every 3 weeks until any DLT (= dose “d1”) or to a max of 8 cycles

➢Verification group (VG) of ≤5 further pts at d1 without indiv. dose esc.

(➢Verification group (VG) of ≤5 further pts at d1-0.5mg/m^2/day without indiv. dose esc. in case a death occurs as DLT)

➢No further DLT in VG ➢resume dose escalation: start 1 pt of VG at d1 + 0.5 mg/m^2/day (d), then as above

➢1 further DLT in VG ➢ dose de-escalation d1 - 0.5 mg/m^2/d, 5 further pts (d-1(-2))

➢ ≤1 further DLT: d-1 = MTD

➢>1 further DLT ➢dose de-escalation, continue as above

Again, if a patient has been taken off the study due to a DLT or progressive disease, the patient may further be treated as follows. If the patient has stable disease or response and the DLT has been resolved, outside the study the patient may be treated

with the study medication on the highest tolerable dose level without DLT (§ 34 Declaration of Helsinki, Fortaleza 2013). In case of progressive disease or if the DLT has not been resolved, different treatment options are offered.

11. Study objectives and end-points

Primary Objectives

To evaluate the maximum tolerated dose (MTD) and the dose-limiting toxicity (DLT) of intravenously (i.v.) infused daily applications of tTF-NGR in patients with cancer (solid tumors and lymphomas), who had obtained all standard treatment known for their disease entity prior to entry on study.

Secondary Objectives

1. To determine the perfusion and vascular volume fraction of measurable tumor lesions versus normal reference tissue before and after tTF-NGR application by MRI as a imaging surrogate parameter for biological activity of the IMP.

2. To obtain pharmacokinetic data of tTF-NGR.

Primary Safety end-point

Maximum Tolerated Dose (MTD). MTD is the dose below the dose leading to reproducible episodes of Dose Limiting Toxicity (DLT) in at least 2/6 patients. DLT will be characterized by clinical, blood and serum monitoring at specified time points before and during study period.

Secondary Safety end-points

1. Occurrence and frequency of inhibition of tumor blood flow as measured by contrast-enhanced Magnetic Resonance Imaging (MRI).
2. Occurrence and frequency of tumor response defined as Complete Response (CR), Partial Response (PR), or Stabile Disease (SD) as defined by RECIST criteria.

Descriptive statistics will be used.

Cancers **2020**, *12*, x; doi: www.mdpi.com/journal/cancers

12. Patient evaluation and follow-up

Patients will be evaluated as outlined in the study calendar below. The time-points given are in relation to IMP application. Additional visits will occur as clinically indicated.

Study Calendar for each 5-day cycle

Days (d), hours (h), weeks (w) post IMP	pre (within 1 w)	at inf. stop	1 h	5 h	daily	5 d	weekly	repeat cycles (see left)	monthly from end of IMP application	study period end
History + examination	x	x	x	x	x	x	x		x	x
Lab tests (FBC, biochemistry, coagulation)	x	x	x	x	x	x	x		x	x
Oxygen sats (- = continuous), ECG-monitoring	x	x-	x-	x-	x	x	x		x	x
Investigator to check eligibility	x						x			
Informed consent	x									
Clinical/blood assessment of AE and toxicity	x	x	x	x	x	x	x		x	x
Tumor measurements	x								x (without relation to IMP)	x
MRI, vascular volume fraction*	x			x		x				(x)
Pharmacokinetics	x	x	x (see below)	x	x	x	x		x	
HAHA						x				x
CNS-MRI	x									

**, times are selected by the decision of the investigator, patients in the verification groups are to be evaluated at least in one cycle of therapy*

12.1 History, physical examination and summary of laboratory investigations

A full **history** and **physical examination** will be done pre-IMP infusion, and thereafter as indicated in the study calendar. Examination also comprises statement on the performance score (Karnofsky index) of the patient and an ECG. ***Lab tests*** comprise a full blood count, urea, creatinine, electrolytes, glucose, protein, lipids, bilirubin, ALT (SGOT), AST (SGPT), AP, LDH, PCHE, ferritin, haptoglobin, CPK, troponin, lactate, standard urinalysis and coagulation assays. Coagulation assays comprise Quick, PTT, TZ, fibrinogen, fibrin-split-products, alpha 2 AP, AT III, D-dimers. Human anti-human antibodies (HAHA) will be tested for as indicated.

12.2 Clinical/blood assessment of toxicity

Clinical and blood assessment of toxicity will be done in intervals given in the study calendars during study period according to NCI CTCAE as stated above in section 9 of this protocol.

12.3 Pharmacokinetics

For pharmacokinetic studies the IMUBIND® assay will be used according to the protocol given by the manufacturer. Blood samples will be collected at the time points indicated in the calendars and stored frozen until testing. Since there is data from the first testings in patients treated that the half-life of tTF-NGR ($t_{1/2}$) is in the range of few hours, the time point given as "1h" in the calendars will be indeed multiple samplings in 30 min intervals for the first 5 hours.

12.4 Adverse events and unexpected toxicities

All adverse events will be assessed by the Investigator for their seriousness, expectedness and relationship to the study protocol. If appropriate, the IMPD will be referenced. Anticipated toxicities are listed in the IMPD. If the tTF-NGR is suspected of causing unexpected serious adverse reactions, SUSAR reporting will be instigated as in section 13 below and the Investigator and sponsor informed immediately. It is the Sponsor's responsibility to report any SUSARs to the Bundesoberbehörde Paul-Ehrlich-Institute complying with the mandated timelines (7 days for fatal and life-threatening events and within 15 days for all others). Additional information will be provided as relevant within the 8 day designated time-frame. The Sponsor will also be responsible for reporting to the Regulatory Authorities, Ethics Committees and Principal Investigators as per current legislation.

12.4.1 Data and safety monitoring board (DSMB) and Trial Steering Committee (TSC)

The Data and Safety Monitoring Board (DSMB) comprises 3 clinicians with appropriate expertise, all of whom are independent of the sponsor and have no involvement in the development of the protocol. The DSMB will consist of Univ.-Prof. Dr. Ulrich Dührsen (Director, Department of Hematology, University Hospital Essen), Prof. em. Dr. Normann Willich (Geschäftsführer, Deutsche Ges. für Radioonkologie), and Prof. Dr. Carsten Bokemeyer (Director Department of Medicine – Hematology and Oncology, University Hospital Hamburg). Procedures governing the convening and execution of

Cancers 2020, 12, x; doi: www.mdpi.com/journal/cancers

the responsibilities of the DMC will be specified separately in a written DMC Charter. The DSMB will review data on recruitment, safety and data collection and will advise the Trial Steering Committee (TSC) on whether it is appropriate for the trial to continue. The TSC will consist of Prof. Dr. Wolfgang E. Berdel (Director Department of Medicine A, UKM), Prof. Dr. Rolf Mesters (Co-Investigator), and Prof. Dr. Andreas Faldum (Trial statistician).

13. Reporting requirements

All patients will be registered on the study enrollment log and data on study end-points will be kept on Case Report Forms (CRFs) for each subject.

13.1 Definitions of Adverse Events

Adverse Events are defined according to the Directive 2001/20/EC, the European Detailed Guidance CT 3, corresponding to the relevant definitions in the German GCP Ordinance.

Adverse Event (AE)

This is defined as any untoward medical occurrence in the patient administered an investigational medicinal product that does not necessarily have a causal relationship with this treatment. An AE is therefore described as any unfavorable and unintended sign (including abnormal laboratory results), symptom or disease temporally (timely) associated with the use of an investigational medicinal product, whether or not related to the product.

Clarification:

- A pathological finding, improved or unchanged in comparison to its status before first administration of the investigational medicinal product, does not constitute an adverse event.
- Symptoms of the disease under study should not be classified as AEs as long as they are within the normal day-to-day fluctuation or expected progression of the disease, and as long as they are not serious.

- Laboratory test value abnormalities will be reported in the CRF. Abnormal values should not be reported on the AE page of the CRF unless they are associated with a clinically relevant condition.

Adverse Reaction (AR)

An adverse reaction is defined as any untoward and unintended response in a subject to an investigational medicinal product, related to any dose administered. A causal relationship between the investigational medicinal product and the adverse event is at least a reasonably possibility, which means that there are facts or arguments to suggest a causal relationship.

The definition covers also medication errors and uses outside what is foreseen in the protocol, including misuse and abuse of the product.

Serious Adverse Event (SAE)

A serious adverse event is defined as any untoward medical occurrence in the patient administered a pharmaceutical product, which does not necessarily have a causal relationship with this treatment, and that at any dose:

- ➢ results in death
- ➢ is life threatening (at immediate risk of death at the time of the event)
- ➢ results in persistent or significant disability/incapacity
- ➢ requires in-patient hospitalization or prolongs existing hospitalization*
- ➢ results in a congenital abnormality or birth defect
- ➢ is medically significant (i.e. any event which the investigator considers significant but which is not covered by the above.)

*: Hospitalization means overnight admission. Hospitalization without underlying adverse event (AE) is not an SAE. Examples are:

- Hospitalization for protocol procedures e.g. chemotherapy, biopsy or monitoring of the study
- Elective hospitalization for a pre-existing condition (i.e. a condition other than

the indication for the chemotherapy) that has not worsened

- Admission to a rehabilitation centre or hospice.

Unexpected Adverse Reaction

"Unexpected" means that the nature, severity or outcome of the adverse reaction is not consistent with the applicable product information for the investigational medicinal product, which is the current version of the IMPD.

- The term "severe" is often used to describe the intensity (severity) of a specific event. This is not the same as "serious," which is based on patient/event outcome or action criteria.
- Reports which add significant information on the specificity, increase of occurrence, or severity of a known, already documented serious adverse reaction constitute unexpected events.

Suspected Unexpected Serious Adverse Reaction (SUSAR)

A suspected unexpected serious adverse reaction (SUSAR) is a serious adverse reaction that has been judged to be unexpected.

Severity

Severity for each adverse event, including any lab abnormality, will be determined by using the National Cancer Institute Common Toxicity Criteria (NCI CTCAE, version 4.0) as a guideline, wherever possible. The criteria are available online at http://ctep.cancer.gov/reporting/ctc.html. In those cases where the NCI CTC criteria do not apply, severity should be defined according to the general CTCAE definitions of grades (see chapter 9).

Causality

Relationship to study drug administration will be determined as follows:

- **Reasonable possibility**
  There are facts or arguments to suggest a causal relationship.

Cancers **2020**, *12*, x; doi: www.mdpi.com/journal/cancers

- **No reasonable possibility**
  Time relationship is improbable and another explanation is more likely (e.g. disease or other drugs provide plausible explanation).

13.2 Procedures for Adverse Event reporting

All Adverse Events

Toxicity will be monitored from the first study-related procedure (tTF-NGR application) until 8 days post last administration of tTF-NGR. All adverse events including serious adverse events that occur during this period will be recorded in the CRF.

Investigators must record in the CRF (Case Record Form) and the patient notes their opinion concerning details of nature, onset, duration/end, severity, seriousness, relationship to the IMP tTF-NGR and action taken concerning the IMP. Medical terminology should always be used to describe any event. Investigators should avoid vague terms such as “sick”. If possible, a diagnosis rather than a list of signs, symptoms and laboratory abnormalities should be given.

All SAEs will also be reported to the Sponsor using the Serious Adverse Event Form.

Serious adverse events (SAE) reporting

The investigator has to report any SAE occurring from the first study-related procedure (tTF-NGR application) until 8 days post last administration of tTF-NGR, including any compassionate use treatment after end of a patient’s study period. If the investigator suspects a reasonable causal relationship to the investigational medicinal product in an SAE occurring even later, this event should also be reported to the Safety Desk.

SAE reporting has to be done within 24 hours of knowledge by faxing the SAE form to the sponsor’s Safety Desk. Personal data have to be replaced by the trial patient number before forwarding any information.

Cancers **2020**, *12*, x; doi: www.mdpi.com/journal/cancers

Safety Desk Contact
Zentrum für Klinische Studien (ZKS) Münster<br>Von-Esmarch-Straße 62<br>48129 Münster<br>Germany<br>Fax: 0251 83 57112<br>Phone: 0251 83 57109<br>E-Mail: mssd@ukmuenster.de

Where possible, a diagnosis rather than a list of symptoms should be given. The investigator is responsible for assessing seriousness, severity (CTCAE v4.0) and causality of the SAE. The SAE form should be completed with as much information as possible. The investigator should not wait for full details before making the initial report.

Minimal information to be included in any initial report:

1. Trial patient number
2. Detailed description of the event
3. Details about administration of the IMP
4. Causality assessment of SAE to the IMP
5. Reporting person

The immediate reports should be followed promptly by detailed, written reports. In case of death, an autopsy report should be provided, if any. For reported deaths, the investigator should also supply the competent authority and the Ethics Committee with any information, if additionally requested by them.

All Serious Adverse Events will be followed up until resolution or definite outcome. The investigator will be asked to provide interim and follow-up reports, as necessary, if the SAE has not resolved at the time of initial report. The investigator should answer all queries from the Safety Desk as soon as possible.

Monitoring pregnancies for potential Serious Adverse Events

Pregnancy is not serious by itself. However, if a subject has a positive pregnancy test during trial participation, this is reportable on an SAE form in order to identify and follow-up on outcome of pregnancy and on any congenital abnormalities. The report

should be made as soon as the investigator gains knowledge of the event. Follow-up of a pregnancy will be done using specific additional questionnaires supplied by the Safety Desk if required. Information will be collected as far as covered by consent (information about the partner or about a child needs to be covered by consent of the patient's partner, too).

13.3 Sponsor's obligations

The Safety Desk will document each SAE, check it and query additionally required information.

Fulfilling sponsor's responsibilities, the Investigator will review each SAE again for seriousness and relatedness. Furthermore he will assess whether a serious adverse reaction is expected or unexpected (SUSAR) according to the current version of the IMPD, and whether any SAE might influence the benefit-risk-ratio or might require changes in the conduct of the trial.

It is the duty of the sponsor's Safety Desk to inform the Ethics Committee, the Competent Authority and participating investigators of all SUSARs in accordance with legal requirements (immediately, fatal or life-threatening SUSARs by the latest within 7 calender days, other SUSARs by the latest within 15 calender days of first knowledge. For fatal or life-threatening SUSARs, additional information will be provided as relevant within a further 8 days.). SUSAR follow-up reports will be submitted, if appropriate.

The Safety Desk will observe SUSAR cross reporting obligations with other trials of the same sponsor investigating the same active substance, if any.

The Investigator is responsible for the ongoing safety evaluation of the trial. The Safety Desk and the Investigator will inform each other immediately about any relevant safety information coming to their knowledge. In case of safety relevant issues (besides SUSARs) which require expedited reporting, the Safety Desk will support the Investigator in submitting an appropriate report in due time. This includes issues which might materially alter the current benefit-risk assessment of an investigational medicinal product or that would be sufficient to consider changes in the investigational medicinal product's administration or in the overall conduct of the trial as well as urgent safety measures to protect the subjects against any immediate hazard.

Annual safety reports will be prepared and submitted in accordance with legal requirements (Development Safety Update Report, DSUR). The Investigator is responsible for providing the updated benefit-risk assessment of the trial for the report (passages requiring medical assessment). The Safety Desk is responsible for preparing the other parts of the report, finalizing it and submitting it to the Competent Authority and the Ethics Committee in due time. The report will be prepared annually from the date of the initial clinical trial authorisation and submitted within 60 days of data lock point (day before anniversary of initial authorisation). Additional reports will be prepared on request by the Competent Authority or the Ethics Committee.

Details of all AEs will be reported to the Competent Authority on request.

14. Anticipated Risks for the Subjects

According to the first-in-man experience doses of tTF-NGR up to 4 mg/m^2 are tolerated without side effects when infused over a central venous access for 1 hour with weekly intervals. Single patients have been treated up to 5 times with weekly intervals. Anticipated side effects and toxicities are classified as those related to infusional reactions, venous thrombosis, arterial vascular occlusion and embolism, disseminated intravascular coagulation (DIC), tumor bleeding, antibody formation, liver toxicity, and a transient thrombocytopenia. A detailed justification of the starting dose for tTF-NGR in this trial is given in the IMPD.

14.1 Infusional reactions

Allergic reactions including rash, fever, rigors and bronchospasm are possible after infusion of proteins. This did not happen during the treatment of the first patients. However, in case these side effects occur, patients will be premedicated with chlorpheniramine, paracetamol and corticosteroids to prevent such reactions.

14.2 Venous thrombosis

The occurrence of venous thrombosis has not been observed in the patients treated with tTF-NGR. Additionally, such an event is unlikely, since there are rare binding sites for the NGR-binding moiety in venous endothelial cells of adult vessels. However, after repeated injections of the IMP into the tail veins of mice, acral necrosis occurred and high local concentrations might theoretically induce venous thrombosis. Thus, tTF-

Cancers **2020**, *12*, x; doi: www.mdpi.com/journal/cancers

NGR has to be infused over a time period of 1 hour via a central venous access. Patients have to be treated in-house with all appropriate drugs for fibrinolysis and anticoagulation ready to use.

14.3 Arterial vascular occlusion and embolism

The occurrence of arterial vascular occlusion and/embolism has not been observed in the patients treated with tTF-NGR. It also did not occur in mice treated within the therapeutic dose range. However, in the safety pharmacology studies with mice to establish limiting toxicity and maximum tolerated dose, we have observed pulmonary embolism and indirect signs of infarction in brain histology of mice deceasing upon treatment (see IMPD). Thus, patients have to be treated in-house with close back-up of an intensive care unit (ICU), their vital parameters (including ECG-monitoring, capillary O_2-saturation) and clinical symptoms have to be checked upon continuously during the infusion and for at least 5 hours afterwards by the investigator or a trained physician and in intervals later as specified by the protocol. Repeated checks on appropriate blood and serum parameters such as troponin and creatinine phosphokinase isoenzymes are outlined above. Appropriate drugs, including those for fibrinolysis and anticoagulation have to be ready for use.

14.4 DIC

DIC was not observed in the patients treated with tTF-NGR thus far. However, DIC can occur theoretically upon dose escalation. Thus patients have to be treated in-house as outlined above and have to be tested repeatedly for blood and serum parameters such as platelet count, D-dimer, fibrinogen, fibrin split products, plasmatic coagulation parameters as outlined above.

14.5 Tumor bleeding

During the preclinical studies we have observed thrombosed tumor vessels with blood pooling, disruption of vessels and intratumoral bleeding (see IMPD). This effect is part of the mechanism of action of tTF-NGR, which enriches and binds in tumor vessels and causes local thrombosis. During all animal studies we have observed sporadic deaths of treated mice with no clear dose-dependency. However, we had the impression this was occurring more often with animals carrying large tumors of >0.5 cm^3. Thus, tumor bleeding may occur. It may preferentially occur in patients with

Cancers **2020**, *12*, x; doi: www.mdpi.com/journal/cancers

large tumor masses. Thus, patients have to be treated in-house with appropriate transfusions of platelets and red blood cells available and prepared for.

14.6 Antibody formation

Human anti-human antibody (HAHA) formation is a theoretical concern although highly unlikely. 1. The amino acid sequence of the tTF-moiety is the unchanged human sequence and thus should be of low or absent immunogenicity. 2. The short NGR-binding moiety used has been reported as being of very low or absent immunogenicity (Di Matteo P et al. Immunogenic and structural properties of the Asn-Gly-Arg (NGR) tumor neovasculature-homing motif. Mol. Immunol. 43: 1508-1518, 2006). 3. The His-tag consists of less than 50 amino acids. However, antibody formation could occur after repeated application and possibly interfere with the therapeutic activity of tTF-NGR. Thus, patients with longer treatment duration will be monitored for HAHA formation.

14.7 Pregnancy, Breast Feeding and Fathering of a Child

There are no data on embryonal or fetal toxicity of tTF-NGR. Since endothelial cells in blood vessels of a growing fetus might express the targets CD13 and □lpha$_{v}$□eta$_{3}$, treatment of pregnant women has to be avoided. Premenopausal women are only allowed to participate in trials with the IMP under strict birth control and upon a negative result of a pregnancy test. Female patients are considered to have no child bearing potential if they are permanently sterile or post-menopausal. Permanent sterilization methods include hysterectomy, bilateral salpingectomy and bilateral oophorectomy. A postmenopausal state is defined as no menses for 12 months without alternative medical cause.

Female patients of childbearing potential (and male patients with a partner of child bearing potential) must agree to use a highly effective contraceptive method (Pearl index <1) throughout the study period and during the 2 months thereafter.

14.8 Liver toxicity

Since we have observed increase in bilirubin levels to values higher than the upper limit normal in the dog toxicology (see IMPD) liver toxicity including bilirubin values has to be monitored carefully throughout the study.

14.9 Transient thrombocytopenia

We have observed transient platelet drops under therapy with tTF-NGR in single patients before, which are possibly due to consumption of platelets within the tumor vascular infarction. Thus, this phenomenon might belong to the mechanism of action of the IMP. It is therefore necessary to treat the patients within this phase I study as "in-house" patients, to make fast transfusions of platelets realistic.

15. Assessment of disease status

Assessment of disease status is made at the time points given in the study calendars. Disease response criteria are according to the RECIST guidelines. The study radiologist will give special attention to tumor necrosis in lesions not shrinking in their outer diamenter and report these in addition. As a surrogate parameter for target hit, MRI will be performed. Vascular volume fraction will be measured to quantify reduction of blood flow in the tumor induced by tTF-NGR. These data will be reported in addition.

16. Statistical Considerations

All statistical evaluations within the study will be performed by Prof. Dr. J. Gerß.

16.1. Estimation of the number of patients needed to complete this trial.

The number of patients needed for completion of this trial is determined by the occurrence of dose limiting toxicity (DLT).

A first patient will be treated daily with tTF-NGR by 1-hour iv infusions for 5 days. Starting dose will be 1 mg/m^2/day, intraindividual dose-escalation will be performed in 0.5 mg/m^2/day steps upon a rest period of 16 days for the next treatment cycle of 5 days in this patient. If diagnosed with progressive disease (PD) after 2 cycles or clinically as judged by the investigator even before, this patient will go off study and will be replaced by a further patient in whom individual dose escalation will be resumed. Upon occurrence of DLT up to 5 further patients will be treated at this specific dose in a verification cohort. Further procedure such as de-escalation and definition of MTD and dose level recommended for further clinical trials will be performed as described above. Thus, a minimum number of patients to reach objectives of this study are envisaged as being 7 patients. This number of patients represent 1 patient for first

Cancers 2020, 12, x; doi: www.mdpi.com/journal/cancers

dose-escalation with DLT at the highest dose, starting the verification cohort and reproduction of DLT in the first patient (of up to 5 patients) of the verification cohort, de-escalation with -0.5 mg/m^2/day in 5 further patients with ≤1 additional DLT.

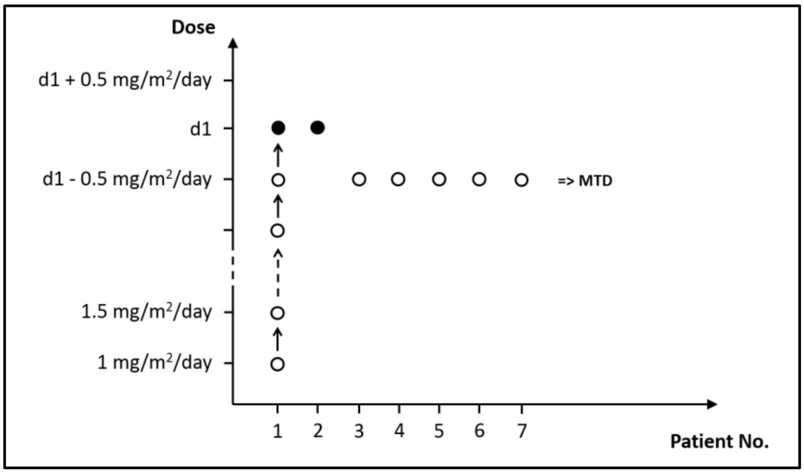

Possible higher numbers of patients in situations such as with no additional DLT in the verification group and further dose-escalation is possible, but cannot be determined before the study with confidence.

17. Study duration, Premature Termination of the Study

Planned start date of the study (FPFV, first patient first visit): 01.11.2016

Planned end date of recruitment (LPFV, last patient first visit): 01.11.2017

Planned end date of the study: 01.05.2018

Planned duration of single patient participation: according to occurrence of DLT and antitumor activity

Planned duration of recruitment: 01.11.2016 - 01.11.2017

Planned overall duration of the study: 01.11.2016 - 01.05.2018

Planned duration of follow up of individual patients within study: 3 months

Definition of the End of the Study

To allow reasonable time for evaluation and report, the end of study is plannned to be at "last patient last visit" (LPLV).

Premature Study Termination

Premature Termination of Study Participation of a Single Patient

A patient is free to withdraw consent and discontinue participation in the study at any time, without prejudice to further treatment according to standard clinical practice. In this case the patient will be asked to permit the conduct of the study termination visit.

In certain circumstances the investigator may exclude a patient from further study participation irrespective of the patient's will. Reasons may be:

- treatment failure
- insufficient compliance
- further participation might jeopardize the patient's health or well-being
- the patient was administered medication which is not permitted
- termination of the entire study.

If, after treatment initiation, a patient's participation is realized not to agree with the inclusion and exclusion criteria, the Investigator will decide on further participation.

In case of premature termination, the reason will be recorded on the CRF.

Premature Termination of the Entire Study

The sponsor may terminate the entire study for relevant medical or administrative reasons. Reasons for termination will be documented in detail. Particular reasons for termination of the study may be:

- insufficient recruitment
- serious problems with the quality of the study data which cannot be solved
- discovery of an unexpected, serious and unacceptable risk to patients enrolled in the study
- new scientific evidence suggesting that study continuation is not justified.

18. Informed Consent

As the study is performed in Muenster, Germany and is a monocentric study, the informed consent form will be provided in German as in Appendix 2.

Prior to inclusion into the study, the investigator informs each patient about nature, significance, implications, and risks of the study as well as about the patient's right to withdraw from study participation at any time without any resulting detriment. Further,

the patients are informed that an insurance has been taken out to cover risks originating from the study. According to the insurance conditions, they are advised to immediately inform the investigator if they were treated for an emergency and not to undergo any other medical treatment without prior consultation with the investigator.

Additionally, patients are handed out the patient information sheet incl. the informed consent form which are provided for this study. Patient consent in study participation must be given in writing. Before informed consent is requested, patients are left sufficient time for consideration. They are provided the opportunity for clarification of any study issues.

According to German drug law (“Arzneimittelgesetz”) §40(2a) patients are informed that their disease related data are stored in a pseudonymized way and used for scientific evaluations. For study participation they must agree therein in writing.

The informed consent form is dated and signed by the patient and by the investigator. The originally signed informed consent form is archived in the investigator site file. A copy of the signed informed consent form (or a second original) is handed over to the patient together with a copy of the patient information sheet and a copy of the general conditions of the patient insurance.

If the patient is unable to write, in exceptional cases, instead of the written consent required, oral consent in the presence of at least one witness, who was also included when the patient was being informed may be given. The witness may not be anyone working at the study site nor a member of the investigating team. The orally given consent has to be documented in writing, dated and signed by the witness.

In case of any study issue which requires a change of the patient information sheet, patients already included into the study must, if relevant to them, be informed about these issues orally and in writing and their written consent in further study participation must be obtained.

Note: Before undergoing any study specific procedure the patient must have provided written informed consent!

Cancers **2020**, *12*, x; doi: www.mdpi.com/journal/cancers

19. Study Administration

This study will be conducted in compliance with the protocol, the International Conference on Harmonisation of Good Clinical Practice (ICH GCP) guidelines and the Declaration of Helsinki (Fortaleza, 2013), the requirements of the current German drug law (“Arzneimittelgesetz”), the current legal provisions regarding data protection, and the principals of Good Clinical Practice.

The present study will not be started before the competent ethics committee has given a favorable opinion and an approval by the competent federal authority has been obtained.

In case of substantial amendments, a new application will be submitted to the ethics committee and/or the competent federal authority. Changes will not be implemented unless the competent ethics committee has given a favorable opinion and/or the competent authority has granted an approval.

Issues, which always require a favorable opinion of the ethics committee are for example:

- inclusion of additional study sites,
- change of the investigator or his deputy,
- changes in any documents addressed to study participants or in any study information addressed to potential study participants.

According to German drug law §67, the investigator is required to notify the local surveillance authority about the beginning, the end and a premature termination of the study, about any substantial amendments and if the study is on hold. These notifications will be performed by an appointee of the sponsor (GCP-V, §12(3)).
Additionally, the investigator is required to notify the competent federal authority of the beginning of the study.

19.1 Sponsor

This study is sponsored by the University Hospital Muenster (UKM). The study will be monitored by the Centre for Clinical Trials Münster of the UKM, the Zentrum für Klinische Studien (ZKS) Münster and performed according to the standard operating procedures of the ZKS Münster.

19.2 Monitoring

In order to ensure a high degree of data quality, the study site will be monitored during the recruitment and follow-up period (frequency depending on the site's recruitment).
The objectives of the monitoring procedures are to ensure that the study safety and rights of the study subjects as a study participant are respected and that accurate, valid and complete data are collected, and that the study is conducted in accordance with the study protocol, the principles of GCP and local legislation.
The investigator agrees that the monitor will visit the study site in appropriate intervals. During these visits the monitor will check the quality of the data recording and ensure that the study site adheres to the timeframe as set in the study protocol. The investigators agree to provide any relevant information and documentation whenever the monitor requires this information. This includes access to all original study documents and source data.

It is the responsibility of the investigator to keep the participant's chart as complete as possible (e.g. history, concomitant diseases, inclusion in the clinical study, visit dates, results of laboratory tests, distribution of the study medication, and adverse events). Source data are checked and compared with entries in the eCRF. The participant has given consent with this procedure by signing the patient information and written informed consent form. Additional tasks of the monitor are:

- to check, whether the study site fulfils requirements of the clinical study (e.g. participant population, technical equipment),
- instruction of the investigators and personnel for the clinical study,
- to check the ISF for completeness and actuality,
- documentation of the status of the participant,
- matching of original data,
- to check SAE reports according to regulations.

The monitor has the responsibility to treat all information confidentially and to safeguard the integrity and personal privacy of the study participants.

The exact extent of the monitoring procedures is described in a separate monitoring manual.

Cancers **2020**, *12*, x; doi: www.mdpi.com/journal/cancers

19.3 Audits und Inspections

For the purpose of ensuring compliance with the study protocol, the principles of GCP and local legislation, the sponsor may initiate an audit at any study site during the study and after completion. All study-related documentation must be made available to the designated auditor(s).

In addition, representatives of the regulatory authorities may choose to inspect a study site at any time prior to, during, or after completion of the clinical study. In this case, all pertinent study data should be made available as requested to the regulatory authority for verification, or inspection purposes. The investigator has to be available during these visits.

19.4 Documentation, Data Management, Archiving

Patient Identification List

All subject data will be collected in a pseudonymized form. Every trial subject can be identified by a unique subject identification code consisting of a three digit center code, a hyphen and three digit numbers. A confidential subject identification list which links the patients' names with the subject identification code will be stored in the investigator site file.

Source Data / Source documents

Source data are, within the meaning of the ICH E6 Guideline, all information in original records and certified copies of original records of clinical findings, observations, or other activities in a clinical trial necessary for the reconstruction and evaluation of the trial. Source data will be documented in various source documents (e.g. hospital records, doctor's report, subjects' diaries or evaluation checklists, x-rays) and then entered into the electronic Case Report Form (eCRF).

The following study specific data will only be recorded electronically in separate files and is not going to be entered in the eCRF*:*

- pharmacokinetics

Recording of Data / Case Report Form (CRF)

Data will be recorded electronically using an EDC (Electronic Data Capture) system. Only persons authorised to enter data into the eCRF will have access to the EDC system. All users will be trained to use the EDC system and will comply with the instructions in the study-specific user manual. They will have continuous access to the data and reports of subjects at their own study site. The investigator is responsible for ensuring that the study data will be documented correctly, completely and in a timely manner. A study team physician takes on responsibility for the collected data by signing electronically. The electronic signature according to FDA 21 CFR Part 11 is the legally binding equivalent of the study team physician's handwritten signature.

Data Management

For data management, the validated data management system MACRO v4 (Elsevier InferMed) will be used. All entered data will be stored on servers of the Universitätsklinikum Münster. The servers are located in a secure data center and behind a firewall in the network of the Universitätsklinikum Münster. A backup of the data will be saved on a daily basis and all data changes will be recorded in an audit trail.

All data will be checked for plausibility during initial data entry. Missing or non-plausible data are highlighted by the system right at input at the clinical study site and may be corrected immediately. Thereafter, according to the data validation plan, further data checks will be performed with regard to completeness and plausibility by the data management of the ZKS Münster. In case of non-plausible or missing data, queries will be sent to the study site. The queries must be resolved by authorized members of the investigator`s staff in the respective study site in a timely manner.

After completion of data entry and data processing, the database will be locked and the data will be exported for statistical analysis. The investigator will receive a CD-ROM of the eCRF data for archiving at the clinical study site.

Archiving

After the end of the trial, the originals of all trial-specific documents (Trial Master File) including the CRFs must be stored by the sponsor according to national regulations (§ 13(10) GCP-Verordnung) for at least 10 years. Furthermore, the investigator stores the ISF (Investigator Site File) including copies of the CRFs for the time period given above.

Trial data or documents may not be destroyed without prior written agreement between the sponsor and the investigator(s) or his/her designee.

Investigator Site File

Each study site keeps an ISF provided by the investigator/sponsor. During monitoring, the ISF will be checked regularly for completeness and actuality. After the clinical trial is finished or stopped, the ISF has to be stored for at least 10 years.

20. Patient Insurance

As required by German drug law, patients will be insured against risks originating from the study.

Insurance: HDI-Gerling Industrie Versicherung AG
Riethorst 2
30659 Hannover.

21. Financing

The study is financed by Deutsche Krebshilfe, grant 111004 to Univ.-Prof. Dr. Wolfgang E. Berdel.

22. Adherence to the Protocol

The investigator must adhere to the protocol as detailed in this document. Substantial changes to the protocol will require a written favorable opinion from the competent ethics committee and written approval by the competent authority prior to implementation. This does not apply when the modification is needed to eliminate an immediate hazard to patients. Any deviations from the protocol must be fully

documented in the source documentation and recorded and explained in the CRF (if applicable).

23. Data Protection

This study will be performed in compliance with the applicable data protection laws. Study personnel will handle all patient data in a strictly confidential way.

To prevent the identification of a person to whom study data belong, study data will be pseudonymized by means of the patient identification number. If patient documents (e.g., examination results) are transferred to an institution outside the study site, copies will be used on which the patient's name and initials are obscured and the patient identification number is indicated.

24. Reporting and Publication Policy

Final Report

After completion of the biometric evaluation, the Investigator prepares a study report. The report includes all trial results, irrespective of whether favorable or not. It is signed by him and the person who is responsible for the evaluation.

Within 12 months after the end of the study, the Investigator submits a summary of the final report to the ethics committee and to the competent federal authority. The summary is prepared in the format provided for report synopses by the ICH Guideline E3 "Guidance on Structure and Content of Clinical Study Reports". After review by the competent federal authority the report is published on the web site of DIMDI (German drug law, §42 b).

According to European Commission Guideline 2012/C 302/03 the study results should be posted within 12 months to the EU Database on Clinical Trials (EudraCT).

Safety Reports

The rules for the annual safety reports are specified above.

Cancers **2020**, *12*, x; doi: www.mdpi.com/journal/cancers

Publication Policy

The study will be registered in a clinical trials database, which is accessible to the public.

Publication details, authorship and journal selection will be decided by the Investigator and Prof. Berdel.

APPENDIX

Cancers **2020**, *12*, x; doi: www.mdpi.com/journal/cancers
