# Supplementary material for: First-In-Class CD13-Targeted Tissue Factor tTF-NGR in Patients with Recurrent or Refractory Malignant Tumors: Results of a Phase I Dose-Escalation Study
Source: Cancers (Basel). 2020 Jun 7;12(6):1488. doi: 10.3390/cancers12061488 (PMC7352358; doi:10.3390/cancers12061488)
Supplement: Supplementary file 1 [file cancers-12-01488-s001.zip › cancers-841723_supplementary_for_XMLberdel corr.pdf]

cancers

Supplementary Material

First-in-Class CD13-Targeted Tissue Factor tTF-NGR in Patients with Recurrent or Refractory Malignant Tumors Beyond Standard Treatment: Results of a Phase I Dose-Escalation Study

Christoph Schliemann [1], Mirjam Gerwing, Hauke Heinzow, Saliha Harrach, Christian Schwöppe, Moritz Wildgruber, Anna A. Hansmeier, Linus Angenendt, Andrew F. Berdel, Ursula Stalmann, Björna Berning, Karsten Kratz-Albers, Kristina Middelberg-Bisping, Stefanie Wiebe, Jörn Albring, Christian Wilms, Wolfgang Hartmann, Eva Wardelmann, Tobias Krähling, Walter Heindel, Joachim Gerss, Eike Bormann, Hartmut Schmidt, Georg Lenz, Torsten Kessler, Rolf M. Mesters, Wolfgang E. Berdel [1]

[1] Department of Medicine A (Hematology and Oncology), University Hospital Muenster, Albert Schweitzer Campus 1, D-48149 Muenster, Germany; email: Christoph.Schliemann@ukmuenster.de, berdel@uni-muenster.de

CONTENT

Supplementary Figures p. 2
Supplementary Tables p. 9

Supplementary Figures

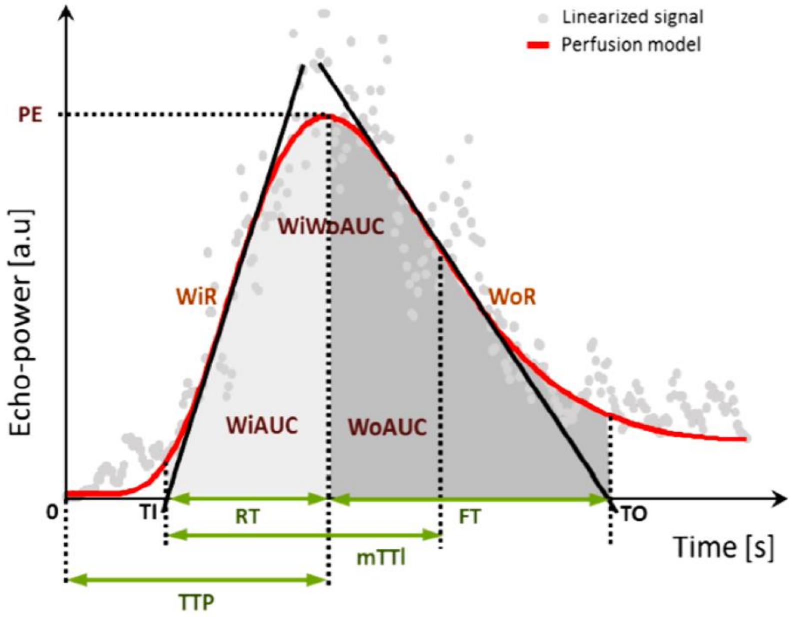

Abbreviations	Time-intensity curve parameters on CEUS	Units
**PE**	Peak-Enhancement	[a.u.]
**WiAUC**	Wash-in Area Under the Curve *(AUC (TI:TTP))*	[a.u.]
**RT**	Rise Time – Anstiegszeit *(TTP – TI)*	[s]
**mTTI**	Mean Transit Time local – mittlere Durchgangszeit (local) *(mTT – TI)*	[s]
**TTP**	Time to Peak	[s]
**TO**	Tangent Out (maximum slope intersection with x-axis)	[s]
**WiR**	Wash-in-Rate *(maximaler Anstieg)*	[a.u.]
**WIPI**	Wash-in Perfusion Index – Wash-inPerfusionsindex *(WiAUC / RT)*	[a.u.]
**WoAUC**	Wash-out AUC *(AUC (TTP:TO))*	[a.u.]
**WiWoAUC**	Wash-in- and Wash-out-AUC *(WiAUC + WoAUC)*	[a.u.]
**FT**	Fall Time – Abstiegszeit *(TO-TTP)*	[s]
**WoR**	Wash-out-Rate (geringster Anstieg)	[a.u.]
**QOF**	Quality Of Fit between the echo-power signal and f(t) - Passungsqualität zwischen dem Echointensitätssignal und f(t)	[%]

Figure S1. Dynamic contrast-enhanced ultrasound (CEUS). Schematic drawing of time-intensity curve parameters of a perfusion model in contrast-enhanced ultrasound (CEUS) imaging. The figure was modified with consent from the manufacturer Bracco Imaging Deutschland GmbH, the property and the copy right remains with Bracco.

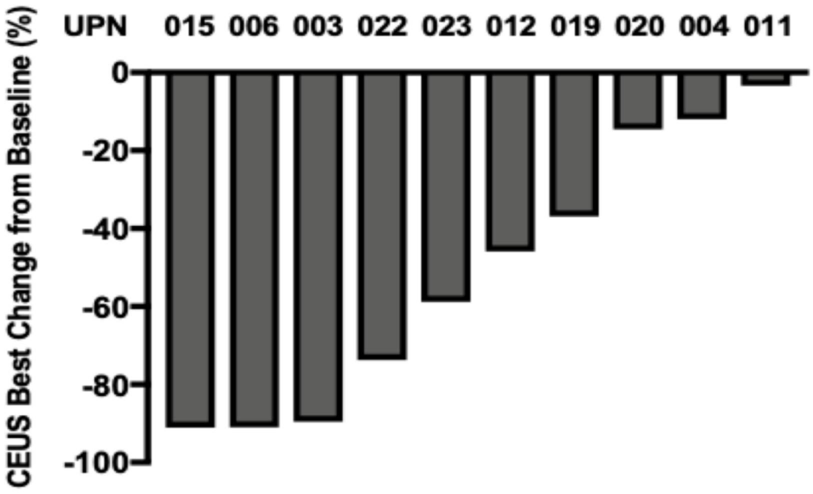

Figure S2. CEUS blood flow decrease in all patients with measurable lesions. Waterfall plots of UPNs with completed cycle 1 show best change from baseline of tumor perfusion within cycle 1 per patient.

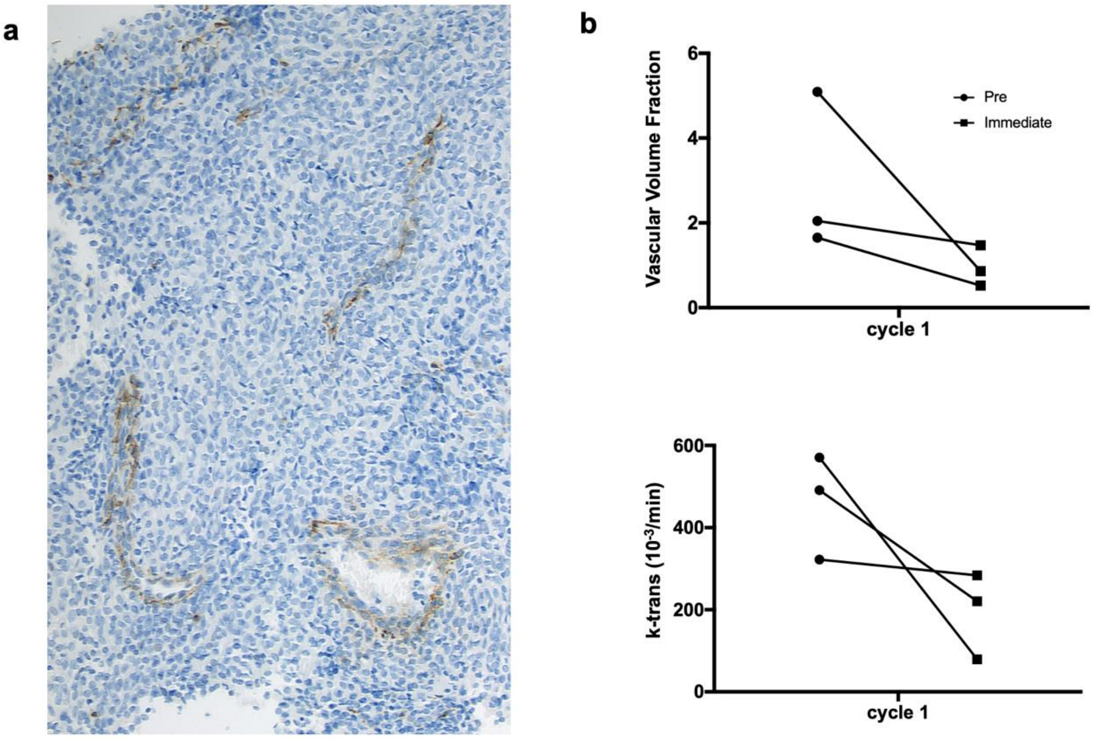

Figure S3. Reduction of tumor perfusion by tTF-NGR in a synovial sarcoma with CD13 positivity in the tumor vasculature only. (**a**) Immunohistochemistry of a synovial sarcoma (UPN 008) showing CD13 negativity of the tumor cells, but CD13 positivity (brownish stain, intensity score 2; ref. Kessler et al., manuscript text ref. 20) in the tumor vasculature. (**b**) Tumor blood flow in 3 different lesions of liver metastasis in the same patient as measured by MRI depicting k-trans and VVF before tTF-NGR application (Pre) and 5 hours after (Immediate) the 1st application of tTF-NGR.

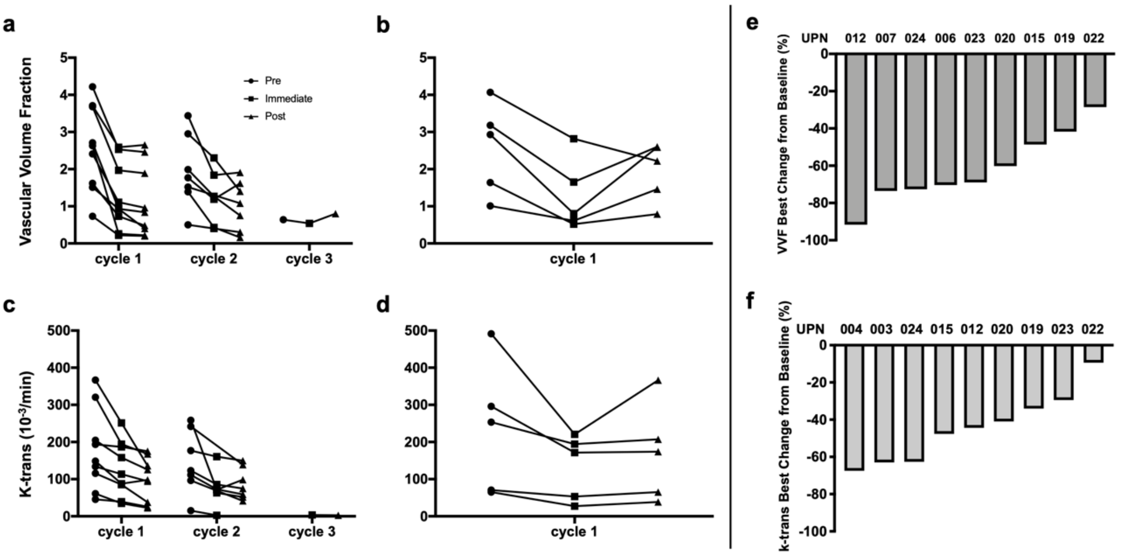

Figure S4. Changes in tumor perfusion during treatment with tTF-NGR in all patients serially imaged. Changes of MRI perfusion parameters before (Pre), during (Immediate, after 5 hours) and after treatment (Post, after 5 days at the end of a cycle) of completed cycles are shown in the left panels for Vascular Volume Fraction VVF (**a**; UPN 007, 012, 015, 019, 020, 022, 023, 024) and K-trans (**c**; UPN 003, 004, 012, 015, 019, 020, 022, 023, 024). VVF (**a, b**; arbitrary units in the y-axis) and k-trans (**c, d**; in 10^{-3}/min) both show a decrease in perfusion after treatment with tTF-NGR in all patients. Incomplete cycles due to a rise in Troponin T hs (DLT) are shown in the right panels for VVF (**b**; UPN 008, 009, 011, 018, 021) and K-trans (**d**; UPN 008, 009, 011, 018, 021). Notice that immediate decrease of tumor perfusion (5-hour values, black squares) in all patients (**b, d**) was blocked and/or reversed (Post) by stop of tTF-NGR application and initiation of anticoagulation further demonstrating the mode of action of tTF-NGR. Waterfall plots of UPNs with completed cycle 1 (**e**; VVF and **f**; k-trans) show best change from baseline of tumor perfusion within cycle 1 per patient.

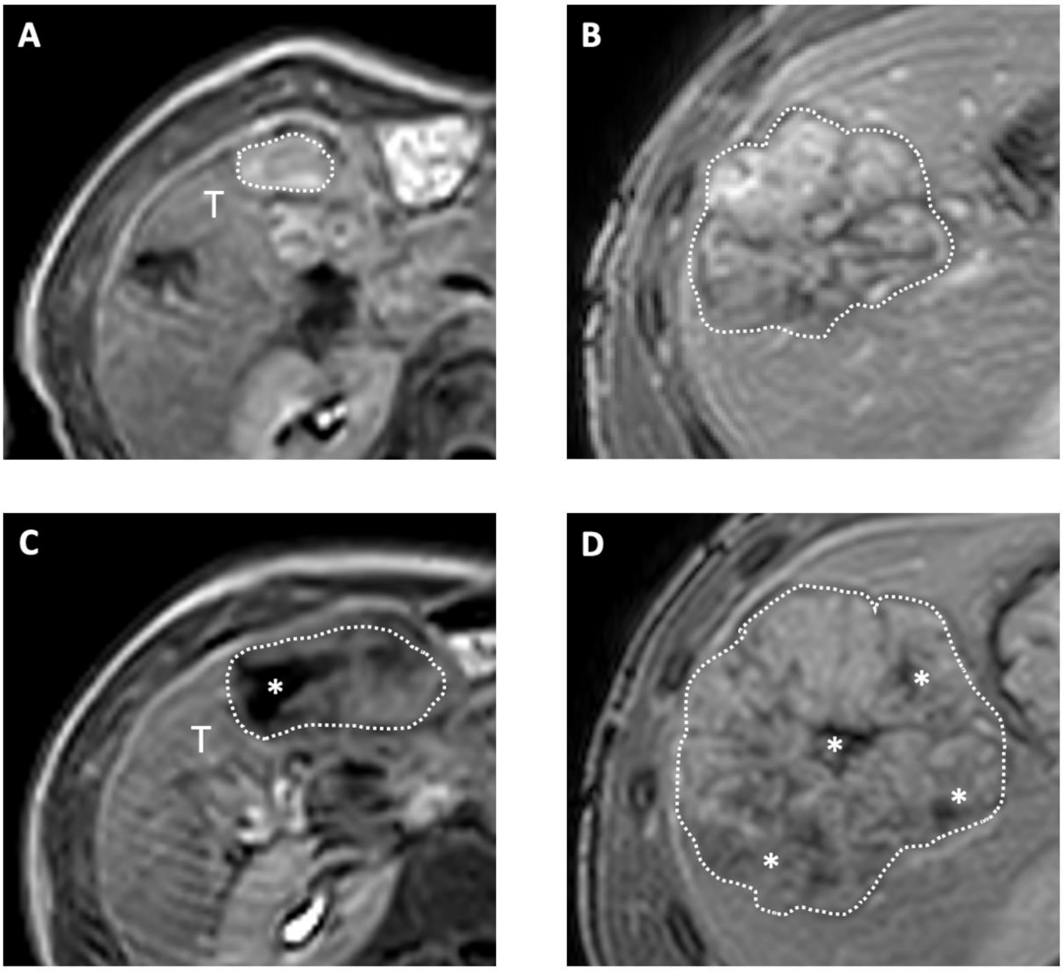

Figure S5. Intratumoral necrosis in liver metastases of a germ cell tumor (UPN 003). *A*/*B*: Before treatment with tTF-NGR at 1.5 mg/m^2 × 5, the tumor (dotted line) shows a more homogenous contrast enhancement in T1 weighted MRI. *C*/*D:* After two cycles of tTF-NGR, hypointense areas without contrast enhancement (*) are visible under progressive disease.

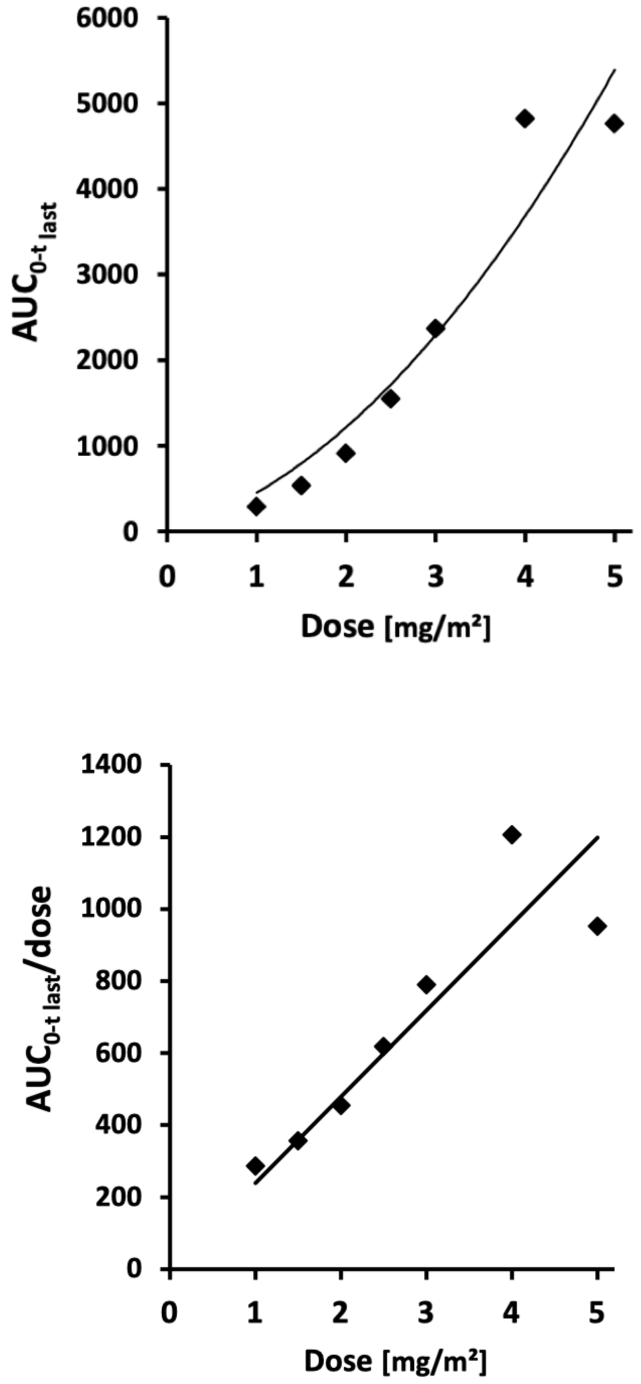

Figure S6. Visualization of the dose proportionality factor (DPF). Two different calculations are shown. Values at 5 mg/m² tTF-NGR have to be interpreted with caution since this dose level was complicated by difficulties to draw and prepare blood samples for testing due to sticky consistency.

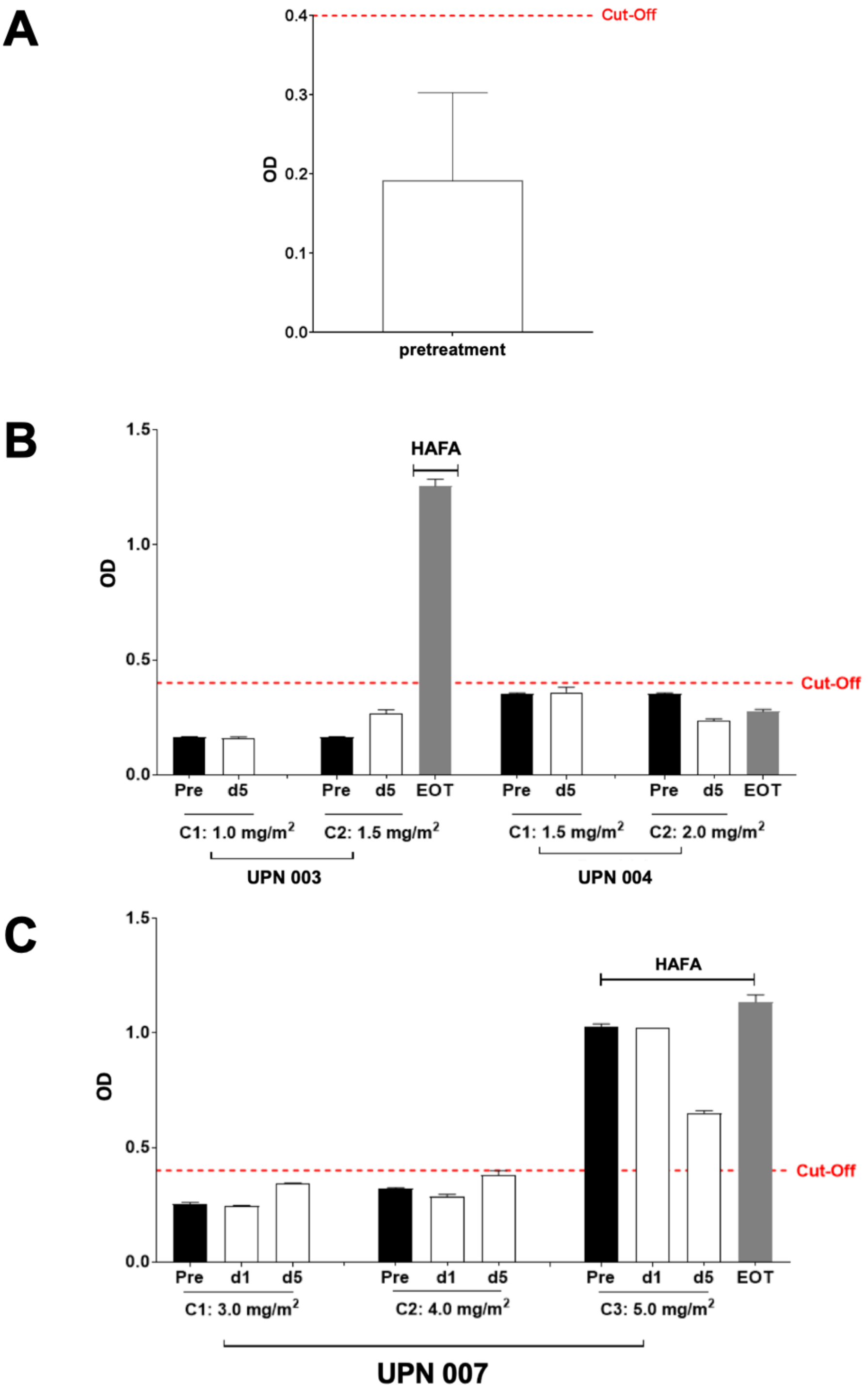

Figure S7. Detection of human anti-fusion protein (tTF-NGR) IgG antibodies (HAFA) in the patient´s plasma. (**A**) Establishment of the cut-off value on the basis of all pretreatment values (mean +/- standard deviation). (**B**) Patient UPN 004 shows values typical for the 15 patients without HAFA formation. Patient UPN 007 showed clearly detectable but low HAFA titers in the 3rd cycle of therapy. OD, optical density; C, cycle; d, day; Pre, before treatment; EOT, end of treatment (for details see Methods).

Supplementary Tables

Table S1. AE listing.

Page 1 of 8

tTF-NGR Phase I Study EudraCT-No.: 2016-003042-85

Line listing of adverse events (AEs) as reported in eCRF at 16.12.2019

Patient ID	AE No.	MedDRA preferred term (v22.1)	Date of onset of AE	Date of end of AE	or ongoing	CTCAE Grading (v 4.0)	serious (=SAE)	Relationship to tTF-NGR	Action taken with tTF-NGR	Comment on action taken with tTF-NGR	AE considered as DLT by investi-gator	AE that caused dis-continuation of whole study	Outcome
057-003	1	Cough	01/06/2017	09/06/2017	.	Grade 1 (mild)	No	No reasonable possibility	Dose not changed		No	No	Recovered/ Resolved
057-003	2	Fatigue	03/06/2017	04/06/2017	.	Grade 1 (mild)	No	No reasonable possibility	Dose not changed		No	No	Recovered/ Resolved
057-003	3	Aspartate aminotransferase increased	08/05/2017	08/05/2017	.	Grade 1 (mild)	No	No reasonable possibility	Dose not changed		No	No	Recovered/ Resolved
057-003	4	Aspartate aminotransferase increased	24/05/2017	05/07/2017	.	Grade 1 (mild)	No	No reasonable possibility	Dose not changed		No	No	Recovered/ Resolved
057-003	5	Alanine aminotransferase increased	24/05/2017	24/05/2017	.	Grade 1 (mild)	No	No reasonable possibility	Dose not changed		No	No	Recovered/ Resolved
057-003	6	Alanine aminotransferase increased	29/05/2017	05/07/2017	.	Grade 1 (mild)	No	No reasonable possibility	Dose not changed		No	No	Recovered/ Resolved
057-003	7	Blood alkaline phosphatase increased	24/05/2017	10/12/2017	.	Grade 1 (mild)	No	No reasonable possibility	Dose not changed		No	No	Not recovered/ Not resolved
057-003	8	Blood lactate dehydrogenase increased	24/05/2017	10/12/2017	.	Grade 2 (moderate)	No	No reasonable possibility	Dose not changed		No	No	Not recovered/ Not resolved
057-003	9	Thrombocytopenia	08/05/2017	09/05/2017	.	Grade 1 (mild)	No	No reasonable possibility	Dose not changed		No	No	Recovered/ Resolved
057-003	10	International normalised ratio increased	08/05/2017	08/05/2017	.	Grade 1 (mild)	No	No reasonable possibility	Dose not changed		No	No	Recovered/ Resolved
057-003	11	International normalised ratio increased	05/05/2017	05/07/2017	.	Grade 1 (mild)	No	No reasonable possibility	Dose not changed		No	No	Recovered/ Resolved
057-003	12	Activated partial thromboplastin time prolonged	08/05/2017	08/05/2017	.	Grade 1 (mild)	No	No reasonable possibility	Dose not changed		No	No	Recovered/ Resolved
057-003	13	Alpha 1 foetoprotein increased	29/05/2017	10/12/2017	.	Grade 3 (severe)	No	No reasonable possibility	Dose not changed		No	No	Not recovered/ Not resolved

ZKS Münster SAS-program: tTF_NGR_AE_Listing_V1.3.sas Date of export: 16.12.2019

Page 2 of 8

tF-NGR Phase I Study EudraCT-No.: 2016-003042-85

Line listing of adverse events (AEs) as reported in eCRF at 16.12.2019

Patient ID	AE No.	MedDRA preferred term (v22.1)	Date of onset of AE	Date of end of AE	or ongoing	CTCAE Grading (v 4.0)	serious (=SAE)	Relationship to tTF-NGR	Action taken with tTF-NGR	Comment on action taken with tTF-NGR	AE considered as DLT by investi-gator	AE that caused dis-continuation of whole study	Outcome
057-003	14	Blood cholinesterase decreased	01/06/2017	01/06/2017	.	Grade 1 (mild)	No	No reasonable possibility	Dose not changed		No	No	Recovered/ Resolved
057-003	15	Plasmin inhibitor decreased	08/05/2017	08/05/2017	.	Grade 1 (mild)	No	Reasonable possibility	Dose not changed		No	No	Recovered/ Resolved
057-003	16	Fibrin D dimer increased	08/05/2017	08/05/2017	.	Grade 1 (mild)	No	Reasonable possibility	Dose not changed		No	No	Recovered/ Resolved
057-003	17	Antithrombin III decreased	08/05/2017	08/05/2017	.	Grade 1 (mild)	No	Reasonable possibility	Dose not changed		No	No	Recovered/ Resolved
057-004	1	Nausea	14/06/2017	15/06/2017	.	Grade 1 (mild)	No	No reasonable possibility	Dose not changed		No	No	Recovered/ Resolved
057-004	2	Headache	15/06/2017	15/06/2017	.	Grade 1 (mild)	No	No reasonable possibility	Dose not changed		No	No	Recovered/ Resolved
057-004	3	Pruritus	15/06/2017	16/06/2017	.	Grade 1 (mild)	No	No reasonable possibility	Dose not changed		No	No	Recovered/ Resolved
057-004	4	Blood bilirubin increased	20/06/2017	24/06/2017	.	Grade 4 (lifethreatening/ disabling)	No	No reasonable possibility	Dose not changed		No	No	Recovered/ Resolved
057-004	5	Pruritus	27/06/2017	31/07/2017	.	Grade 1 (mild)	No	No reasonable possibility	Dose not changed		No	No	Not recovered/ Not resolved
057-004	6	Hypokalaemia	03/07/2017	04/07/2017	.	Grade 1 (mild)	No	No reasonable possibility	Dose not changed		No	No	Recovered/ Resolved
057-004	7	Anaemia	05/07/2017	06/07/2017	.	Grade 3 (severe)	No	No reasonable possibility	Dose not changed		No	No	Recovered/ Resolved
057-004	8	Supraventricular tachycardia	04/07/2017	04/07/2017	.	Grade 3 (severe)	No	No reasonable possibility	Dose not changed		No	No	Recovered/ Resolved
057-004	9	General physical health deterioration	03/07/2017	31/07/2017	.	Grade 3 (severe)	No	No reasonable possibility	Dose not changed		No	No	Not recovered/ Not resolved
057-004	10	Nausea	03/07/2017	05/07/2017	.	Grade 1 (mild)	No	No reasonable possibility	Dose not changed		No	No	Recovered/ Resolved

ZKS Münster SAS-program: tTF_NGR_AE_Listing_V1.3.sas Date of export: 16.12.2019

tTF-NGR Phase I Study EudraCT-No.: 2016-003042-85

Line listing of adverse events (AEs) as reported in eCRF at 16.12.2019

Patient ID	AE No.	MedDRA preferred term (v22.1)	Date of onset of AE	Date of end of AE	or ongoing	CTCAE Grading (v 4.0)	serious (=SAE)	Relationship to tTF-NGR	Action taken with tTF-NGR	Comment on action taken with tTF-NGR	AE considered as DLT by investi-gator	AE that caused dis-continuation of whole study	Outcome
057-004	11	Nausea	23/07/2017	23/07/2017	.	Grade 1 (mild)	No	No reasonable possibility	Dose not changed		No	No	Recovered/ Resolved
057-005	1	Constipation	24/07/2017	25/07/2017	.	Grade 1 (mild)	No	No reasonable possibility	Dose not changed		No	No	Recovered/ Resolved
057-005	2	Pain in extremity	24/07/2017	25/07/2017	.	Grade 2 (moderate)	No	No reasonable possibility	Dose not changed		No	No	Recovered/ Resolved
057-005	3	Cough	25/07/2017	30/08/2017	.	Grade 2 (moderate)	No	No reasonable possibility	Dose not changed		No	No	Recovered/ Resolved
057-005	4	Bacterial infection; Device related infection	25/07/2017	26/07/2017	.	Grade 2 (moderate)	No	No reasonable possibility	Dose not changed		No	No	Recovered/ Resolved
057-005	5	Back pain	25/07/2017	.	Ongoing	Grade 2 (moderate)	No	No reasonable possibility	Dose not changed		No	No	Recovering/ Resolving
057-005	6	Anaemia	25/07/2017	25/07/2017	.	Grade 3 (severe)	No	No reasonable possibility	Dose not changed		No	No	Recovered/ Resolved
057-005	7	Haemoptysis	04/08/2017	05/08/2017	.	Grade 3 (severe)	No	No reasonable possibility	Dose not changed		No	No	Recovered/ Resolved
057-005	8	Haemoptysis	15/08/2017	16/08/2017	.	Grade 2 (moderate)	No	No reasonable possibility	Dose not changed		No	No	Recovered/ Resolved
057-005	9	Nausea	14/08/2017	18/08/2017	.	Grade 1 (mild)	No	Reasonable possibility	Dose not changed		No	No	Recovered/ Resolved
057-005	10	Vertigo	15/08/2017	16/08/2017	.	Grade 1 (mild)	No	Reasonable possibility	Dose not changed		No	No	Recovered/ Resolved
057-005	11	Headache	15/08/2017	18/08/2017	.	Grade 1 (mild)	No	Reasonable possibility	Dose not changed		No	No	Recovered/ Resolved
057-005	12	Troponin T increased	16/08/2017	30/08/2017	.	Grade 1 (mild)	No	Reasonable possibility	Dose not changed		No	No	Recovered/ Resolved
057-005	13	Pyrexia	18/08/2017	18/08/2017	.	Grade 1 (mild)	Yes	No reasonable possibility	Dose not changed		No	No	Recovered/ Resolved
057-006	1	Abdominal pain	04/09/2017	15/09/2017	.	Grade 1 (mild)	No	Reasonable possibility	Dose not changed		No	No	Not recovered/ Not resolved

tTF-NGR Phase I Study EudraCT-No.: 2016-003042-85

Line listing of adverse events (AEs) as reported in eCRF at 16.12.2019

Patient ID	AE No.	MedDRA preferred term (v22.1)	Date of onset of AE	Date of end of AE	or ongoing	CTCAE Grading (v 4.0)	serious (=SAE)	Relationship to tTF-NGR	Action taken with tTF-NGR	Comment on action taken with tTF-NGR	AE considered as DLT by investi-gator	AE that caused dis-continuation of whole study	Outcome
057-006	2	Abdominal pain	15/09/2017	29/09/2017	.	Grade 3 (severe)	Yes	No reasonable possibility	Dose not changed		No	No	Not recovered/ Not resolved
057-006	3	Back pain; Musculoskeletal pain; Arthralgia	04/09/2017	14/09/2017	.	Grade 2 (moderate)	No	No reasonable possibility	Dose not changed		No	No	Recovered/ Resolved
057-006	4	Decreased appetite	16/09/2017	18/09/2017	.	Grade 1 (mild)	No	No reasonable possibility	Dose not changed		No	No	Recovered/ Resolved
057-006	5	Nausea	16/09/2017	18/09/2017	.	Grade 2 (moderate)	No	No reasonable possibility	Dose not changed		No	No	Recovered/ Resolved
057-006	6	Urinary tract infection	07/09/2017	08/09/2017	.	Grade 2 (moderate)	No	No reasonable possibility	Dose not changed		No	No	Recovered/ Resolved
057-006	7	Pleural effusion	11/10/2017	18/10/2017	.	Grade 3 (severe)	No	No reasonable possibility	Not applicable		No	No	Recovered/ Resolved
057-006	8	Oedema peripheral	21/10/2017	23/10/2017	.	Grade 2 (moderate)	No	No reasonable possibility	Not applicable		No	No	Recovered/ Resolved
057-007	1	Nausea	04/12/2017	22/01/2018	.	Grade 2 (moderate)	No	No reasonable possibility	Dose not changed		No	No	Recovered/ Resolved
057-007	2	Pyrexia	28/12/2017	29/12/2017	.	Grade 1 (mild)	No	Reasonable possibility	Dose not changed		No	No	Recovered/ Resolved
057-007	3	Pyrexia	15/01/2018	15/01/2018	.	Grade 1 (mild)	No	Reasonable possibility	Dose not changed		No	No	Recovered/ Resolved
057-007	4	Vomiting	27/12/2017	27/12/2017	.	Grade 1 (mild)	No	No reasonable possibility	Dose not changed		No	No	Recovered/ Resolved
057-007	5	Bone pain	27/12/2017	27/12/2017	.	Grade 1 (mild)	No	No reasonable possibility	Dose not changed		No	No	Recovered/ Resolved
057-007	6	Vertigo	30/12/2017	30/12/2017	.	Grade 1 (mild)	No	No reasonable possibility	Dose not changed		No	No	Recovered/ Resolved
057-007	7	Cough	26/12/2017	26/12/2017	.	Grade 1 (mild)	No	No reasonable possibility	Dose not changed		No	No	Recovered/ Resolved
057-007	8	Haematuria	06/12/2017	07/12/2017	.	Grade 1 (mild)	No	No reasonable possibility	Dose not changed		No	No	Recovered/ Resolved

tTF-NGR Phase I Study EudraCT-No.: 2016-003042-85

Line listing of adverse events (AEs) as reported in eCRF at 16.12.2019

Patient ID	AE No.	MedDRA preferred term (v22.1)	Date of onset of AE	Date of end of AE	or ongoing	CTCAE Grading (v 4.0)	serious (=SAE)	Relationship to tTF-NGR	Action taken with tTF-NGR	Comment on action taken with tTF-NGR	AE considered as DLT by investi-gator	AE that caused dis-continuation of whole study	Outcome
057-007	9	Headache	06/12/2017	07/12/2017	.	Grade 1 (mild)	No	No reasonable possibility	Dose not changed		No	No	Recovered/ Resolved
057-007	10	Chest pain	01/12/2017	05/12/2017	.	Grade 1 (mild)	No	No reasonable possibility	Not applicable		No	No	Recovered/ Resolved
057-008	1	Headache	19/02/2018	20/02/2018	.	Grade 1 (mild)	No	No reasonable possibility	Dose not changed		No	No	Recovered/ Resolved
057-008	2	Pain in extremity	20/02/2018	20/02/2018	.	Grade 1 (mild)	No	Reasonable possibility	Dose not changed		No	No	Recovered/ Resolved
057-008	3	Deep vein thrombosis	20/02/2018	22/02/2018	.	Grade 2 (moderate)	Yes	Reasonable possibility	Drug permanently withdrawn	study Medication stopped for this patient	No	No	Recovered/ Resolved
057-008	4	Troponin T increased	21/02/2018	02/03/2018	.	Grade 3 (severe)	Yes	Reasonable possibility	Drug permanently withdrawn	study Medication stopped for this patient	Yes	No	Recovered/ Resolved
057-008	5	Nasopharyngitis	20/03/2018	21/03/2018	.	Grade 1 (mild)	No	No reasonable possibility	Not applicable		No	No	Recovered/ Resolved
057-009	1	Hypokalaemia	05/03/2018	05/03/2018	.	Grade 1 (mild)	No	Reasonable possibility	Dose not changed		No	No	Recovered/ Resolved
057-009	2	Troponin T increased	05/03/2018	21/03/2018	.	Grade 3 (severe)	Yes	Reasonable possibility	Drug permanently withdrawn	study Medication stopped for this patient	Yes	No	Recovered/ Resolved
057-009	3	Back pain	07/03/2018	09/03/2018	.	Grade 1 (mild)	No	No reasonable possibility	Dose not changed		No	No	Recovered/ Resolved
057-011	1	Troponin T increased	25/06/2018	.	Ongoing	Grade 1 (mild)	No	No reasonable possibility	Dose not changed		No	No	Not recovered/ Not resolved
057-011	2	Troponin T increased	26/06/2018	09/07/2018	.	Grade 3 (severe)	Yes	Reasonable possibility	Drug permanently withdrawn		Yes	No	Recovering/ Resolving
057-011	3	Blood lactic acid increased	04/07/2018	05/07/2018	.	Grade 2 (moderate)	No	No reasonable possibility	Dose not changed		No	No	Recovered/ Resolved

Page 6 of 8

tTF-NGR Phase I Study EudraCT-No.: 2016-003042-85

Line listing of adverse events (AEs) as reported in eCRF at 16.12.2019

Patient ID	AE No.	MedDRA preferred term (v22.1)	Date of onset of AE	Date of end of AE	or ongoing	CTCAE Grading (v 4.0)	serious (=SAE)	Relationship to tTF-NGR	Action taken with tTF-NGR	Comment on action taken with tTF-NGR	AE considered as DLT by investi-gator	AE that caused dis-continuation of whole study	Outcome
057-011	4	N-terminal prohormone brain natriuretic peptide increased	04/07/2018	04/07/2018	.	Grade 2 (moderate)	No	Reasonable possibility	Drug permanently withdrawn		No	No	Recovered/ Resolved
057-012	1	Anaemia	10/07/2018	10/07/2018	.	Grade 3 (severe)	No	No reasonable possibility	Dose not changed		No	No	Recovered/ Resolved
057-012	2	Pyrexia	09/07/2018	11/07/2018	.	Grade 1 (mild)	No	No reasonable possibility	Dose not changed		No	No	Recovered/ Resolved
057-012	3	Abdominal pain upper; Tumour pain	15/07/2018	19/07/2018	.	Grade 2 (moderate)	Yes	No reasonable possibility	Not applicable		No	No	Not recovered/ Not resolved
057-012	4	Pyrexia	15/07/2018	17/07/2018	.	Grade 2 (moderate)	Yes	No reasonable possibility	Not applicable		No	No	Recovered/ Resolved
057-012	5	Nausea	16/07/2018	16/07/2018	.	Grade 1 (mild)	No	No reasonable possibility	Not applicable		No	No	Recovered/ Resolved
057-012	6	Vomiting	16/07/2018	16/07/2018	.	Grade 1 (mild)	No	No reasonable possibility	Not applicable		No	No	Recovered/ Resolved
057-012	7	Chest pain	16/07/2018	25/07/2018	.	Grade 2 (moderate)	No	No reasonable possibility	Not applicable		No	No	Unknown
057-015	1	Pyrexia	09/10/2018	09/10/2018	.	Grade 1 (mild)	No	No reasonable possibility	Dose not changed		No	No	Recovered/ Resolved
057-015	2	Hypotension	10/10/2018	11/10/2018	.	Grade 1 (mild)	No	No reasonable possibility	Dose not changed		No	No	Recovered/ Resolved
057-015	3	Diarrhoea	18/10/2018	02/11/2018	.	Grade 1 (mild)	No	No reasonable possibility	Dose not changed		No	No	Recovered/ Resolved
057-015	4	Back pain	18/10/2018	18/10/2018	.	Grade 1 (mild)	No	No reasonable possibility	Dose not changed		No	No	Recovered/ Resolved
057-015	5	Jugular vein thrombosis; Brachiocephalic vein thrombosis; Subclavian vein thrombosis	30/10/2018	08/11/2018	.	Grade 2 (moderate)	Yes	Reasonable possibility	Drug permanently withdrawn		No	No	Recovered/ Resolved
057-015	6	Lipase increased	08/10/2018	08/10/2018	.	Grade 3 (severe)	No	No reasonable possibility	Dose not changed		No	No	Recovered/ Resolved

ZKS Münster SAS-program: tTF_NGR_AE_Listing_V1.3.sas Date of export: 16.12.2019

tTF-NGR Phase I Study EudraCT-No.: 2016-003042-85

Line listing of adverse events (AEs) as reported in eCRF at 16.12.2019

Patient ID	AE No.	MedDRA preferred term (v22.1)	Date of onset of AE	Date of end of AE	or ongoing	CTCAE Grading (v 4.0)	serious (=SAE)	Relationship to tTF-NGR	Action taken with tTF-NGR	Comment on action taken with tTF-NGR	AE considered as DLT by investi-gator	AE that caused dis-continuation of whole study	Outcome
057-015	7	Lipase increased	16/10/2018	16/10/2018	.	Grade 1 (mild)	No	No reasonable possibility	Dose not changed		No	No	Recovered/ Resolved
057-015	8	Amylase increased	08/10/2018	08/10/2018	.	Grade 1 (mild)	No	No reasonable possibility	Dose not changed		No	No	Recovered/ Resolved
057-015	9	Blood creatinine increased	29/10/2018	30/10/2018	.	Grade 2 (moderate)	No	No reasonable possibility	Dose not changed		No	No	Recovered/ Resolved
057-015	10	Blood creatinine increased	08/11/2018	08/11/2018	.	Grade 2 (moderate)	No	No reasonable possibility	Not applicable		No	No	Recovered/ Resolved
057-015	11	Fibrin D dimer increased	30/10/2018	30/10/2018	.	Grade 2 (moderate)	No	Reasonable possibility	Dose not changed		No	No	Recovered/ Resolved
057-015	12	Anaemia	30/10/2018	30/10/2018	.	Grade 1 (mild)	No	No reasonable possibility	Dose not changed		No	No	Recovered/ Resolved
057-015	13	Hyperuricaemia	08/11/2018	08/11/2018	.	Grade 1 (mild)	No	No reasonable possibility	Not applicable		No	No	Recovered/ Resolved
057-015	14	Hypercalcaemia	08/11/2018	08/11/2018	.	Grade 1 (mild)	No	No reasonable possibility	Not applicable		No	No	Recovered/ Resolved
057-016	1	Abscess; Sepsis	12/11/2018	14/11/2018	.	Grade 5 (death)	Yes	No reasonable possibility	Not applicable	not given at all	No	No	Fatal
057-018	1	Troponin T increased	29/01/2019	31/01/2019	.	Grade 1 (mild)	No	Reasonable possibility	Dose not changed		No	No	Not recovered/ Not resolved
057-018	2	Troponin T increased	31/01/2019	06/02/2019	.	Grade 3 (severe)	Yes	Reasonable possibility	Drug permanently withdrawn		Yes	No	Recovered/ Resolved
057-018	3	Lower gastrointestinal haemorrhage	03/02/2019	06/02/2019	.	Grade 2 (moderate)	Yes	No reasonable possibility	Not applicable		No	No	Recovered/ Resolved
057-019	1	Pyrexia	14/02/2019	14/02/2019	.	Grade 1 (mild)	No	No reasonable possibility	Dose not changed		No	No	Recovered/ Resolved
057-019	2	Transient ischaemic attack	04/03/2019	05/03/2019	.	Grade 2 (moderate)	Yes	Reasonable possibility	Drug permanently withdrawn		No	No	Recovered/ Resolved

tTF-NGR Phase I Study EudraCT-No.: 2016-003042-85

Line listing of adverse events (AEs) as reported in eCRF at 16.12.2019

Patient ID	AE No.	MedDRA preferred term (v22.1)	Date of onset of AE	Date of end of AE	or ongoing	CTCAE Grading (v 4.0)	serious (=SAE)	Relationship to tTF-NGR	Action taken with tTF-NGR	Comment on action taken with tTF-NGR	AE considered as DLT by investi-gator	AE that caused dis-continuation of whole study	Outcome
057-019	3	Troponin T increased	15/02/2019	18/02/2019	.	Grade 1 (mild)	No	Reasonable possibility	Dose not changed		No	No	Recovered/ Resolved
057-020	1	Diarrhoea	07/04/2019	08/04/2019	.	Grade 1 (mild)	No	No reasonable possibility	Dose not changed		No	No	Recovered/ Resolved
057-020	2	Pyrexia	11/04/2019	13/04/2019	.	Grade 2 (moderate)	No	No reasonable possibility	Dose not changed		No	No	Recovered/ Resolved
057-020	3	Device related infection	14/04/2019	30/04/2019	.	Grade 3 (severe)	Yes	No reasonable possibility	Dose not changed		No	No	Recovered/ Resolved
057-021	1	Troponin T increased	16/04/2019	25/04/2019	.	Grade 3 (severe)	Yes	Reasonable possibility	Drug permanently withdrawn		Yes	No	Recovered/ Resolved
057-022	1	Head discomfort	03/06/2019	04/06/2019	.	Grade 1 (mild)	No	No reasonable possibility	Dose not changed		No	No	Recovered/ Resolved
057-022	2	Hyperhidrosis	08/06/2019	10/06/2019	.	Grade 2 (moderate)	No	No reasonable possibility	Dose not changed		No	No	Recovered/ Resolved
057-022	3	Thrombocytopenia	28/06/2019	28/06/2019	.	Grade 1 (mild)	No	Reasonable possibility	Dose not changed		No	No	Recovered/ Resolved
057-023	1	Thrombocytopenia	08/07/2019	17/07/2019	.	Grade 2 (moderate)	No	Reasonable possibility	Dose not changed		No	No	Recovered/ Resolved
057-024	1	Ataxia	31/10/2019	06/11/2019	.	Grade 3 (severe)	Yes	No reasonable possibility	Not applicable		No	No	Recovering/ Resolving

Troponin T in Table S1 stands for Troponin T hs, which was actually measured.

Table S2. Absolute doses of tTF-NGR given per patients.

Suppl. Table 2: Absolute doses of tTF-NGR given per patient

						Absolute Dose [mg] per Day per Patient														
UPN	Birthday	Gender	tTF-NGR batch no.	Dose [mg/m²]	Body Surface Area (BSA,m²)	C1 d1	C1 d2	C1 d3	C1 d4	C1 d5	C2 d1	C2 d2	C2 d3	C2 d4	C2 d5	C3 d1	C3 d2	C3 d3	C3 d4	C3 d5
**057-001**	23.06.1968*	m	–	–**	–	–***	–	–	–	–	–	–	–	–	–	–	–	–	–	–
**057-002**	16.12.79	m	–	–	–	–	–	–	–	–	–	–	–	–	–	–	–	–	–	–
**057-003**	10.08.96	m	29, 30	C1: 1.0 / C2: 1.5	C1: 1.94 / C2: 1.94	1.94	1.94	1.94	1.94	1.94	2.91****	2.91	2.91	2.91	2.91	–	–	–	–	–
**057-004**	30.05.90	f	29, 30	C1: 1.5 / C2: 2.0	C1: 1.6 / C2: 1.59***	2.40	2.40	2.40	2.40	2.40	3.20	3.20	3.20	3.20	3.20	–	–	–	–	–
**057-005**	07.02.54	m	29, 30	C1: 2.0 / C2: 2.5	C1: 2.1 / C2: 2.1	4.20	4.20	4.20	4.20	4.20	5.25	5.25	5.25	5.25	5.25	–	–	–	–	–
**057-006**	05.12.72	m	31, 33, 34	C1: 2.5 / C2: 3.0	C1: 2.2 / C2: 2.2	5.50	5.50	5.50	5.50	5.50	6.60	6.60	6.60	6.60	6.60	–	–	–	–	–
**057-007**	19.02.60	m	29, 31, 34	C1: 3.0 / C2: 4.0 / C3: 5.0	C1: 2.0 / C2: 2.0 / C3: 2.0	6.00	6.00	6.00	6.00	6.00	8.00	8.00	8.00	8.00	8.00	10.00	10.00	10.00	10.00	10.00
**057-008**	25.12.72	m	29, 31	C1: 5.0 / C2: -	C1: 1.9 / C2: -	9.50	9.50	9.50	–	–	–	–	–	–	–	–	–	–	–	–
**057-009**	19.11.62	m	31	C1: 5.0 / C2: -	C1: 1.9 / C2: -	9.50	–	–	–	–	–	–	–	–	–	–	–	–	–	–
**057-010**	18.05.60	f	–	–	–	–	–	–	–	–	–	–	–	–	–	–	–	–	–	–
**057-011**	17.01.44	m	33	C1: 4.0 / C: -	C1: 1.8 / C2: -	7.20	7.20	–	–	–	–	–	–	–	–	–	–	–	–	–
**057-012**	29.04.93	m	33	C1: 4.0 / C2: -	C1: 2.0 / C2: -	8.00	8.00	8.00	8.00	8.00	–	–	–	–	–	–	–	–	–	–
**057-013**	01.08.61	m	–	–	–	–	–	–	–	–	–	–	–	–	–	–	–	–	–	–
**057-014**	19.10.56	m	–	–	–	–	–	–	–	–	–	–	–	–	–	–	–	–	–	–
**057-015**	09.06.56	m	34	C1: 4.0 / C2: 4.0	C1: 1.9 / C2: 1.89	7.60	7.60	7.60	7.60	7.60	7.56	7.56	7.56	7.56	7.56	–	–	–	–	–
**057-016**	11.10.55	m	–	–	–	–	–	–	–	–	–	–	–	–	–	–	–	–	–	–
**057-017**	16.07.74	m	–	–	–	–	–	–	–	–	–	–	–	–	–	–	–	–	–	–
**057-018**	17.06.64	f	33	C1: 4.0 / C2: -	C1: 1.84 / C2: -	7.40	7.40	7.40	–	–	–	–	–	–	–	–	–	–	–	–
**057-019**	09.12.77	f	33, 34	C1: 3.0 / C2: 3.0	C1: 1.7 / C2: 1.7	5.10	5.10	5.10	5.10	5.10	5.10	–	–	–	–	–	–	–	–	–
**057-020**	04.12.99	m	33, 34	C1: 3.0 / C2: 3.0	C1: 1.7 / C2: 1.7	5.10	5.10	5.10	5.10	5.10	5.10	5.10	5.10	5.10	5.10	–	–	–	–	–
**057-021**	19.06.55	m	34	C1: 3.0 / C2: -	C1: 1.83 / C2: -	5.50	–	–	–	–	–	–	–	–	–	–	–	–	–	–
**057-022**	14.11.62	f	36, 37	C1: 3.0 / C2: 3.0	C1: 1.76 / C2: 1.76/1.79	5.28	5.28	5.28	5.28	5.28	5.28	5.28	5.37	5.37	5.28	–	–	–	–	–
**057-023**	02.01.59	m	36, 37	C1: 3.0 / C2: 3.0	C1: 1.74 / C2: 1.74/1.76	5.22	5.22	5.22	5.22	5.22	5.22	5.22	5.28	5.28	5.28	–	–	–	–	–
**057-024**	07.06.95	m	38, 39	C1: 3.0 / C2: 3.0	C1: 1.79 / C2: 1.80	5.37	5.37	5.37	5.37	5.37	5.40	5.40	5.40	5.40	5.40	–	–	–	–	–

*****, day.month.year; ******, screening failure; *******, not given; ********, changes according to actual BSA

UPN, unique patient number, here with study number 057; **C**, cycle; **d**, day; **f**, female; **m**, male

Table S3. Frequency of SUSAR and SAE possibly related to tTF-NGR (MedDRA v22.1).

**Observation**	**MeDRA preferred term**	**n[1]**	**Maximum dose level tTF-NGR[1]**	**UPN**	**Maximum CTCAE 4.0 grade**
1	Transient ischaemic attack	1	3	019	2, moderate
2	Troponin T[3] increased	1	3	021	3, severe
3[4]	Brachiocephalic vein thrombosis	1	4	015	2, moderate
4[4]	Jugular vein thrombosis	1	4	015	2, moderate
5[4]	Subclavian vein thrombosis	1	4	015	2, moderate
6	Troponin T increased	2	4	011, 018	3, severe
7	Deep vein thrombosis	1	5	008	2, moderate
8	Troponin T increased	2	5	008, 009	3, severe

[1], number of patients with this observation;

[2], dose level in mg/m^2 tTF-NGR/day × 5;

[3], Troponin T relates to Troponin T hs, a term actually not present in CTCAE 4.0, further comments see results and discussion;

[4], observation 3, 4, and 5 relate to the identical patient UPN 015, who developed a catheter-related thrombosis partially involving all 3 veins (see results in the manuscript).

Table S4. tTF-NGR phase I study - CEUS maximum and minimum A.U. values for wash-in perfusion index in all patients with measurable lesions.

UPN - diagnosis	Measurement *wash-in perfusion index (PI)*	region of interest	tTF-NGR dose in mg/m² x 5	prior to tTF-NGR cycle 1	post/during tTF-NGR cycle 1	between cycles 1 and 2	prior to tTF-NGR cycle 2	post tTF-NGR cycle 2
003 - germ cell cancer	*wash-in PI*	liver metastasis	1.5	506.9	52.9			
	wash-in PI	liver reference	1.5	117.4	86.7			
004 - hepatocellular carcinoma	*wash-in PI*	metastasis intra- and extra-hepatically	cycle 1: 1.5<br>cycle 2: 2.0	ROI 1: 283.6<br>ROI 2: 522.4	249.7<br>515.5	126.9<br>338.4	254.1<br>679.7	124.3<br>386.0
	wash-in PI	liver reference	cycle 1: 1.5<br>cycle 2: 2.0	54.9	80.3	82.5	96.1	75.9
006 - colorectal carcinoma	*wash-in PI*	liver metastasis<br>ROI 2 in ROI 1	2.5	ROI 1: 102.7<br>ROI 2: 65.2	9.3<br>3.0			
	wash-in PI	liver reference	2.5	47.8	65.8			
011 - colorectal carcinoma	*wash-in PI*	liver metastasis	4.0, appl. only days 1 and 2	lesion 1: 1517.5<br>total lesion: 541.0	1465.9<br>773.6			
	wash-in PI	liver reference	4.0, appl. only days 1 and 2	62.3	339.6			
	wash-in PI in % of liver reference	liver metastasis<br>ROI 2 in ROI 1		lesion 1: 2446 %<br>total lesion: 872 %	423 %<br>228 %			
012 - germ cell cancer	*wash-in PI*	liver metastasis	cycle 1: 4.0	total lesion: 103.9	total lesion (d3): 56.2			
		liver reference	cycle 1: 4.0	50.8	d3: 72.1			
015 – medullary thyroid cancer	*wash-in PI*	lnn metastasis neck	4.0	total lesion: 9.63<br>lower half: 8.17<br>small hyperperfused ROI: 7.50	0.86<br>1.11<br><br>4.94		4.32<br>9.06<br><br>6.35	
019 - angiosarcoma	*wash-in PI %*	liver metastasis	3.0	total lesion 562.3 %<br>ref lesion: 1520.7 %	d1: 355.1 %<br>d1: 705.0 %	d5: 443.5 %<br>d5: 781.1 %		
	wash-in PI %	liver reference	3.0	100 %	100%	100 %		
020 – small round cell tumor	*wash-in PI*	liver metastasis	3.0	total lesion: 77.2<br>central lesion: 56.9<br>upper part: 12.6	d1: 66.0<br>d1: 39.0<br>d1: 6.4	159.9<br>119.6<br>17.8	243.7<br>182.3<br>5.7	176.5<br>213.6<br>3.5 (necrosis)
	wash-in PI	liver reference	3.0	128.0	d1: 110.7	147.5	148.9	152.8
022 – hepatocellular carcinoma	*wash-in PI*	liver lesion	3.0	total lesion: 233.2<br>central lesion: 246.4	61.3<br>52.7		181.0<br>106.5	83.0<br>51.4
	wash-in PI	liver reference	3.0	not available (NA)	NA		NA	NA
023 – colorectal adenocarcinoma	*wash-in PI*	liver metastasis	3.0	total lesion: 64.8<br>central lesion: 43.2	26.7<br>5.7		31.1<br>30.6	d1: 11.2<br>d1: 7.7
		Liver reference	3.0	NA	NA		NA	NA

CEUS, contrast enhanced ultrasound; A.U., arbitrary units; d=day; lnn, lymph nodes
Wash-in Perfusion Index (PI): Wash-in Area Under the Curve/Rise Time (see Suppl. Figure 1);
Patients 001, 002, 010, 011, 013, 014, 016, 017 were screening failures and/or not treated, patients 005, 007, 008, 009, and 024 had no lesions measurable by CEUS, patients 008 and 018 had a DLT,
Pat 019: variable blood flow, thus a.u. given also as percentage of normal liver reference lesion,
NA, not available

Table S5. VVF and k-trans in magnetic resonance imaging under tTF-NGR application.

**VVF**	**UPN**									
arbitrary units		**VVF**								
		1st Cycle			2nd Cycle			3rd Cycle		
		Pre	5h	Post	Pre	5h	Post	Pre	5h	Post
	0 0 1									
	0 0 2									
	0 0 3	-	-	-	-	-	-	-	-	-
	0 0 4	-	-	-	-	-	-	-	-	-
	0 0 5	-	-	-	-	-	-	-	-	-
	0 0 6	1.62	0.73	0.48	1.39	0.43	0.16	-	-	-
	0 0 7	1.51	0.93	0.4	0.49	0.38	0.3	0.64	0.54	0.83
	0 0 8	1.65	0.52	0.79	-	-	-	-	-	-
	0 0 9	1.01	0.61	1.46	-	-	-	-	-	-
	0 1 0									
	0 1 1	3.18	1.65	2.6	-	-	-	-	-	-
	0 1 2	2.63	0.26	0.22	-	-	-	-	-	-
	0 1 3									
	0 1 4									
	0 1 5	3.68	1.97	1.89	1.77	1.19	1.62	-	-	-
	0 1 6									
	0 1 7									
	0 1 8	2.93	0.8	2.59	-	-	-	-	-	-
	0 1 9	4.22	2.53	2.46	-	-	-	-	-	-
	0 2 0	2.41	1.11	0.96	1.99	1.27	0.75			
	0 2 1	4.07	2.82	2.21	-	-	-	-	-	-
	0 2 2	3.71	2.59	2.65	3.44	1.84	1.91	-	-	-
	0 2 3	2.71	0.93	0.84	1.52	-	1.08	-	-	-
	0 2 4	0.73	0.22	0.20	2.95	2.31	1.41			
**k-trans**										
10 e-3/min		**k-trans**								
		1st Cycle			2nd Cycle			3rd Cycle		
		Pre	5h	Post	Pre	5h	Post	Pre	5h	Post
	0 0 1									
	0 0 2									
	0 0 3	366.7	251.61	135.87	-	63.18	51.87	-	-	-
	0 0 4	115.58	85.41	37.57	241.74	-	139.08	-	-	-
	0 0 5	-	-	-	-	-	-	-	-	-
	0 0 6	-	-	-	-	-	-	-	-	-
	0 0 7	-	-	-	15.13	2.77	-	-	-	-
	0 0 8	491.26	220.76	366.63	-	-	-	-	-	-
	0 0 9	65.31	27.19	38.36	-	-	-	-	-	-
	0 1 0									
	0 1 1	253.3	194.33	207.48				-	-	-
	0 1 2	45.82	39.33	25.5	-	-	-	-	-	-
	0 1 3									
	0 1 4									
	0 1 5	320.76	194.39	167.9	258.35	71.6	98.72	-	-	-
	0 1 6									
	0 1 7									
	0 1 8	70.78	53.14	65.11	-	-	-	-	-	-
	0 1 9	204.72	158.2	125.45	-	-	-	-	-	-
	0 2 0	148.72	87.86	98,14	111.58	72.86	59.07			
	0 2 1	296.13	171.59	173.76	-	-	-	-	-	-
	0 2 2	193.38	186.93	175.21	177.08	160.79	149.7	-	-	-
	0 2 3	134.06	113.6	94.52	96.52	-	41.61	-	-	-
	0 2 4	60.89	34.87	22.77	123.01	85.44	74.95			

VVF, vascular volume fraction; **UPN**, unique patient number; **Pre**, before application of tTF-NGR; **5h**, approx. 5 hours after tTF-NGR; **Post**, at end of cycle; **-**, not applicated or not measured
empty rows, patient screening failures

Table S6. CD13 immunohistochemistry (IHC) staining intensity per patient and distribution according to histology.

Staining Location		Vascular/Perivascular	Tumor Cells	Composite Stain*
**Patient UPN****	**Diagnosis****	***		
003	germ cell	n.d.	n.d.	n.d.
004	HCC	1	2	2
005	SCLC	1	0	1
006	rectal adeno	2	0	2
007	colon adeno	1	0	1
008	synovial sarcoma	2	0	2
009	squamous lung	2	0	2
011	colon adeno	1	0	1
012	germ cell	3	1	3
015	medullary thyroid	1	0	1
018	CUP	2	0	2
019	angiosarcoma	2	1	2
020	desmoplastic round cell	n.d.	n.d.	n.d.
021	synovial sarcoma	3	3	3
022	HCC	n.d.	n.d.	n.d.
023	colon adeno	n.d.		
024	undifferentiated pleomorphic sarcoma	2	3	3

*, composite indicates the highest observed intensity of either vascular/perivascular or tumor cell per case;
**, for histology and disease history see Table 1;
***, intensity scores according to the method published (see ref. 20);
n.d., not done; HCC, hepatocellular carcinoma; SCLC, small-cell lung cancer;
CUP, cancer of unknown primary

Table S7. Non-compartment analysis of tTF-NGR with various intravenous doses of tTF-NGR administered to cancer patients. **(**For Tables S7 A–C: For explanation and description of values and methodology see Methods. UPN, unique patient number; c, cycle; h, hour; term., terminal)

Table S7. A. Non-compartment analysis of tTF-NGR with various intravenous doses of tTF-NGR administered to cancer patients (all data; some data reappear in verification groups).

Non-compartment analysis of tTF-NGR, test day 1 (all values up to 24 hours after start of 1st infusion)								
**Dose [mg/m²]**	**Pat.-UPN /cycle**	**C_{max}[#] [ng/mL]**	**t_{max}[#] [h]**	**$t_{1/2}$ (term.) [h]**	**K_{el} [1/h]**	**$AUC_{0\text{-}t\ last}$ [ng*h/mL]**	**time interval for $t_{1/2}$ determination [h][#]**	**$AUC_{0\text{-}t\ last}$/dose [h*m²*ng/mL/mg]**
**1.0**	**003/c1**	**138.10**	**1.0**	**7.34**	**0.094**	**285.54**	3.5–24.0	**285.54**
**1.5**	**003/c2**	227.45	1.0	6.02	0.115	513.00	3.5–24.0	342.00
	**004/c1**	295.95	1.0	5.62	0.123	556.37	3.0–24.0	370.91
	**mean**	**261.70**	**1.0**	**5.82**	**0.119**	**534.69**	–	**356.46**
**2.0**	**004/c2**	354.28	1.0	4.86	0.143	682.47	3.5–24.0	341.24
	**005/c1**	328.31	1.0	5.15	0.135	1132.14	3.5–24.0	566.07
	**mean**	**341.30**	**1.0**	**7.4**	**0.139**	**907.31**	–	**453.66**
**2.5**	**005/c2**	412.64	1.0	8.07	0.086	1492.83	3.5–24.0	597.13
	**006/c1**	459.46	1.0	7.03	0.099	1597.85	3.5–24.0	1031.12
	**mean**	**436.05**	**1.0**	**7.55**	**0.093**	**1545.34**	–	**618.14**
**3.0**	**006/c2**	534.33	1.0	9.73	0.071	2608.60	3.5–24.0	869.53
	**007/c1**	613.45	1.0	6.84	0.101	2404.00	3.5–24.0	801.33
	**019/c1**	930.96	1.0	9.59	0.072	2942.32	3.0–24.0	980.77
	**019/c2**	1312.39	1.0	11.07	0.063	2980.56	3.5–24.0	993.52
	**020/c1**	730.09	1.0	8.38	0.083	2205.38	3.5–24.0	735.13
	**020/c2**	528.64	1.0	10.04	0.069	1914.98	3.0–24.0	638.33
	**021/c1**	694.56	1.0	9.49	0.073	3197.90	3.5–24.0	1065.97
	**022/c1**	549.72	1.0	7.51	0.092	1940.19	3.5–24.0	646.73
	**022/c2**	717.03	1.0	11.09	0.063	2926.03	3.0–24.0	975.34
	**023/c1**	647.0	1.0	7.31	0.095	1802.17	3.5–24.0	600.72
	**023/c2**	577.29	1.0	11.11	0.062	2038.59	3.0–24.0	679.53
	**024/c1**	523.96	1.0	7.47	0.093	1977.68	3.0–24.0	659.23
	**024/c2**	548.69	1.0	7.29	0.095	1822.53	3.0–24.0	607.51
	**mean**	**685.24**	**1.0**	**8.99**	**0.079**	**2366.23**	–	**788.74**
**4.0**	**007/c2**	1129.27	1.5	8.73	0.079	3311.11	3.5–24.0	827.78
	**011/c1**	1085.72	1.0	7.90	0.088	4446.09	3.5–24.0	1111.52
	**012/c1**	1242.88	1.0	8.57	0.081	3512.46	5.5–24.0	878.12
	**015/c1**	1763.35	1.0	6.10	0.110	6628.61	3.0–24.0	1657.15
	**015/c2**	1672.61	1.0	7.63	0.091	8368.98	3.5–24.0	2092.25
	**018/c1**	840.79	1.0	7.85	0.088	2657.41	3.5–24.0	664.35
	**mean**	**1289.10**	**1.08**	**7.80**	**0.090**	**4820.78**	–	**1205.20**
**5.0**	**007/c3**	1089.79	1.0	6.08	0.114	5082.18	3.5–24.0	1016.44
	**008/c1**	1770.21	1.5	5.27	0.132	5089.87	3.5–24.0	1017.97
	**009/c1**	1001.43	1.0	7.13	0.097	4107.33	3.5–24.0	821.47
	**mean**	**1287.14**	**1.17**	**6.16**	**0.114**	**4759.79**	–	**951.96**

#, values obtained from plasma analysis, all other values calculated by toxicokinetic analysis

Table S7. B. Non-compartment analysis of tTF-NGR with intravenous doses of 4.0 mg/m². (4.0 mg/m²-verification group; values are also in Table S7. A).

Non-compartment analysis of tTF-NGR, test day 1 (up to 24 h after start of 1st infusion of each cycle)								
**Dose [mg/m²]**	**Pat.-UPN/cycle**	**C_{max}[#] [ng/mL]**	**t_{max}[#] [h]**	**$t_{1/2}$ (term.) [h]**	**K_{el} [1/h]**	**$AUC_{0\text{-}t\ last}$ [ng*h/mL]**	**time interval for $t_{1/2}$ determination [h]**[#]	**$AUC_{0\text{-}t\ last}$/dose [h*m²*ng/mL/mg]**
**4.0**	**007/c2**	1129.27	1.5	8.73	0.079	3311.11	3.5–24.0	827.78
	**011/c1**	1085.72	1.0	7.90	0.088	4446.09	3.5–24.0	1111.52
	**012/c1**	1242.88	1.0	8.57	0.081	3512.46	5.5–24.0	878.12
	**015/c1**	1763.35	1.0	6.10	0.110	6628.61	4.0–24.0	1657.15
	**015/c2**	1672.61	1.0	7.63	0.091	8368.98	3.5–24.0	2092.25
	**018/c1**	840.79	1.0	7.85	0.088	2657.41	3.5–24.0	664.35
	**mean**	**1289.10**	**1.08**	**7.80**	**0.090**	**4820.78**	–	**1205.20**

#, values obtained from plasma analysis, all other values calculated by toxicokinetic analysis

Table 7. C. Non-compartment analysis of tTF-NGR with intravenous doses of 3.0 mg/m². (3.0 mg/m²-verification group; values are also in Table S7. A).

Non-compartment analysis of tTF-NGR, test day 1 (up to 24 h after start of 1st infusion of each cycle)								
**Dose [mg/m²]**	**Pat.-UPN/cycle**	**C_{max}[#] [ng/mL]**	**t_{max}[#] [h]**	**$t_{1/2}$ (term.) [h]**	**K_{el} [1/h]**	**$AUC_{0\text{-}t\ last}$ [ng*h/mL]**	**time interval for $t_{1/2}$ determination [h]**[#]	**$AUC_{0\text{-}t\ last}$/dose [h*m²*ng/mL/mg]**
**3.0**	**006/c2**	534.33	1.0	9.73	0.071	2608.60	3.5–24.0	869.53
	**007/c1**	613.45	1.0	6.84	0.101	2404.00	3.5–24.0	801.33
	**019/c1**	930.96	1.0	9.59	0.072	2942.32	3.0–24.0	980.77
	**019/c2**	1312.39	1.0	11.07	0.063	2980.56	3.5–24.0	993.52
	**020/c1**	730.09	1.0	8.38	0.083	2205.38	3.5–24.0	735.13
	**020/c2**	528.64	1.0	10.04	0.069	1914.98	3.0–24.0	638.33
	**021/c1**	694.56	1.0	9.49	0.073	3197.90	3.5–24.0	1065.97
	**022/c1**	549.72	1.0	7.51	0.092	1940.19	3.5–24.0	646.73
	**022/c2**	717.03	1.0	11.09	0.063	2926.03	3.0–24.0	975.34
	**023/c1**	647.0	1.0	7.31	0.095	1802.17	3.5–24.0	600.72

	**023/c2**	577.29	1.0	11.11	0.062	2038.59	3.0–24.0	679.53
	**024/c1**	523.96	1.0	7.47	0.093	1977.68	3.0–24.0	659.23
	**024/c2**	548.69	1.0	7.29	0.095	1822.53	3.0–24.0	607.51
	**mean**	**685.24**	**1.0**	**8.99**	**0.079**	**2366.23**	–	**788.74**

#, values obtained from plasma analysis, all other values calculated by toxicokinetic analysis.
